# Predictors of infant birth weights: Role of the Lebanese mediterranean diet, psychosocial factors and maternal health status

**DOI:** 10.1371/journal.pone.0351497

**Published:** 2026-06-10

**Authors:** Paula Hage Boutros, Jessy El Hayek Fares, Maya Bassil, Kristine G. Koski

**Affiliations:** 1 Department of Nutrition, Faculty of Health Sciences, Modern University for Business and Science, Beirut, Lebanon; 2 Faculty of Nursing and Health Sciences, Notre Dame University- Louaize (NDU), Zouk Mosbeh, Lebanon; 3 Department of Nutrition Sciences, College of Health Sciences, QU Health, Qatar University, Doha, Qatar; 4 School of Human Nutrition McGill University - Macdonald Campus, Ste Anne de Bellevue Quebec, Canada; King Saud University Medical City, SAUDI ARABIA

## Abstract

**Background:**

The Mediterranean diet (MeD) is associated with favorable pregnancy outcomes, but contribution of this dietary pattern during pregnancy with small (SGA), appropriate (AGA) and large (LGA) for gestational age births is limited.

**Methods:**

For this prospective national cohort study 618 Lebanese pregnant women were recruited. Infant birth weight was categorized into SGA (n = 73), AGA (n = 447) and LGA (n = 98). Modifiers of birth weight outcomes included dietary adherence to Lebanese MeD (LMeD), trimestral and total weight gain, MAP (mean arterial pressure) and PP (pulse pressure) and psychosocial, socio-demographic, and maternal health factors. Descriptive statistics compared differences among SGA, AGA, and LGA infants. Hierarchical linear regression modeling identified determinants for birth weight categories and hierarchical logistic regression modeling was used to identify factors associated with increasing the likelihood of SGA or LGA compared to AGA births.

**Results:**

Adherence to the LMeD was associated with AGA birth weights where intakes of dairy products were associated with lower normal AGA births and dried fruits with higher normal AGA births. Adherence to LMeD did not enter models for SGA or LGA but intakes of specific foods and maternal health status indicators did. For SGA infants, appropriate gestational weight gain (GWG) mitigated against a low birth weight whereas higher burghul intake in T1 and higher MAP in T2 and T3 were linked to increased SGA risk. For LGA infants, greater parity, previous macrosomia and poor sleep quality in T3, and higher intake of olive oil in T2 were associated with higher risk of a LGA birth whereas higher PP in T1 decreased the odds of a LGA birth.

**Conclusions:**

Screening for family history of diabetes and macrosomia, targeting trimester-specific gestational weight gain, monitoring maternal blood pressure, pulse pressure, and sleep quality, and promoting adherence to the Lebanese Mediterranean diet are important strategies to optimize infant birth outcomes.

## Introduction

Eastern Mediterranean countries (EMCs) are undergoing a rapid nutrition transition driven by economic growth, urbanization, and demographic shifts, leading to reduced adherence to the traditional Mediterranean diet (MeD) and increased adoption of Western dietary patterns and sedentary lifestyles [1]. These changes are particularly relevant for women of childbearing age, as maternal diet before and during pregnancy plays a key role in fetal growth and infant birth weight [2–4].

Adherence to the MeD—characterized by high intake of fruits, vegetables, whole grains, legumes, and olive oil—has been consistently associated with favorable pregnancy outcomes, including optimal birth weight and reduced risk of small for gestational age (SGA) and large for gestational age (LGA) infants [5–7]. Individual components such as fruits, vegetables, dairy products, and extra virgin olive oil have also been linked to improved fetal growth [8, 9], whereas poor adherence, particularly in early pregnancy, has been associated with impaired placental development and lower birth weight [10]. However, most studies assess diet at single timepoints, with limited evidence on adherence across pregnancy [11].

Maternal anthropometrics and clinical conditions are also key determinants of birth weight. Both excessive and inadequate gestational weight gain (GWG) are associated with adverse outcomes, including macrosomia and low birth weight [12–14]. Metabolic disturbances such as pregnancy-induced insulin resistance may increase the risk of both SGA and LGA infants [15,16]. Hypertensive disorders of pregnancy (HDPs) and gestational diabetes mellitus (GDM) further influence fetal growth through impaired placental function and altered glucose metabolism [17–19], particularly in low- and middle-income countries (LMICs) where management may be suboptimal [20].

Psychosocial factors, including maternal stress, depression, and sleep disturbances, have also been associated with adverse outcomes such as low birth weight, preterm birth, and impaired neurodevelopment, potentially mediated through inflammatory and neuroendocrine pathways [21,22].

Given the multifactorial determinants of birth weight and the limited evidence from Mediterranean populations, this study aims to: (1) identify predictors of AGA, SGA, and LGA infants, including maternal anthropometrics, GWG, and psychosocial factors; (2) assess the association between adherence to the Lebanese Mediterranean Diet (LMeD) and birth weight categories; and (3) evaluate the contribution of specific LMeD components to birth outcomes.

## Materials and methods

### Study participants

This national prospective cohort study recruited pregnant women from the 6 different governorates of Lebanon that included Mount Lebanon (30.7%), Beirut (12.4%), Bekaa (16.4%) South Lebanon (and Nabatieh) (29.4%), North Lebanon (7.4%) and Akkar (3.7%.) The inclusion criterion was pregnant Lebanese women > 18 years with a singleton pregnancy. The exclusion criteria were women 1) with multiple gestations, 2) prepregnancy diabetes, and 3) those carrying a fetus with structural malformation, chromosomal anomalies or TORCH (toxoplasmosis, rubella, cytomegalovirus, herpes and other agent) infections.

We used Epiinfo software to calculate the sample size needed via the Fleiss formula with correction [23]. The confidence interval was set at 95%, the power at 80% and the ratio (unexposed/exposed to the MeD = 1). A sample of 618 participants was targeted after adjusting for a 20% loss to follow-up. Simple random sampling among all obstetric clinics in Lebanon was performed with SPSS version 22.0 (Statistical Package for Social Sciences yr). Among the 732 obstetric clinics that were retrieved from the Syndicate of Physicians in Lebanon, a total of 20 private and hospital-based private clinics were stratified by governorate, and the sample was proportionally allocated to include 15 private clinics and 5-hospital based clinics. The following distribution was made across the governorates: Mount Lebanon (n = 3, 185 women), Beirut (n = 5, 105), Bekaa (n = 4, 99), South Lebanon (n = 3,167), North Lebanon (n = 4, 43), and Akkar (n = 1, 19), with a minimum of 30 women recruited from each clinic. Participants were consecutively recruited within each clinic at their first prenatal visit during the study period. This refers to women presenting for their initial antenatal consultation for the current pregnancy, all of whom were in their first trimester (<12 weeks of gestation) at the time of enrollment.

This study was conducted in accordance with the ethical principles of the Declaration of Helsinki. Ethical approval was obtained from the Bellevue Medical Center Institutional Review Board (IRB). In addition, written permission was secured from the supervising physician or head of each participating private clinic, allowing the research team to conduct the survey and access relevant medical records. Recruitment took place between December 15, 2019, and June 30, 2021. Healthy pregnant women (<15 weeks gestation) attending routine obstetric visits were approached by trained research staff, provided with detailed information about the study, and invited to participate. Written informed consent was obtained from all participants, who were assured that participation was voluntary and that they could withdraw at any time without any consequences to their medical care.

### Study design

Pregnant women were followed from the 1st trimester (<15 weeks of gestation) until delivery, with follow-up interviews in the 2nd (24--28 weeks) and 3rd trimesters (34--37 weeks). The initial interview was conducted in person via a socio-demographic questionnaire and 5 validated questionnaires: 1) the Perceived Stress Scale (PSS10) [24], 2) the Pittsburgh Sleep Quality Index (PSQI) [25], 3) the Edinsburgh Postnatal Depression Scale (EPDS) [26], 4) the FFQ and LMeD score [27], and 5) the Canadian Physical Activity Readiness Medical Examination (PARmed-X for pregnancy) [28], where we categorized women as sedentary or active. The questionnaires were administered at the first prenatal visit using face-to-face interviews and re-administered in the 2nd and 3rd trimesters of pregnancy via phone interviews.

Clinical data, including self-reported pre-pregnancy weight, vitamin/mineral over the counter supplement intakes, presence of anemia and medical history, were obtained from medical chart reviews. At follow-up visits during each trimester, additional clinical indicators, including weight gain per trimester, blood pressure measurements [systolic blood pressure (SBP), diastolic blood pressure (DBP), mean arterial pressure (MAP) and pulse pressure (PP)] and general maternal health status, were collected from medical charts. MAP and PP were calculated using SBP and DBP. Infant data were collected upon delivery.

### Dietary assessment and adherence to the Lebanese Mediterranean diet

Data on food intake were collected via a previously validated 61-item FFQ specifically for the Lebanese diet by Naja et al., 2015 [27], which is representative of the LMeD and incorporates traditional Lebanese dishes. This tool demonstrated a moderate positive correlation (r = 0.56) with the Italian MeD tool [29]. The FFQ consists of 9 food categories, including 1) breads and cereals, 2) dairy products, 3) fruits and juices, 4) vegetables, 5) meat and alternates, 6) fats and oils, (7) sweets and desserts, 8) beverages, and 9) miscellaneous. The LMeD was developed to assess adherence to a Lebanese version of the MeD. The authors were able to identify an LMeD pattern from the FFQ with the following 9 food groups because of their consistently high loading on this pattern: fruits, vegetables, legumes, olive oil, burghul (crushed whole wheat), milk and dairy products, starchy vegetables (potato, corn and beans), dried fruits and eggs. The LMeD index used in this study shares common food groups with other indices developed in Italy [29], Spain [30], Greece [31], France [32], and 9 Mediterranean European countries [33]; however, in the LMeD, other main components of the traditional Mediterranean diet, such as fish, red meat, poultry, and wine consumption, did not load high on the pattern [27].

Adherence to the LMeD was measured on the basis of the intake of servings of each of the 9 food groups, with 1, 2, and 3 points assigned to consumption in the 1st, 2nd, and 3rd tertiles respectively, and were calculated per trimester. Scores ranged between 9 and 27, with 9 reflecting the least adherence and 27 reflecting the highest adherence to the LMeD.

### Clinical measurements

#### Pre-pregnancy BMI.

Pre-pregnancy BMI data were collected at baseline from obstetrical charts and were used to classify women as underweight (<18.5 kg/m²), normal (18.5–24.9 kg/m²), overweight (25–29.9 kg/m²), or obese (≥30 kg/m²) [34].

#### Total GWG and per trimester GWG.

The total GWG in kg was calculated by subtracting total weight gain from pre-pregnancy weight, and GWG was classified as low, adequate, or excessive according to the pre-pregnancy BMI recommendations of the Institute of Medicine (IOM) (underweight 12.5–18 kg, normal weight 11.5–16 kg, overweight 7–11.5 kg, and obese 5–9 kg) [34]. GWG per trimester was classified as adequate, excessive, or low according to the following recommendations: For the 1st trimester, GWG above 2 kg or below 1 kg was considered excessive or low, respectively, for all categories of BMI. For the 2nd and 3rd trimesters, the recommended weight gain per week for each BMI category is as follows: 0.45–0.6 kg/wk for underweight women, 0.35–0.45 kg/wk for normal weight women, and 0.2–0.3 kg/wk for both overweight and obese women. Weight gain below or above these values was classified as low or excessive, respectively [35].

#### Fasting blood glucose (FBG), impaired glucose tolerance, and diagnosis of GDM.

FBG values were collected from obstetric charts in trimesters 1 and 3 only and were categorized into normal FBG values <5.6 mmol/L and impaired glucose ≥5.6 mmol/L.

During the second trimester visit, GDM diagnosis (yes/no) was made. Obstetricians in Lebanon followed the two-step 50 g oral glucose tolerance test between 28–32 weeks in line with the American College of Obstetricians and Gynecologists for the diagnosis of GDM [36].

#### Mean arterial pressure (MAP) and pulse pressure (PP).

Trimester-specific cutoffs for elevated MAP (eMAP) during pregnancy were defined as >87 mmHg (10– < 18 weeks), > 84 mmHg (18–34 weeks), and >86 mmHg (after 34 weeks) [37]; low MAP was defined as <70 mmHg [38]. MAP is calculated as MAP = DBP + 1/3 (SBP − DBP). PP was calculated as follows: PP = SBP – DBP. Prior research has highlighted that there is no universally accepted definition of “elevated” PP in pregnancy, and thresholds are often derived empirically within individual cohorts rather than standardized across studies [39,40]. In a recent study by Sampson et al., 2024, they chose to use the dichotomous cutoff of PP > 55 mmHg based on previous studies that defined these categories based on the distribution of measurements or means within their respective cohorts [41]. Given this variability and lack of consensus, we opted to retain PP as a continuous variable to preserve statistical power and avoid arbitrary categorization.

Blood pressure measurements were obtained using standardized protocols.

#### Infant birth outcomes.

Birth weight and infant sex were collected upon delivery. Infants with birth weights <10th percentile and >90th percentile according to their gestational age were classified as SGA and LGA, respectively, whereas infants with birth weights between the 10th and 90th percentiles were classified as AGA using the National Center for Health Statistics from 2011 [42]. Gestational age was collected from medical charts based on last menstrual period and confirmed by first-trimester ultrasound.

#### Psychosocial variables.

*Perceived Stress Scale (PSS10)*: Perceived stress was evaluated using the 10-item Perceived Stress Scale (PSS-10) [24]. The Arabic version has been validated among Lebanese pregnant and postpartum women, showing good internal consistency (Cronbach’s alpha = 0.74) and acceptable test–retest reliability (Spearman’s r = 0.49) [34]. The PSS-10 scores were also moderately correlated with the General Health Questionnaire (r = 0.59) and the Edinburgh Postnatal Depression Scale (EPDS) (r = 0.49), indicating concurrent validity [34]. A total score of 0–40 is obtained, with higher scores indicating higher perceived stress. The categories are: low stress (0–13), moderate stress (14–26), and high stress (27–40).

*Pittsburgh Sleep Quality Index (PSQI)*: PSQI is a self-reported tool that assesses sleep quality over a 1-month period. A total score ranges between 0–21. A score of equal or greater to 5 indicates poor sleep, while a score of less than 5 indicates good sleep. It has been validated in an Arabic population with Cronbach’s alpha = 0.74, correlation r = 0.36 and r = 0.19, with the Insomnia Severity Index and Medical Outcome Study Short Form [25].

*Edinburgh Postnatal Depression Scale (EPDS)*: Edinburgh Postnatal Depression Scale (EPDS) is a commonly used 10-item scale in clinical practice to identify women at risk of perinatal depression. A score ranging 0–30 is obtained. A score of 10 or greater indicates possible depression. It has been validated in an Arabic pregnant population with Cronbach’s alpha = 0.84, correlation r = 0.57 with the Present State Examination [26].

### Statistical analyses

Statistical analyses were conducted via SPSS version 25. Descriptive statistics included frequencies and percentages for qualitative data and means, standard deviations, medians, minimums, and maximums for quantitative data, with visual representations via histograms. The dependent variable was infant birth weight, which was categorized into SGA, AGA and LGA using the INTERGROWTH_EFW calculator [43].

Adherence to the LMeD, maternal characteristics and psychosocial variables were compared across AGA, SGA, and LGA birth weight categories by trimesters. Maternal characteristics included pre-pregnancy BMI, smoking, physical activity status (sedentary vs active), GWG, fasting blood glucose (FBG) and evidence of impaired glucose tolerance (IGT), blood pressure measurements (SBP, DBP, MAP, PP), and assessment of stress, sleep, depression. Bivariate analyses were performed using chi-square tests and ANOVA, with significance set at p < 0.05. Significant differences were further explored using post hoc Tukey tests.

Linear regression models were used to assess factors associated with infant BWGA for the study population (n = 618) and for the subset of AGA infants (n = 447) using hierarchical modeling. For Model 1 – maternal characteristics for BWGA- were adjusted for smoking at T1, T2, T3, parity, infant sex, maternal height, pre-pregnancy BMI, family history of diabetes, history of macrosomia, and total GWG. Model 2- Maternal Health Factors – included significant variables from model 1 and further adjusted for GDM, impaired fasting blood glucose≥5.6 mmol/L at T1 and T3, both PP and MAP at T1, T2 and T3, PSQI, and LMeD adherence at T1, T2 and T3. Model 3 incorporated individual food groups in separate models while controlling for significant variables from model 2. Variable selection was conducted using stepwise regression.

Logistic regression was used to evaluate the predictors of SGA and LGA infants each compared to AGA infants. Model 1-Maternal Characteristics – controlled for variables such as family history of diabetes, smoking at T1, T2, T3, parity, infant sex, maternal height, history of macrosomia, both MAP and PP at T1, T2, and T3, PSQI, pre-pregnancy BMI and total GWG, and LMeD adherence at T1, T2 and T3. Model 2- Individual Food Groups included significant variables from Model 1 and individual food groups, with final adjustments made via the forward Wald method.

## Results

### General population and newborn characteristics

Initially, 660 participants were recruited, however 42 dropped out due to miscarriage or not willing to participate making the total sample size to be N = 618. Among the 618 pregnant women; average age was 29.2 ± 5.0 years, average parity was 1.3 children, 49.2% were homemakers and 48.7% were employed. Fewer than half resided in the main urban areas of Mount Lebanon and Beirut, with a greater percentage living in rural regions. The majority had a university degree (76.1%,); 23.9% had a high school education or less. Monthly income ranged from $700--$2000 and varied little by region. Less than 4% of the women smoked in T1, less than 2% smoked in T2 and less than 1.1% smoked in T3. Pre-pregnancy BMI was normal for 63.4% of women; 37.2% were overweight and obese. Excessive weight gain was high for most women with 86.8% in T1 and 93.2% in T3, but most had no history of gestational diabetes mellitus (98%) or hypertensive disorders during pregnancy (96.4%) whereas anemia was common (25.0%). As for delivery complications and anemia, the prevalence was 12.3% and 25% respectively. The majority of infants (72.5%) were AGA births; 11.8% were SGA and 15.9% were LGA (Table 1). Mean head circumference was 33.20 ± 3.17 cm, and mean abdominal circumference was 27.44 ± 3.35 cm. Apgar scores averaged 8.31 ± 1.06 at 1 minute and 9.55 ± 1.07 at 5 minutes.

**Table 1:** Population characteristics of pregnant women in Lebanon (N = 618).

Maternal health characteristics^a^	Mean ± SD or %
Smoking T1	3.9 (%)
Smoking T2	1.9 (%)
Smoking T3	1.0 (%)
Pre-pregnancy BMI categories, kg/m²	
Underweight (<18.5)	3.9 (%)
Normal (18.5–24.9)	63.4 (%)
Overweight (25.0–29.9)	22.5 (%)
Obese (≥30.0)	10.2 (%)
Total GWG, kg	
Low	11.1 (kg)
Adequate	20.5 (kg)
Excessive	68.3 (kg)
GWG at T1, kg	1.17 ± 4.33 (kg)
Low	3.6 (kg)
Adequate	9.6 (kg)
Excessive	86.8 (kg)
GWG at T2, kg	5.34 ± 3.46 (kg)
Low	34.5 (kg)
Adequate	4.1 (kg)
Excessive	61.4 (kg)
GWG at T3, kg	7.09 ± 3.80 (kg)
Low	4.5 (kg)
Adequate	2.3 (kg)
Excessive	93.2 (kg)
Anemia	25 (%)
Delivery Complications	12.3 (%)
**Newborn characteristics**	
Infant sex, %	
Boy	58 (%)
Girl	42 (%)
Gestational age at delivery, weeks	37.6 ± 1.9 (weeks)
Mean birth weight, grams	3118. ± 598 (grams)
Birth weight, grams	
< 2500 g	11.0 (%)
2500–4500 g	85.6 (%)
≥ 4500 g	3.4 (%)
Birth weight z score, percentiles	
SGA (<10th percentile)	11.8 (%)
AGA (10th–90th percentiles)	72.3 (%)
LGA (>90th percentile)	15.9 (%)

^a^N = 618 Values are presented as means ± standard deviations unless otherwise specified. Percentages are shown for categorical variables.

Adherence to the LMeD was significantly different between trimester 1, 2 and 3. In trimester 3, a higher percentage of women (26.0%) had low adherence scores compared to trimester 1 and 2 (19.0% and 24.0% respectively) (p < 0.001), and in trimester 2 more women were identified in the high adherence group (30.0%) compared to trimester 1 (21.0%) and trimester 3 (25.0%) (S1 Table in S1 File).

### Trimestral comparisons for maternal factors among SGA, AGA and LGA infants (Table 2)

Table 2 compares maternal factors by trimester among SGA, AGA and LGA infants. Greater adherence to the LMeD was associated in both trimesters 2 (p < 0.033) and 3 (p < 0.038), but not in trimester 1, with birth of an AGA compared to a SGA infant. In contrast, GWG in T1 was associated with birth of an LGA infant (p < 0.011). With regards to blood pressure, higher MAP in T1 and T3 (p < 0.016 and p < 0.002, respectively) and higher PP (p < 0.002, p < 0.016, p < 0.001) in all 3 trimesters were associated with birth of an SGA infant. In contrast, lower PP across all trimesters were observed in mothers who gave birth to LGA infants. Most women were sedentary, and mothers who gave birth to LGA infants remained largely inactive in T2.

**Table 2:** Comparisons of maternal health factors across the SGA, AGA, and LGA groups across trimesters (N = 618).

Variables ^a^	SGA (N = 73)	AGA (N = 447)	LGA (N = 98)	p*
**Maternal Characteristics** ^**b**^				
Adherence to the LMeD T2	16.5 ± 4.2 ^A^	18.5 ± 3.9 ^B^	18.0 ± 4.9 ^AB^	0.033*
Adherence to the LMeD T3	16.5 ± 4.9 ^A^	18.8 ± 4.4 ^B^	18.2 ± 5.2 ^AB^	0.038*
Mean GWG T1, kg	1.29 ± 2.49 ^A^	1.55 ± 4.19 ^A^	3.02 ± 5.66 ^B^	0.011*
MAP T1, mm Hg	132.86 ± 12.86 ^A^	130.02 ± 12.67 ^B^	126.78 ± 14.42 ^B^	0.016*
MAP T3, mm Hg	140.31 ± 18.24 ^A^	134.06 ± 12.52 ^B^	135.67 ± 12.70 ^B^	0.002*
Pulse pressure T1, mm Hg	25.69 ± 18.17 ^A^	21.04 ± 18.28 ^B^	15.23 ± 19.08 ^B^	0.002*
Pulse pressure T2, mm Hg	25.15 ± 17.36 ^A^	20.50 ± 17.53 ^B^	16.44 ± 20.95 ^B^	0.016*
Pulse pressure T3, mm Hg	27.74 ± 20.57 ^A^	21.16 ± 18.86 ^B^	15.99 ± 19.02 ^B^	0.001*
Physical activity at T2				0.027*
Sedentary	80.3% ^A^	88.8% ^A^	94.4% ^B^	
Active	19.7%	11.2%	5.6%	

^a^N = 618 Values are presented as means ± standard deviations unless otherwise specified. Percentages are shown for categorical variables.

^b^Superscript letters (ᴬ, ᴮ, ᴬᴮ) indicate significant pairwise differences between groups based on Tukey’s post hoc test following one-way ANOVA**.**

*p < 0.05 indicates statistical significance.

With regards to psychosocial variables, poor sleep in T2, which was more prevalent among women who subsequently gave birth to LGA infants (86.5%, p < 0.038), was the only psychosocial variable to emerge as significantly related to birth weight. Interestingly, other psychological variables including stress and depression did not differ across birth weight categories, but their prevalence exceeded 50% of mothers in T1, T2 and T3. Further variables were analyzed and reported in S2 Table in S1 File. Gestational age, weight at T3, delivery complications, and previous macrosomia were significantly different among SGA, AGA and LGA (p < 0.05).

S3 Table in S1 File compares the dietary characteristics of mothers delivering SGA, AGA and LGA. Mothers of LGA infants had the highest consumption of eggs and olive oil, whereas mothers of SGA infants had the lowest consumption of dairy products and vegetables. Interestingly, dairy product consumption was highest among AGA infants (p < 0.05)

### Adjusted linear regression models for birth weight for gestational age (BWGA) and appropriate for gestational age (AGA)

Table 3 summarizes linear regression analyses of predictors for infant BWGA (n = 618) and determinants of AGA infants among 447 pregnant women. In Model 1-Maternal Characteristics revealed that parity, pre-pregnancy BMI, history of macrosomia, and total GWG significantly increased BWGA. Infant sex also played a role, with girls having lower BWGA. In Model 2- that included additional clinical variables such as GDM, IGT: FBG ≥ 5.6 mmol/L, MAP, PP, PSQI, and adherence to the LMeD uncovered in addition to parity, history of macrosomia, infant sex, total GWG, pre-pregnancy BMI, both PP and PSQI were also significant. However, adherence to the LMeD did not enter the model. In Model 3-Individual Food Groups, specific food groups emerged as significant determinants of BWGA revealing that higher intakes of olive oil, and eggs in the third trimester, dairy products in the first and second trimester, in addition to poorer quality sleep, were associated with increased BWGA. Infant sex continued to be negatively associated with BWGA, with girls having lower birth weights.

**Table 3:** Linear regression describing determinants of infant birth weight-for-gestational age (%) (N = 618) and AGA Infants (=447) in pregnant women in Lebanon.

Variable	Adjusted Model of BWGA^a^			Adjusted Model for AGA^b^		
	B	SE	p*	B	SE	p*
**Model 1- Maternal Characteristics**						
Parity, #	3.935	0.018	0.015	3.220	1.160	0.006
Infant sex, M = 0/ F = 1	−9.208	0.042	0.002	---	--	NS
Hx Macrosomia (No = 0/ Yes = 1)	19.686	7.032	0.001	--	--	NS
Total GWG, Kg	0.951	0.003	0.000	--	--	NS
Pre-pregnancy BMI, kg/m²	0.750	0.295	0.011	--	--	NS
**Model 2 – Maternal Health Factors**						NS
Parity, #	3.701	1.104	0.001	3.291	1.156	0.005
Infant sex, M = 0/ F = 1	−9.841	2.540	0.000	--	--	NS
Hx Macrosomia (No = 0/ Yes = 1)	19.736	7.014	0.005	--	--	NS
Total GWG, kg	0.960	0.225	0.000	--	--	NS
Pre-pregnancy BMI, kg/m²	0.713	0.291	0.015	--	--	NS
Pulse pressure T2, mm Hg	0.397	0.152	0.009	--	--	NS
Pulse pressure T3, mm Hg	−0.491	0.144	0.001	--	--	NS
PSQI T2	1.169	0.380	0.002	0.674	0.336	0.045
**Model 3 – Individual Food Groups**						
Parity, #	4.017	1.096	0.000	--	--	NS
Infant sex, M = 0/ F = 1	−8.990	2.525	0.000	--	--	NS
Hx Macrosomia (No = 0/ Yes = 1)	15.466	6.574	0.019	--	--	NS
Total GWG, kg	0.765	0.216	0.000	--	--	NS
PSQI T2	1.088	0.379	0.004	--	--	NS
Olive Oil T3, servings/day	2.898	1.279	0.024	--	--	NS
Parity, #	3.934	1.095	0.000	3.293	1.150	0.004
Infant sex, M = 0/ F = 1	−9.169	2.513	0.000	--	--	NS
Hx Macrosomia (No = 0/ Yes = 1)	16.186	6.518	0.013	--	--	NS
Total GWG, kg	3.934	1.095	0.000	--	--	NS
PSQI T2	1.171	0.379	0.002	--	--	NS
Dairy products T1, servings/day	−3.132	0.955	0.001	−1.892	0.690	0.006
Dairy products T2, servings/day	2.656	1.201	0.027	--	--	NS
Parity, #	3.738	1.102	0.001	--	--	NS
Infant sex, M = 0/ F = 1	−9.119	2.518	0.000	--	--	NS
Hx Macrosomia (No = 0/ Yes = 1)	13.814	6.621	0.037	--	--	NS
Total GWG, kg	0.733	0.216	0.001	--	--	NS
PSQI T2	1.131	0.378	0.003	--	--	NS
Eggs T3, servings/day	7.279	2.587	0.005	--	--	NS
Parity, #	--	--	--	3.726	1.168	0.002
Dried fruits T3, servings/day	--	--	--	6.324	2.564	0.014
Adherence to the LMeD T1	--	--	--	−3.807	1.843	0.040

^a^N = 618 for BWGA model and ^b^ N = 447 for AGA model.

^a^BWGA: In model 1, smoking T1, T2 and T3, maternal height, family history of diabetes did not enter the model. In model 2, GDM, FBG>=100 mg/dl in T1 and T3, MAP T1, T2, and T3, pulse pressure T1, PSQI T1 and T3, and adherence to the LMeD T1, T2 and T3 did not enter the model. In model 3, food groups that entered the model are olive oil T3, dairy products T1 and T2, and eggs T3.

^b^AGA: In model 1, smoking at T1, T2 and T3, infant sex, maternal height, family history of diabetes, history of macrosomia, total GWG, and pre-pregnancy BMI did not enter the model. In model 2, FBG in T1 and T3, GDM, MAP T1, T2 and T3, pulse pressure T1, T2 and T3, PSQI T1 and T3, and adherence to the LMeD T1, T2 and T3 did not enter the model. In model 3, food groups that entered the model are dried fruits T3 and dairy products T1.

*Only significant adjusted variables are shown.

“--” indicates non-significant associations.

NS indicates non-significant p-values.

In contrast, for the AGA infants, Model 1-Maternal Characteristics showed that only parity was associated with an AGA birth. Model 2-Maternal Health Factors confirmed that higher parity was associated with birth of an AGA infants. Model 3- Individual Food Groups which focused on individual foods revealed that adherence to the LMeD in T1 was inversely associated with AGA whereas higher intakes of dried fruits in T3 increased AGA birth weights, whereas higher dairy product intake lowered it in T1. Neither olive oil nor intake of eggs entered the models for birth of an AGA infant.

### Multiple logistic models for SGA with AGA, and LGA with AGA

SGA vs. AGA: Multiple logistic regression models were used to assess predictors of SGA compared with AGA infants (n = 520). Model 1-Maternal Characteristics, which included maternal characteristics, revealed that higher total GWG was associated with a decreased risk of SGA [OR=0.894, p < 0.001], whereas higher MAP in the third trimester increased the risk of SGA [OR=1.047, p < 0.004]. After adjustment, total GWG and MAP during the second and third trimesters remained significant predictors (Table 4).

**Table 4:** Multiple logistic regression describing the likelihood of SGA to AGA infants in pregnant women in Lebanon (N = 520).

	Unadjusted Model			Adjusted** Model		
SGA^a^	OR	CI	p*	OR	CI	p*
**Model 1 – Maternal characteristics**						
Total GWG, kg	0.894	0.329-0.781	0.001	0.511	0.337-0.774	0.002
MAP T2, mm Hg	--	--	--	0.958	0.928-0.990	0.000
MAP T3, mm Hg	1.047	1.015-1.078	0.004	1.045	1.017-1.073	0.001
Pulse pressure T3, mm Hg	1.039	1.002-1.076	0.037	--	--	NS
**Model 2 – Individual Food Groups**						
Total GWG, kg	0.430	0.267-0.695	0.001	0.483	0.315-0.740	0.001
MAP T2, mm Hg	0.948	0.909-0.988	0.012	0.956	0.925-0.987	0.007
MAP T3, mm Hg	1.047	1.012-1.084	0.009	1.049	1.020-1.079	0.001
Burghul T1, Serving/day	16.329	1.440-185.213	0.024	3.130	1.145-8.560	0.026

^a^N = 520

** In the adjusted model 1, parity, smoking T1, T2 and T3, pre-pregnancy BMI, infant sex, maternal height, family history of diabetes, FBG>=100 mg/dl, GDM, MAP T1, pulse pressure T1, T2 and T3, PSQI T1, T2 and T3, adherence to the LMeD T1, T2 and T3 did not enter the model. In adjusted model 2, the only food groups that entered are burghol in T1.

*Only significant adjusted variables are shown.

“--” indicates non-significant associations.

NS indicates non significant p-values.

Model 2-Individual Food Groups focused on specific food groups, identifying burghul (bulgur wheat) as a significant factor. Burghul consumption in the first trimester was associated with increased SGA risk [OR=16.329, p < 0.024]. This association persisted after adjusting for total GWG and MAP in T2 and T3.

LGA vs AGA: Multiple logistic regression models were used to assess predictors of LGA infants compared with AGA infants (n = 545) (Table 5). Model 1-Maternal Characteristics revealed that higher parity and previous macrosomia were associated with increased odds of LGA infants [OR=1.369, p < 0.010; OR=8.482, p < 0.001], whereas being a girl was associated with a lower likelihood of LGA [OR=0.340, p < 0.002]. Higher MAP in the third trimester was linked to increased LGA risk [OR=1.039, p < 0.016]. After adjustment using the stepwise forward model, parity, infant sex, and previous history of macrosomia remained significant, while family history of diabetes and PP in the first trimester were associated with a decreased risk of LGA [OR=0.456, p < 0.041; OR=0.977, p < 0.007].

**Table 5:** Multiple logistic regression describing the likelihood of LGA to AGA infants in pregnant women in Lebanon (N = 458).

	Unadjusted Model			Adjusted Model**		
LGA ^a^	OR	CI	p*	OR	CI	p*
**Model 1 – Maternal characteristics**						
Parity, #	1.369	1.077-1.740	0.010	1.452	1.165-1.811	0.001
Infant sex, M = 0/F = 1	0.340	0.173-0.670	0.002	0.314	0.166-0.594	0.000
Family Hx DM, No/Yes	--	--	--	0.456	0.215-0.968	0.041
Hx Macrosomia, No/Yes	8.482	2.533-28.407	0.001	6.447	2.145-19.376	0.001
MAP T3, mm Hg	1.039	1.007-1.072	0.016	--	--	NS
Pulse pressure T1, mm Hg	--	--	NS	0.977	0.961-0.994	0.007
PSQI T3	--	--	NS	1.081	1.000-1.167	0.049
**Model 2 – Individual Food Groups**						
Parity, #	1.367	1.074-1.740	0.011	1.498	1.208-1.859	0.000
Infant sex, M = 0/F = 1	0.327	0.165-0.650	0.001	0.352	0.187-0.663	0.001
Hx Macrosomia, No/Yes	8.321	2.433-28.460	0.001	6.145	2.231-16.928	0.000
Olive oil T2, Serving/day	--	--	NS	1.447	1.094-1.913	0.010

^a^N = 458.

*Only significant adjusted variables are shown.

** In the adjusted model 1, smoking T1, T2 and T3, maternal height, total GWG, pre-pregnancy BMI, FBG > 100 mg/dl, GDM, MAP T1, T2 and T3, pulse pressure T2 and T3, PSQI T1, T2 and adherence to the LMeD T1, T2, T3 did not enter the model. In adjusted model 2, the only food group that entered is olive oil.

“--” indicates non-significant associations.

NS indicates non-significant p-values.

Model 2-Individual Food Groups identified olive oil consumption as a significant predictor of LGA. A higher intake of olive oil was associated with an increased risk of LGA [OR=1.447, p < 0.010], along with significant associations with parity, infant sex, and previous history of macrosomia. (Table 5)

## Discussion

This national cohort study is the first in Lebanon to examine associations between adherence to the (LMeD, trimester-specific maternal psychosocial and health variables, and three birth weight categories: AGA, SGA, and LGA infants. Several key findings emerged. Adherence to the LMeD in the first trimester (T1) was associated with AGA births, whereas SGA and LGA were more strongly associated with specific dietary components and maternal health indicators. Appropriate GWG was associated with lower SGA risk, while higher burghul intake in T1 and elevated MAP in T2 and T3 were associated with higher SGA risk. LGA was associated with previous macrosomia, poor sleep quality in T3, and higher olive oil intake in T2, while higher PP in T1 was inversely associated with LGA.

### Adherence to LMeD and infant birth weight

Consistent with prior research [44,45], parity, infant sex, pre-pregnancy BMI, and total GWG were associated with birth weight-for-gestational age (BWGA). Maternal sleep quality, history of macrosomia, and vascular measures (PP and MAP) also showed associations. Although overall LMeD adherence was not associated with BWGA, trimester-specific effects were observed. Higher adherence in T1 was associated with lower birth weight within the AGA category, supporting evidence that early adherence to a Mediterranean dietary pattern may promote optimal fetal growth [46, 47]. Potential mechanisms include improved placental function, reduced vascular resistance, enhanced antioxidant status, and moderated insulin signaling [48,49]. Meta-analyses also link MeD adherence to improved maternal outcomes, including lower obesity, reduced gestational diabetes, improved blood pressure, and better sleep [2], which may contribute to favorable AGA outcomes.

### Specific food groups and birth weight outcomes

While overall LMeD adherence was associated with AGA births, individual food groups showed distinct associations across all birth weight categories. Higher olive oil intake was associated with higher BWGA in T3 and with LGA, consistent with evidence suggesting protective effects against SGA [37,38]. Dairy intake showed a negative association in T1 and a positive association in T2, aligning with studies linking milk consumption to increased birth weight, potentially via IGF-1 pathways [50,51]. Egg consumption in T3 was associated with higher BWGA, likely reflecting its high-quality protein, choline, and essential fatty acid content [52]. Dried fruit intake in T3 was associated with AGA births, possibly due to its nutrient density and antioxidant properties [53]. In contrast, higher burghul intake in T1 was associated with SGA, which may reflect limited dietary diversity or socioeconomic constraints [54]. These findings suggest that individual dietary components may influence fetal growth through specific nutrient-related mechanisms.

### Gestational weight gain, maternal BMI, and birth outcomes

Despite most women having normal or underweight pre-pregnancy BMI, a large proportion exceeded GWG recommendations, particularly among overweight and obese women, consistent with patterns observed in LMICs [55]. Total GWG was associated with higher BWGA and lower SGA risk, in agreement with findings from Lebanon [56] and global studies [57,58]. These results highlight the importance of monitoring GWG across pregnancy to support optimal fetal growth.

### Blood pressure and fetal growth

Hypertensive disorders of pregnancy affect approximately 10% of pregnancies globally [59]. In this cohort, few cases were identified using SBP and DBP thresholds, whereas elevated MAP in T2 and T3 was more common and associated with SGA risk, consistent with a recent meta-analysis [60]. PP was inversely associated with LGA in T1, potentially reflecting early vascular adaptation. These findings support the potential utility of MAP and PP as accessible indicators for identifying fetal growth risks, particularly in LMIC settings.

### Other determinants: parity, infant sex, macrosomia, and sleep

Female infants had lower birth weights, consistent with global evidence [61]. Higher parity was associated with AGA births [62], while previous macrosomia predicted higher birth weight recurrence [63,64]. Poor sleep quality in T3 was associated with increased LGA risk, aligning with studies linking sleep disturbances to altered glucose metabolism, insulin resistance, and elevated cortisol levels [65,66]. Although stress and depression were prevalent, they were not significantly associated with birth outcomes, possibly due to confounding factors such as the economic crisis and COVID-19 pandemic.

### Clinical implications

In the context of economic constraints and limited healthcare resources in Lebanon, these findings support the use of practical, low-cost strategies, including trimester-specific GWG monitoring, assessment of MAP and PP, and brief psychosocial screening, particularly for sleep. Early identification of women at risk of SGA or LGA may enable timely and feasible interventions.

### Strengths and limitations

Strengths include the longitudinal national cohort design, repeated trimester-specific measurements, and integration of dietary, anthropometric, and psychosocial data. Hierarchical modeling enabled identification of predictors of birth weight categories. However, variations in response rates may limit representativeness. Additional limitations include reliance on self-reported data, a highly educated sample (76%), and potential confounding from external factors such as economic instability and COVID-19. The LMeD tool has not been validated in pregnancy, and some food groups were not assessed, limiting comparability. Macronutrient composition, cooking methods, and total caloric intake were not analyzed. Variability in blood pressure measurement methods across clinics may also have introduced measurement error.

## Conclusion

These findings highlight the importance of a multifactorial approach to prenatal care, integrating diet, GWG, vascular monitoring, and sleep. Associations observed between LMeD adherence, maternal health indicators, and birth outcomes provide insight into factors influencing AGA, SGA, and LGA births. Further research is needed to examine long-term outcomes and contextual influences in Lebanon and the Eastern Mediterranean region.

## Supporting information

S1 File S1 Table. Dietary Characteristics of the Population and the Consumption of the Individual Food Groups of the Lebanese Mediterranean Diet (N = 618). S2 Table. Differences in Maternal and Infant Risk Factors across SGA, AGA and LGA Infants. S3 Table. Dietary Characteristics of Mothers Delivering SGA, AGA and LGA infants.
(ZIP)
