# Supplementary material for: Predictors of infant birth weights: Role of the Lebanese mediterranean diet, psychosocial factors and maternal health status
Source: PLoS One. 2026 Jun 10;21(6):e0351497. doi: 10.1371/journal.pone.0351497 (PMC13252803; doi:10.1371/journal.pone.0351497)
Supplement: S1 File — S2 Table. Differences in Maternal and Infant Risk Factors across SGA, AGA and LGA Infants. S3 Table. Dietary Characteristics of Mothers Delivering SGA, AGA and LGA infants. (ZIP) [file pone.0351497.s001.zip › Supporting information 3.docx]

**Supplemental Table 3.** Dietary Characteristics of Mothers Delivering SGA, AGA and LGA infants

**SGA AGA LGA**

**Food Groups (# of Servings/d) ^a,b^ Mean±SD Mean±SD Mean±SD p**

**Trimester 1**

Burghol, 1 cup 0.16±0.38 0.12±0.21 0.11±0.18 0.208

Dried Fruits, 1 ex 0.18±0.32 0.17±0.42 0.19±0.62 0.935

Legumes, 1 cup 0.27±0.41 0.24±0.27 0.28±0.44 0.392

Olive oil, 1 tsp 1.08±0.86 1.03±0.79 1.15±1.01 0.372

Eggs, 1 large 0.26±0.35^A^ 0.31±0.39^A^ 0.45±0.66^B^ 0.007*

Starchy Vegetables, 1 cup 0.37±0.36 0.42±0.51 0.35±0.38 0.338

Dairy Products, 1 serv 2.14±1.57 2.44±1.63 2.16±1.26 0.125

Fruits, 1 piece 2.30±1.57 2.47±1.90 2.43±1.71 0.749

Vegetables, 1 cup 1.77±1.18 1.76±1.15 1.96±1.28 0.323

**Trimester 2**

Burghol, 1 cup 0.13±0.37 0.14±0.20 0.13±0.19 0.947

Dried Fruits, 1 ex 0.18±0.32 0.17±0.42 0.19±0.62 0.935

Legumes, 1 cup 0.23±0.40 0.28±0.35 0.29±0.31 0.470

Olive Oil, 1 tsp 0.90±0.93^A^ 1.06±0.79^B^ 1.28±1.13A^B^  0.016*

Eggs, 1 large 0.20±0.33^A^ 0.35±0.47^B^ 0.48±0.64^C^ 0.001*

Starchy Vegetables, 1 cup 0.33±0.42 0.37±0.37 0.34±0.36 0.668

Dairy products, 1 serv 1.07±1.23^A^ 1.65±1.29^B^ 1.52±1.21^B^ 0.001*

Fruits, 1 piece 1.97±1.59 2.43±1.81 2.31±1.63 0.114

Vegetables, 1 cup 1.46±1.33^A^ 1.94±1.39^B^ 2.07±1.62^B^ 0.013*

**Trimester 3**

Burghol, 1 cup 0.14±0.39 0.13±0.19 0.11±0.18 0.714

^c^ Dried Fruits, 1 ex 0.15±0.27 0.19±0.43 0.22±0.62 0.579

Legumes, 1 cup 0.23±0.41 0.26±0.27 0.27±0.31 0.580

Olive Oil, 1 tsp 1.03±1.12^A^ 1.33±0.95^A^ 1.51±1.25^B^ 0.009*

Eggs, 1 large 0.17±0.34^A^ 0.35±0.43^B^ 0.54±0.70^C^ 0.000*

Starchy Vegetables, 1 cup 0.27±0.31 0.33±0.37 0.29±0.35 0.311

^c^ Dairy Products, 1 serv 1.00±1.33^A^ 1.62±1.34^B^ 1.40±1.21^B^ 0.001*

Fruits, 1 piece 1.98±1.70 2.20±1.52 1.92±1.37 0.182

Vegetables, 1 cup 1.28±1.25^A^ 1.69±1.18^B^ 1.71±1.35^B^ 0.025*

**_____________________________________________________________________________________**

^a^ Sample size= 618. Values are means ± standard deviation (SD) if normally distributed, median (min, max, interquartile range) if not normally distributed,

^b^ Food group intake was reported as the daily average number of servings consumed for each food group of the MeD. ^c^ 1 exchange (ex) for dried fruits (2tbsp raisins or cranberries, 2 pieces dates, 4 pieces apricots), and dairy products (1 cup milk or yogurt, 1 slice cheese or 2 tbsp labneh).

*Indicates significant associations using McNemar test for proportions followed by post hoc Tukey’s test.
