# Supplementary material for: Predictors of infant birth weights: Role of the Lebanese mediterranean diet, psychosocial factors and maternal health status
Source: PLoS One. 2026 Jun 10;21(6):e0351497. doi: 10.1371/journal.pone.0351497 (PMC13252803; doi:10.1371/journal.pone.0351497)
Supplement: S1 File — S2 Table. Differences in Maternal and Infant Risk Factors across SGA, AGA and LGA Infants. S3 Table. Dietary Characteristics of Mothers Delivering SGA, AGA and LGA infants. (ZIP) [file pone.0351497.s001.zip › Supporting information 1.docx]

**Supplemental table 1.** Dietary Characteristics of the Population and the Consumption of the Individual Food Groups of the Lebanese Mediterranean Diet (N=618)

_____________________________________________________________________________________

**Trimester 1 Trimester 2 Trimester 3**

**Maternal Dietary Intake Mean ± SD; % Mean ± SD; % Mean ± SD; % p**

**Mean Adherence Score** 17.47 ± 3.4 17.43 ± 4.04 17.5 ± 4.46

**Categories of Adherence**

Low (Score 9-15) 12.7 ±1.3 11.8 ± 1.9 11.5 ± 2.0 **<0.001***

19.0 24.0 26.0

Medium (Score 16-20) 17.3 ± 1.7 17.7 ± 1.7 17.7 ± 1.6

60.0 51.0 43.0

High (Score 21-27) 22.4 ± 1.4 22.4 ± 1.3 22.6 ± 1.5

21.0 25.0 30.0

_____________________________________________________________________________________

**^1^ Food Group Intake per Day, Serving Size**

Burghol, 1cup 0.12 ± 0.23 0.13 ± 0.22 0.13 ± 0.22 0.430

Starchy Vegetables, 1 cup 0.40 ± 0.48 0.36 ± 0.37 0.32 ± 0.36 0.256

Vegetables, 1 cup 1.79 ± 1.17 1.90 ± 1.43 1.64 ± 1.22 **0.044***

Fruits, 1 piece 2.44 ± 1.83 2.36 ± 1.76 2.13 ± 1.52 0.560

^2^ Dried Fruits, 1 serv 0.18 ± 0.45 0.18 ± 0.45 0.19 ± 0.45 0.875

^2^ Dairy products, 1 serv 2.36 ± 1.58 1.56 ± 1.28 1.51 ± 1.33 **0.035***

Olive oil, 1 tsp 1.05 ±.84 1.08 ± 0.87 1.32 ± 1.03 0.567

Eggs, 1 large 0.33 ± 0.44 0.36 ± 0.49 0.36 ± 0.48 0.789

Legumes, 1 cup 0.25 ± 0.32 0.28 ± 0.35 0.26 ± 0.30 0.567

Sample size= 618. Values are means ± standard deviation (SD) if normally distributed, median (min, max, interquartile range) if not normally distributed, or percentages (%) if binary. ^1^Food group intake was reported as the daily average number of servings consumed for each food group of the MeD.in each trimester: ^2^ 1 ex for dried fruits (2tbsp raisins or cranberries, 2 pieces dates, 4 pieces apricots), and dairy products (1 cup milk or yogurt, 1 slice cheese or 2 tbsp labneh). *Indicates significant associations using McNemar test for proportions. Abbreviations: IGT, impaired glucose tolerance, LMeD, Lebanese Mediterranean diet, tsp, teaspoon
