# Supplementary material for: Predictors of infant birth weights: Role of the Lebanese mediterranean diet, psychosocial factors and maternal health status
Source: PLoS One. 2026 Jun 10;21(6):e0351497. doi: 10.1371/journal.pone.0351497 (PMC13252803; doi:10.1371/journal.pone.0351497)
Supplement: S1 File — S2 Table. Differences in Maternal and Infant Risk Factors across SGA, AGA and LGA Infants. S3 Table. Dietary Characteristics of Mothers Delivering SGA, AGA and LGA infants. (ZIP) [file pone.0351497.s001.zip › Supporting information 2.docx]

**Supplemental Table 2.** Differences in Maternal and Infant Risk Factors across SGA, AGA and LGA Infants

**SGA (N=73) AGA (N=447) LGA (N=98)**

**Maternal Factors ^a^, ^b^ Mean±SD or % Mean±SD or % Mean±SD or % p**

| Maternal Age, years | 29.48± 5.94 | 29.23±4.93 | 28.97±4.92 | 0.804 |
| --- | --- | --- | --- | --- |
|  |  |  |  |  |
| Gestational age, weeks | 37.7±2.5 ^A^ | 37.8±1.6 ^A^ | 36.3±2.7 ^B^ | 0.000* |
| Maternal Height, meters | 1.63±0.06 | 1.63±0.06 | 1.64±0.07 | 0.305 |
| Weight T3, Kg | 75.65±11.62 ^A^ | 78.52±11.76 ^A^ | 82.57±13.86 ^B^ | 0.003* |
| Parity, number of gestations  0-1  2-4  >4 |  |  |  | 0.000* |
|  | 57.5 | 57.7 | 49.0 |  |
|  | 19.2 | 36.2 | 33.7 |  |
|  | 23.3 | 6.0 | 17.3 |  |
| Pre-pregnancy BMI, Kg/m^2^  Underweight (<18.5, %)  Normal (18.5-24.9, %)  Overweight (25-29.9, %)  Obese (≥30, %) | 5.5 | 4.0 | 2.0 | 0.095 |
|  | 60.3 | 62.2 | 71.4 |  |
|  | 27.4 | 23.9 | 12.2 |  |
|  | 6.8 | 9.8 | 14.3 |  |
| Delivery Complications  No, %  Yes, % |  |  |  |  |
|  | 71.4 | 87.4 | 84.9 | 0.011* |
|  | 28.6 | 12.6 | 15.1 |  |
| Maternal Delivery Type  Vaginal, %  Caesarian, % |  |  |  | 0.573 |
|  | 43.8 | 44.3 | 50.0 |  |
|  | 56.2 | 55.7 | 50.0 |  |
| Previous Caesarian  No, %  Yes, % |  |  |  | 0.167 |
|  | 81.0 | 69.3 | 68.0 |  |
|  | 19.0 | 30.7 | 32.0 |  |
| Previous Macrosomia  No, %  Yes, % | 95.1 ^A^ | 97.4 ^AB^ | 87.0 ^B^ | 0.000* |
|  | 4.9 | 2.6 | 13.0 |  |
| GDM  No, %  Yes, % |  |  |  | 0.058 |
|  | 90.3 | 95.8 | 90.7 |  |
|  | 9.7 | 4.2 | 9.3 |  |
| Mean Total GWG  Total GWG  Low, %  Adequate, %  Excessive, % | 11.98±4.97 | 14.17±5.71 | 16.21±7.13 | 0.001* |
|  | 34.5 ^A^ | 18.9 ^B^ | 14.6 ^B^ |  |
|  | 41.8 | 38.0 | 30.0 |  |
|  | 23.6 | 43.1 | 54.9 |  |
| Infant sex  Boy, %  Girl , % | 54.8 ^A^  45.2 | 55.5 ^B^  44.5 | 71.4 ^C^  28.6 | 0.013 |
| Infant Weight, grams | 2332.5±451.6 ^A^ | 3111.0±391.5 ^B^ | 3735.9±756.8 ^C^ | 0.000* |

| Adherence to the LMeD T1 | 16.6 ± 4.2 | 17.5 ± 3.9 | 18.0 ± 3.0 | 0.205 |
| --- | --- | --- | --- | --- |
| Adherence to the LMeD T2 | 16.5 ± 4.2 ^A^ | 18.5 ± 3.9 ^B^ | 18.0 ± 4.9 ^AB^ | 0.033* |
| Adherence to the LMeD T3 | 16.5 ± 4.9 ^A^ | 18.8 ± 4.4 ^B^ | 18.2 ± 5.2 ^AB^ | 0.038* |
| Mean GWG T1, kg | 1.29 ± 2.49 ^A^ | 1.55 ± 4.19 ^A^ | 3.02 ± 5.66 ^B^ | 0.011* |
| Mean GWG T2, kg | 4.29 ± 3.00 | 5.43 ± 3.62 | 5.52 ± 2.74 | 0.059 |
| Mean GWG T3, kg | 6.22 ± 3.69 | 7.10 ± 3.78 | 7.64 ± 3.85 | 0.100 |
| MAP T1, mm Hg | 132.86 ± 12.86 ^A^ | 130.02 ± 12.67 ^B^ | 126.78 ± 14.42 ^B^ | 0.016* |
| MAP T2, mm Hg | 133.25 ± 14.80 | 131.79 ± 12.08 | 129.05 ± 14.25 | 0.104 |
| MAP T3, mm Hg | 140.31 ± 18.24 ^A^ | 134.06 ± 12.52 ^B^ | 135.67 ± 12.70 ^B^ | 0.002* |
|  |  |  |  |  |
| Pulse pressure T1, mm Hg | 25.69 ± 18.17 ^A^ | 21.04 ± 18.28 ^B^ | 15.23 ± 19.08 ^B^ | 0.002* |
| Pulse pressure T2, mm Hg | 25.15 ± 17.36 ^A^ | 20.50 ± 17.53 ^B^ | 16.44 ± 20.95 ^B^ | 0.016* |
| Pulse pressure T3, mm Hg | 27.74 ± 20.57 ^A^ | 21.16 ± 18.86 ^B^ | 15.99 ± 19.02 ^B^ | 0.001* |
| Physical activity at T1 |  |  |  |  |
| Sedentary | 80.3 ^A^ | 84.0 ^AB^ | 87.6 ^B^ | 0.453 |
| Active | 19.4 | 16.0 | 12.4 |  |
| Physical activity at T2 |  |  |  | 0.027* |
| Sedentary | 80.3% ^A^ | 88.8% ^A^ | 94.4% ^B^ |  |
| Active | 19.7% | 11.2% | 5.6% |  |
| Physical activity at T3 |  |  |  | 0.131 |
| Sedentary | 90.2% | 94.6% | 97.7% |  |
| Active | 9.8% | 5.4% | 2.3% |  |
| FBG T1, mg/dL | 94.19 ± 11.04 | 92.39 ± 8.54 | 92.95 ± 8.57 | 0.335 |
| FBG T3, mg/dL | 95.59 ± 11.15 | 95.29 ± 10.67 | 96.44 ± 11.75 | 0.686 |
| IGT T1 (FBG ≥5.51 mmol/L.) |  |  |  | 0.776 |
| Yes | 12.3% | 10.3% | 12.2% |  |
| No | 87.7% | 89.7% | 87.8% |  |
| IGT T3 (FBG ≥5.51 mmol/L.) |  |  |  | 0.872 |
| Yes | 21.9% | 24.6% | 23.5% |  |
| No | 78.1% | 75.4% | 76.5% |  |
| **Psychosocial Variables** |  |  |  |  |
| Sleep score T1 |  |  |  | 0.323 |
| Bad Sleep (Score ≥5) | 57.5% | 50.6% | 57.1% |  |
| Good Sleep (Score <5) | 42.5% | 49.4% | 42.9% |  |
| Sleep score T2 |  |  |  | 0.038* |
| Bad Sleep (Score ≥5) | 75.0% ^A^ | 73.8% ^A^ | 86.5% ^B^ |  |
| Good Sleep (Score <5) | 25.0% | 26.2% | 13.5% |  |
| Sleep score T3 |  |  |  | 0.350 |
| Bad Sleep (Score ≥5) | 83.3% | 82.3% | 88.6% |  |
| Good Sleep (Score <5) | 16.7% | 17.7% | 11.4% |  |
| Mean Perceived Stress Score T1 | 18.61 ± 6.04 | 19.68 ± 5.92 | 20.55 ± 6.03 | 0.11 |
| Perceived Stress Score T1 |  |  |  | 0.160 |
| Low (Score ≤13) | 24.7% | 16.3% | 13.3% |  |
| Moderate (Score 14–26) | 68.5% | 73.2% | 71.4% |  |
| High (Score ≥27) | 6.8% | 10.5% | 15.3% |  |
| Mean Perceived Stress Score T2 | 20.26 ± 5.62 | 20.46 ± 6.45 | 22.17 ± 6.57 | 0.060 |
| Perceived Stress Score T2 |  |  |  | 0.125 |
| Low (Score ≤13) | 14.8% | 16.7% | 11.2% |  |
| Moderate (Score 14–26) | 75.4% | 66.9% | 64.0% |  |
| High (Score ≥27) | 9.8% | 16.4% | 24.7% |  |
| Mean Perceived Stress Score T3 | 20.13 ± 6.29 | 21.43 ± 6.98 | 21.92 ± 7.14 | 0.283 |
| Perceived Stress Score T3 |  |  |  | 0.343 |
| Low (Score ≤13) | 19.7% | 15.1% | 12.5% |  |
| Moderate (Score 14–26) | 65.6% | 62.6% | 59.1% |  |
| High (Score ≥27) | 14.8% | 22.3% | 28.4% |  |
| Mean Edinburgh Depression Scale T1 | 9.15 ± 5.00 | 9.73 ± 4.91 | 9.18 ± 5.49 | 0.465 |
| Edinburgh Depression Scale T1 |  |  |  | 0.809 |
| Non-Depressed (Score <10) | 52.1% | 51.0% | 54.6% |  |
| Depressed (Score ≥10) | 47.9% | 49.0% | 45.4% |  |
| Mean Edinburgh Depression Scale T2 | 10.38 ± 4.34 | 9.67 ± 4.80 | 10.00 ± 5.60 | 0.523 |
| Edinburgh Depression Scale T2 |  |  |  | 0.149 |
| Non-Depressed (Score <10) | 31.7% | 44.7% | 45.5% |  |
| Depressed (Score ≥10) | 68.3% | 55.3% | 54.5% |  |
| Mean Edinburgh Depression Scale T3 | 10.65 ± 5.09 | 9.41 ± 4.94 | 9.63 ± 5.88 | 0.216 |
| Edinburgh Depression Scale T3 |  |  |  | 0.326 |
| Non-Depressed (Score <10) | 38.3% | 48.6% | 48.3% |  |
| Depressed (Score ≥10) | 61.7% | 51.4% | 51.7% |  |

^a^ Values are means ± standard deviation (SD) if normally distributed, median (min-max) if not normally distributed, or percentages (%) if binary, unless otherwise specified.

^b^ Superscript letters (ᴬ, ᴮ, ᴬᴮ) indicate significant pairwise differences between groups based on **Tukey's post hoc test following one-way ANOVA.**
*Indicates significant associations.
