# Supplementary material for: Predictors of infant birth weights: Role of the Lebanese mediterranean diet, psychosocial factors and maternal health status
Source: PLoS One. 2026 Jun 10;21(6):e0351497. doi: 10.1371/journal.pone.0351497 (PMC13252803; doi:10.1371/journal.pone.0351497)
Supplement: Attachment — Submitted filename: Summary of Comments on PONE-D-25-31196_SB_12.7.pdf [file pone.0351497.s002.pdf]

PLOS ONE

Predictors of Infant Birth Weights: Role of the Lebanese Mediterranean Diet, Psychosocial Factors and Maternal Health Status

--Manuscript Draft--

**Manuscript Number:**	PONE-D-25-31196
**Article Type:**	Research Article
**Full Title:**	Predictors of Infant Birth Weights: Role of the Lebanese Mediterranean Diet, Psychosocial Factors and Maternal Health Status
**Short Title:**	Predictors of Infant Birth Weights among Lebanese pregnant women
**Corresponding Author:**	Jessy El Hayek Fares, PhD<br>Notre Dame University Louaize<br>Zouk Mikael, LEBANON
**Keywords:**	LMeD, Lebanese Mediterranean diet, SGA, small for gestational age, LGA, large for gestational age, AGA, appropriate for gestational age
**Abstract:**	Background: The Mediterranean diet (MeD) is associated with favorable pregnancy outcomes, but contribution of this dietary pattern during pregnancy with small (SGA), appropriate (AGA) and large (LGA) for gestational age births has not been investigated.<br>Methods: For this prospective national cohort study 618 Lebanese pregnant women were recruited. Infant birth weight was categorized into SGA (n=73), AGA (n=447) and LGA (n=98). Modifiers of birth weight outcomes included dietary adherence to Lebanese MeD (LMeD), trimestral and total weight gain, MAP (mean arterial pressure) and PP (pulse pressure) and psychosocial, sociodemographic, and maternal health factors. Descriptive statistics compared differences among SGA, AGA, LGA infants. Hierarchical linear regression modeling identified determinants for birth weight categories and hierarchical logistic regression modeling was used to identify factors associated with increasing the likelihood of SGA or LGA compared to AGA births.<br>Results: Adherence to the LMeD was associated with AGA birth weights where intakes of dairy products were associated with lower normal AGA births and dried fruits with higher normal AGA births. Adherence to LMeD did not enter models for SGA or LGA but intakes of specific foods and maternal health status indicators did. For SGA infants, appropriate GWG mitigated against a low birth weight whereas higher burghul intake in T1 and higher MAP in T2 and T3 were linked to increased SGA risk. For LGA infants, greater parity, previous macrosomia and poor sleep quality in T3, and higher intake of olive oil in T2 were associated with higher risk of a LGA birth whereas higher pulse pressure in T1 decreased the odds of a LGA birth.<br>Conclusions: Evidence suggests that screening for specific risks is important to improve birth weight outcomes. These include screening for family history of diabetes and macrosomia, targeting trimestral weight gain recommendations, monitoring mean arterial and pulse pressures and sleep quality and promoting adherence to the LMeD.
**Order of Authors:**	Paula Hage Boutros, PhD
	Jessy El Hayek Fares
	Maya Bassil
	Kristine G. Koski
**Additional Information:**	
**Question**	**Response**
**Financial Disclosure**<br><br>Enter a financial disclosure statement that describes the sources of funding for the work included in this submission. Review the submission guidelines for detailed	The author(s) received no specific funding for this work.

Powered by Editorial Manager® and ProduXion Manager® from Aries Systems Corporation

Summary of Comments on PONE-D-25-31196_SB_12.7.pdf

Page: 1

Author: Shrish Budree Subject: Comment on Text Date: 12/9/2025 9:19:18 AM

The authors are commended for conducting such a robust study in a population where the is a paucity of data related to pregnancy outcomes.

However, there are several concerns about some of the unusual finding from this study such as associations of healthy fat intake (olive oil) and wheat with poor pregnancy outcomes and (LGA and SGA respectively), high MAP during pregnancy being associated with LGA and poor sleep being associated with LGA. The authors are encouraged to robustly explain these findings and how they contradict current understandings in this field.

requirements. View published research articles from *PLOS ONE* for specific examples.<br><br>This statement is required for submission and **will appear in the published article** if the submission is accepted. Please make sure it is accurate.<br><br>**Funded studies**<br>Enter a statement with the following details:<br>• Initials of the authors who received each award<br>• Grant numbers awarded to each author<br>• The full name of each funder<br>• URL of each funder website<br>• Did the sponsors or funders play any role in the study design, data collection and analysis, decision to publish, or preparation of the manuscript?<br><br>Did you receive funding for this work?	
**Competing Interests**<br><br>Use the instructions below to enter a competing interest statement for this submission. On behalf of all authors, disclose any competing interests that could be perceived to bias this work—acknowledging all financial support and any other relevant financial or non-financial competing interests.<br><br>This statement is required for submission and **will appear in the published article** if the submission is accepted. Please make sure it is accurate and that any funding sources listed in your Funding Information later in the submission form are also declared in your Financial Disclosure statement.<br><br>View published research articles from *PLOS ONE* for specific examples.	The authors have declared that no competing interests exist.

This page contains no comments

**NO authors have competing interests**<br><br>Enter: *The authors have declared that no competing interests exist.*<br><br>**Authors with competing interests**<br><br>Enter competing interest details beginning with this statement:<br><br>*I have read the journal's policy and the authors of this manuscript have the following competing interests: [insert competing interests here]*<br><br>* typeset	
**Ethics Statement**<br><br>Enter an ethics statement for this submission. This statement is required if the study involved:<br><br>• Human participants<br>• Human specimens or tissue<br>• Vertebrate animals or cephalopods<br>• Vertebrate embryos or tissues<br>• Field research<br><br>Write "N/A" if the submission does not require an ethics statement.<br><br>General guidance is provided below. Consult the submission guidelines for detailed instructions. **Make sure that all information entered here is included in the Methods section of the manuscript.**	This study was conducted in accordance with the ethical principles of the Declaration of Helsinki. Ethical approval was obtained from the Bellevue Medical Center Institutional Review Board (IRB). In addition, written permission was secured from the supervising physician or head of each participating private clinic, allowing the research team to conduct the survey and access relevant medical records. Recruitment took place between December 15, 2019, and June 30, 2021. Written informed consent was obtained from all participants, who were assured that participation was voluntary and that they could withdraw at any time without any consequences to their medical care.

This page contains no comments

**Format for specific study types**<br><br>**Human Subject Research (involving human participants and/or tissue)**<br>• Give the name of the institutional review board or ethics committee that approved the study<br>• Include the approval number and/or a statement indicating approval of this research<br>• Indicate the form of consent obtained (written/oral) or the reason that consent was not obtained (e.g. the data were analyzed anonymously)<br><br>**Animal Research (involving vertebrate animals, embryos or tissues)**<br>• Provide the name of the Institutional Animal Care and Use Committee (IACUC) or other relevant ethics board that reviewed the study protocol, and indicate whether they approved this research or granted a formal waiver of ethical approval<br>• Include an approval number if one was obtained<br>• If the study involved *non-human primates*, add *additional details* about animal welfare and steps taken to ameliorate suffering<br>• If anesthesia, euthanasia, or any kind of animal sacrifice is part of the study, include briefly which substances and/or methods were applied<br><br>**Field Research**<br><br>Include the following details if this study involves the collection of plant, animal, or other materials from a natural setting:<br>• Field permit number<br>• Name of the institution or relevant body that granted permission	
**Data Availability**<br><br>Authors are required to make all data underlying the findings described fully available, without restriction, and from the time of publication. PLOS allows rare exceptions to address legal and ethical concerns. See the PLOS Data Policy and FAQ for detailed information.	Yes - all data are fully available without restriction

This page contains no comments

A Data Availability Statement describing where the data can be found is required at submission. Your answers to this question constitute the Data Availability Statement and **will be published in the article**, if accepted.<br><br>**Important:** Stating 'data available on request from the author' is not sufficient. If your data are only available upon request, select 'No' for the first question and explain your exceptional situation in the text box.<br><br>Do the authors confirm that all data underlying the findings described in their manuscript are fully available without restriction?	
**Describe where the data may be found in full sentences. If you are copying our sample text, replace any instances of XXX with the appropriate details.**<br><br>• If the data are **held or will be held in a public repository**, include URLs, accession numbers or DOIs. If this information will only be available after acceptance, indicate this by ticking the box below. For example: *All XXX files are available from the XXX database (accession number(s) XXX, XXX.).*<br>• If the data are all contained **within the manuscript and/or Supporting Information files**, enter the following: *All relevant data are within the manuscript and its Supporting Information files.*<br>• If neither of these applies but you are able to provide **details of access elsewhere**, with or without limitations, please do so. For example:<br><br>*Data cannot be shared publicly because of [XXX]. Data are available from the XXX Institutional Data Access / Ethics Committee (contact via XXX) for researchers who meet the criteria for access to confidential data.*<br><br>*The data underlying the results presented in the study are available from (include the name of the third party*	All relevant data are within the manuscript and its Supporting Information files

This page contains no comments

and contact information or URL).<br>• This text is appropriate if the data are owned by a third party and authors do not have permission to share the data.<br><br>* typeset	
Additional data availability information:	Tick here if the URLs/accession numbers/DOIs will be available only after acceptance of the manuscript for publication so that we can ensure their inclusion before publication.

Powered by Editorial Manager® and ProduXion Manager® from Aries Systems Corporation

This page contains no comments

Manuscript Click here to access/download;Manuscript;Manuscript PLOS One.docx

Predictors of infant birth weights: Role of the Lebanese Mediterranean diet, psychosocial factors and maternal health status

Paula H. Boutros ¶1, Jessy E. Hayek ¶2*, Maya Bassil[3], Kris G. Koski ¶4.

[1] Department of Nutrition, Faculty of Health Sciences, Modern University for Business and Science, Ramiz Sarkis, Beirut, Beirut 1137501, Lebann

[2] Faculty of Nursing and Health Sciences, Notre Dame University- Louaize (NDU), Zouk Mosbeh, Lebanon PO Box:72 Zouk Mikael

[3] Department of Nutrition Sciences, College of Health Sciences, QU Health, Qatar University, Doha, Qatar

[4] School of Human Nutrition McGill University - Macdonald Campus 21111 Lakeshore Road, Ste Anne de Bellevue Quebec H9X 3V9, Canada

*Corresponding Author
Email: jelhayek@ndu.edu.lb (JH)

¶These authors contributed equally to this work.

This page contains no comments

Abstract

Background: The Mediterranean diet (MeD) is associated with favorable pregnancy outcomes, but contribution of this dietary pattern during pregnancy with small (SGA), appropriate (AGA) and large (LGA) for gestational age births is limited.

Methods: For this prospective national cohort study 618 Lebanese pregnant women were recruited. Infant birth weight was categorized into SGA (n=73), AGA (n=447) and LGA (n=98). Modifiers of birth weight outcomes included dietary adherence to Lebanese MeD (LMeD), trimestral and total weight gain, MAP (mean arterial pressure) and PP (pulse pressure) and psychosocial, sociodemographic, and maternal health factors. Descriptive statistics compared differences among SGA, AGA, and LGA infants. Hierarchical linear regression modeling identified determinants for birth weight categories and hierarchical logistic regression modeling was used to identify factors associated with increasing the likelihood of SGA or LGA compared to AGA births.

Results: Adherence to the LMeD was associated with AGA birth weights where intakes of dairy products were associated with lower normal AGA births and dried fruits with higher normal AGA births. Adherence to LMeD did not enter models for SGA or LGA but intakes of specific foods and maternal health status indicators did. For SGA infants, appropriate GWG mitigated against a low birth weight whereas higher burghul intake in T1 and higher MAP in T2 and T3 were linked to increased SGA risk For LGA infants, greater parity, previous macrosomia and poor sleep quality in T3, and higher intake of olive oil in T2 were associated with higher risk of a LGA birth whereas higher PP in T1 decreased the odds of a LGA birth.

This page contains no comments

Conclusions: Screening for family history of diabetes and macrosomia, targeting trimestral weight gain recommendations, monitoring MAP, PP and sleep quality and promoting adherence to the LMeD.

This page contains no comments

Introduction

Eastern Mediterranean countries (EMCs) are currently undergoing a nutrition transition, with economic and population growth leading to a shift from the Mediterranean diet (MeD) to the adoption of Western dietary patterns and sedentary lifestyles [1]. This nutritional transition has significant implications for women of childbearing age, as their nutritional choices before and during pregnancy play crucial roles in determining infant birth weight [2, 3]. Understanding these dietary changes is essential for promoting healthier outcomes in this population. While existing research has highlighted various factors influencing infant birth weight, including maternal diet, weight and gestational weight gain (GWG), and psychosocial factors, a deeper exploration of their interplay is necessary to fully understand their individual contributions [4].

Adherence to the MeD, which emphasizes the consumption of fruits, vegetables, whole grains, and healthy fats, has been associated with improved pregnancy outcomes [5]. Several studies have indicated that better adherence to this dietary pattern correlates with favorable infant birth weights [5], lower odds of poor intrauterine growth, and reduced risks of SGA and LGA [6-11]. Furthermore, high intake of fruits, vegetables, and dairy products is associated with higher infant growth [12, 13], as well as adherence to a MeD supplemented with extra virgin olive oil [14]. Conversely, poor adherence to the MeD, especially in early pregnancy, has been shown to reduce placental growth and decrease infant birth weight [15]. Despite this, most research has focused on either early or late pregnancy, with few studies assessing continuous adherence to MeD throughout targeted timepoints during gestation [16].

This page contains no comments

Excessive weight gain during pregnancy is another critical factor influencing infant birth weight. Studies have shown that higher GWG is linked to increased birth weight and macrosomia [17], with implications for long-term health outcomes for both mothers and children [18]. In contrast, inadequate weight gain during pregnancy can lead to lower birth weights, which poses risks for neonatal complications [19]. Pregnancy-induced insulin resistance, which becomes more pronounced in overweight and obese women, further increases the risk of SGA infants [20, 21]. Moreover, hypertensive disorders of pregnancy (HDPs) are established causes of uteroplacental insufficiency, reducing nutrient and oxygen transfer to the fetus and leading to restricted growth [22, 23, 24, 25, 26, 27]. In low and middle income countries (LMICs), undiagnosed or unmanaged hypertension remains a significant contributor to SGA births, particularly among women with fewer than four antenatal visits [28]. Additionally, gestational diabetes mellitus (GDM) is associated with low birth weight and neonatal complications [29, 30].

Psychological factors such as stress, depression, and sleep disorders also play a significant role in gestational outcomes. Poor maternal sleep quality and sleep disorders during pregnancy have been linked to adverse infant outcomes, including low birth weight, impaired neurodevelopment, and increased risk of preterm birth. Disrupted maternal sleep may also affect fetal growth and development through altered maternal stress responses and inflammation, potentially impacting the infant's long-term health and well-being [31]. For example, maternal stress can lead to lower fetal weight gain from the second trimester onward [32], and both stress and depression before 20 weeks of pregnancy increase the likelihood of SGA infants being born [2].

Page: 11

Author: Shrish Budree Date: 12/2/2025 9:18:12 PM

Provide more details on these long term outcomes. Clarify whether these are negative outcomes

Author: Shrish Budree Date: 12/2/2025 9:25:19 PM

Clarify whether this is referring to gestational diabetes which is a pathological state of insulin resistance

Author: Shrish Budree Date: 12/2/2025 9:31:00 PM

should include pre-eclampsia

Author: Shrish Budree Date: 12/2/2025 9:33:58 PM

intimate partner violence

Author: Shrish Budree Date: 12/2/2025 9:36:11 PM

reference

Understanding these predictors is crucial for modulating infant birth weight, especially in the Mediterranean region, where studies are limited. Our study aims to 1) identify predictors of AGA, SGA and LGA infants including psychosocial factors such as stress, sleep and depression as well as pre-pregnancy BMI and GWG; 2) evaluate the impact of adherence to the Lebanese Mediterranean Diet (LMeD) on AGA, SGA and LGA infants; and 3) assess the role of specific LMeD food items on these outcomes.

Materials and methods

Study participants

This national prospective cohort study recruited pregnant women from the 6 different governorates of Lebanon that included Mount Lebanon (30.7%), Beirut (12.4%), Bekaa (16.4%) South Lebanon (and Nabatieh) (29.4%), and Akkar (3.7 %.) The inclusion criterion was pregnant Lebanese women > 18 years with a singleton pregnancy. The exclusion criteria were women 1) with multiple gestations, 2) prepregnancy diabetes, and 3) those carrying a fetus with structural malformation, chromosomal anomalies or TORCH (toxoplasmosis, rubella, cytomegalovirus, herpes and other agent) infections.

We used Epiinfo software to calculate the sample size needed via the Fleiss formula with correction [33]. The confidence interval was set at 95%, the power at 80% and the ratio (unexposed/exposed to the MeD=1). A sample of 618 participants was targeted after adjusting

Author: Shrish Budree Date: 12/2/2025 9:46:14 PM

Were participants recruited during any trimester of pregnancy?

Author: Shrish Budree Date: 12/2/2025 9:53:13 PM

What is the outcome measure that this study is powered on? What are the event rates in exposed/ unexposed and what is the alpha?

for a 20% loss to follow-up. Simple random sampling among all obstetric clinics in Lebanon was performed with SPSS version 22.0 (Statistical Package for Social Sciences yr). Among the 732 obstetric clinics, a total of 20 private and hospital-based private clinics were selected: Mount Lebanon (n=3), Beirut (n=5), Bekaa (n=4), South China (n=3), North China (n=4), and Akkar (n=1), with a minimum of 30 women recruited from each clinic.

This study was conducted in accordance with the ethical principles of the Declaration of Helsinki. Ethical approval was obtained from the Bellevue Medical Center Institutional Review Board (IRB). In addition, written permission was secured from the supervising physician or head of each participating private clinic, allowing the research team to conduct the survey and access relevant medical records. Recruitment took place between December 15, 2019, and June 30, 2021. Healthy pregnant women (<15 weeks gestation) attending routine obstetric visits were approached by trained research staff, provided with detailed information about the study, and invited to participate. Written informed consent was obtained from all participants, who were assured that participation was voluntary and that they could withdraw at any time without any consequences to their medical care.

Study Design

Pregnant women were followed from the 1st trimester (<15 weeks of gestation) until delivery, with follow-up interviews in the 2nd (24--28 weeks) and 3rd trimesters (34--37 weeks). The initial interview was conducted in person via a sociodemographic questionnaire and 5

This page contains no comments

validated questionnaires: 1) the Perceived Stress Scale (PSS10) [34], 2) the Pittsburgh Sleep Quality Index (PSQI) [35], 3) the Edinsburgh Perinatal/Postnatal Depression Scale (EPDS) [36], 4) the FFQ and LMeD score [37], and 5) the Canadian Physical Activity Readiness Medical Examination (PARmed-X for pregnancy) [38], where we categorized women as sedentary or active. The questionnaires were readministered in the 2nd and 3rd trimesters of pregnancy via phone interviews.

Clinical data, including self-reported prepregnancy weight, vitamin/mineral over the counter supplement intakes, presence of anemia and medical history, were obtained from medical chart reviews. At follow-up visits during each trimester, additional clinical indicators, including weight gain per trimester, blood pressure measurements [SBP, DBP, MAP. PP] and general maternal health status, were collected from medical charts. MAP and PP were calculated using SBP and DBP. Infant data were collected upon delivery.

Dietary assessment and adherence to the Lebanese Mediterranean diet

Data on food intake were collected via a previously validated 61-item FFQ specifically for the Lebanese diet by Naja et al., 2015 [37], which is representative of the LMeD and incorporates traditional Lebanese dishes. The FFQ consists of 9 food categories, including 1) breads and cereals, 2) dairy products, 3) fruits and juices, 4) vegetables, 5) meat and alternates, 6) fats and oils, (7) sweets and desserts, 8) beverages, and 9) miscellaneous. The LMeD was developed to assess adherence to a Lebanese version of the MeD. The authors were able to identify an LMeD pattern from the FFQ with the following 9 food groups because of their consistently high loading on this pattern: fruits, vegetables, legumes, olive oil, burghul (crushed

Page: 14

Author: Shrish Budree Date: 12/3/2025 9:20:33 PM

Clarify whether this was abstracted from medical charts or performed by study staff

Author: Shrish Budree Date: 12/6/2025 9:20:47 AM

This is not clear - consider rewording. Provide an explanation of why authors further subset FFQ data into food groups. Explain the terms 'LMed pattern' and 'high loading on pattern'.

whole wheat), milk and dairy products, starchy vegetables (potato, corn and beans), dried fruits and eggs. The LMeD index used in this study shares common food groups with other indices developed in Italy [39], Spain [40], Greece [41], France [42], and 9 Mediterranean European countries [43]; however, in the LMeD, other main components, such as fish, red meat, poultry, and wine consumption, are missing [37].

Adherence to the LMeD was measured on the basis of the intake of servings of each of the 9 food groups, with 1, 2, and 3 points assigned to consumption in the 1st, 2nd, and 3rd tertiles, respectively. Scores ranged between 9 and 27, with 9 reflecting the least adherence and 27 reflecting the highest adherence to the LMeD. This tool had a good correlation (r=0.56) with the Italian MeD tool [35]. Alcohol intake was excluded because consumption is virtually absent in this study population.

Clinical measurements

Pre-pregnancy BMI

Pre-pregnancy BMI data were collected at baseline from obstetrical charts and were used to classify women as underweight (<18.5 kg/m²), normal (18.5–24.9 kg/m²), overweight (25–29.9 kg/m²), or obese (≥30 kg/m²) [44].

Total GWG and per trimester GWG

Page: 15

Author: Shrish Budree Date: 12/3/2025 9:28:04 PM

Did the authors consider tobacco exposure (smoking) which is a well described cause of IUGR

The total GWG in kg was calculated by subtracting total weight gain from prepregnancy weight, and GWG was classified as low, adequate, or excessive according to the prepregnancy BMI according to the recommendations of the Institute of Medicine (IOM) (underweight 12.5–18 kg, normal weight 11.5–16 kg, overweight 7–11.5 kg, and obese 5–9 kg) [45]. GWG per trimester was classified as adequate, excessive, or low according to the following recommendations: For the 1st trimester, GWG above 2 kg or below 1 kg was considered excessive or low, respectively, for all categories of BMI. For the 2nd and 3rd trimesters, the recommended weight gain per week for each BMI category is as follows: 0.45–0.6 kg/wk for underweight women, 0.35–0.45 kg/wk for normal weight women, and 0.2–0.3 kg/wk for both overweight and obese women. Weight gain below or above these values was classified as low or excessive, respectively [46].

Fasting blood glucose (FBG), impaired glucose tolerance, and diagnosis of GDM

FBG values were collected from obstetric charts in trimesters 1 and 3 only and were categorized into normal FBG values <5.6 mmol/ and impaired glucose ≥5.6 mmol/L during the second trimester visit. GDM diagnosis (yes/no) was confirmed. Obstetricians in Lebanon followed the two-step 50 g oral glucose tolerance test between 28–32 weeks in line with the American College of Obstetricians and Gynecologists for the diagnosis of GDM [47].

Mean arterial pressure (MAP) and pulse pressure (PP)

This page contains no comments

Trimester-specific cutoffs for elevated MAP (eMAP) during pregnancy were defined as >87 mmHg (10–<18 weeks), >84 mmHg (18–34 weeks), and >86 mmHg (after 34 weeks) [48]; low MAP was defined as <70 mmHg [49]. MAP is calculated as MAP=DBP+ 1/3 (SBP−DBP). PP was calculated as follows: PP = SBP - DBP. A prior large population study on normal pregnant women was used to define high PP (>68 mmHg) and low PP (<42 mmHg) [49].

Infant birth outcomes

Infants with birth weights <10th percentile and >90th percentile according to their gestational age were classified as SGA and LGA, respectively, whereas infants with birth weights between the 10th and 90th percentiles were classified as AGA using the National Center for Health Statistics from 2011 [50].

Psychosocial variables

Perceived Stress Scale (PSS10): Perceived stress was evaluated using the Perceived Stress Scale (PSS10), which was later shortened to 10 items [34]. It has been validated in a Lebanese pregnant population with test-retest reliability and Cronbach's alpha=0.74; Correlation r=0.48 and r=0.58 with a general health questionnaire and EPDS [34]. A global sum score of 0-40 is obtained with higher scores indicating higher perceived stress. The categories are: Low stress (score 0-13), moderate stress (score 14-26), and high stress (score 27-40).

Author: Shrish Budree Date: 12/3/2025 9:49:09 PM

How was birth weight collected? How was GA determined during pregnancy ie. by ultrasound, last menstrual period?

Author: Shrish Budree Date: 12/6/2025 9:39:04 AM

Can the authors provide a rational for using the NCHS2011 reference standards which focuses on a U.S. population as opposed to the Intergrowth21 standards that represent an international population.

Pittsburgh Sleep Quality Index (PSQI): PSQI is a self-reported tool that assesses sleep quality over a 1-month period. A global score ranges between 0-21. A score of equal or greater to 5 indicates poor sleep, while a score of less than 5 indicates good sleep. It has been validated in an Arabic population with Cronbach's alpha=0.74, correlation r=0.36 and r=0.19, with the Insomnia Severity Index and Medical Outcome Study Short Form [35].

Edinburgh Perinatal/Postnatal Depression Scale (EPDS): Edinburgh Perinatal/Postnatal Depression Scale (EPDS) is a commonly used 10-item scale in clinical practice to identify women at risk of perinatal depression. A global sum of 0-30 is obtained. A score of 10 or greater indicates possible depression. It has been validated in a Lebanese pregnant population with Cronbach's alpha=0.84, correlation r=0.57 with the Present State Examination [36].

Statistical analyses

Statistical analyses were conducted via SPSS version 25. Descriptive statistics included frequencies and percentages for qualitative data and means, standard deviations, medians, minimums, and maximums for quantitative data, with visual representations via histograms. The dependent variable was infant birth weight, which was categorized into SGA, AGA and LGA using the INTERGROWTH_EFW calculator [51].

Author: Shrish Budree Date: 12/6/2025 9:44:30 AM

Can authors clarify which gestational age reference standard was used to categorize AGA, SGA/LGA. NCHS standards mentioned above. For linear models, can authors clarify whether percentile for BWGA were the dependent variable.

Adherence to the LMeD, maternal characteristics and psychosocial variables were compared across AGA, SGA, and LGA birth weight categories by trimesters. Maternal characteristics included pre-pregnancy BMI, smoking, physical activity status (sedentary vs active), GWG, fasting blood glucose (FBG) and evidence of impaired glucose tolerance (IGT), blood pressure measurements (SBP, DBP, MAP, PP), and assessment of stress, sleep, depression. Bivariate analyses were performed using chi-square tests and ANOVA, with significance set at $p < 0.05$. Significant differences were further explored using post hoc Tukey tests.

Linear regression models were used to assess factors associated with infant birth weight for gestational age (BWGA) for the study population (n=618) and for the subset of AGA infants (n=447) using hierarchical modeling. For Model 1 - maternal characteristics for BWGA- were adjusted for smoking at T1, T2, T3, parity, infant sex, maternal height, pre-pregnancy BMI, family history of diabetes, history of macrosomia, and total GWG. Model 2- Maternal Health Factors - included significant variables from model 1 and further adjusted for GDM, impaired fasting blood glucose≥5.6 mmol/L at T1 and T3, both PP and MAP at T1, T2 and T3, PSQI, and LMeD adherence at T1, T2 and T3. Model 3 incorporated individual food groups in separate models while controlling for significant variables from model 2. Variable selection was conducted using stepwise regression.

Logistic regression was used to evaluate the predictors of SGA and LGA infants each compared to AGA infants. Model 1-Maternal Characteristics - controlled for variables such as family history of diabetes, smoking at T1, T2, T3, parity, infant sex, maternal height, history of macrosomia, both MAP and PP at T1, T2, and T3, PSQI, pre-pregnancy BMI and total GWG,

Page: 19

Author: Shrish Budree Date: 12/3/2025 9:52:53 PM

did the authors look at impact of these maternal characteristics on pre-term delivery and LBW in addition to SGA/IUGR. Prematurity and LBW have significant impact on infant mortality and morbidity

Author: Shrish Budree Date: 12/3/2025 10:04:26 PM

did any of the models include the EPDS? Did the authors consider including socio-economic status as a co-variate and if not, why?

Author: Shrish Budree Date: 12/6/2025 9:29:39 AM

What is the rationale for further adjusting for these variables?

and LMeD adherence at T1, T2 and T3. Model 2- Individual Food Groups included significant variables from Model 1 and individual food groups, with final adjustments made via the forward Wald method.

Results

General population and newborn characteristics

The study sample comprised 618 pregnant women; average age was 29.2 ± 5.0 years, average parity was 1.3 children, 49.2% were homemakers and 48.7% were employed. Fewer than half resided in the main urban areas of Mount Lebanon and Beirut, with a greater percentage living in rural regions. The majority had a university degree (76.1 %,); 23.9% had a high school education or less. Monthly income ranged from $700--$2000 and varied little by region. Pre-pregnancy BMI was normal for 67.6% of women; 32.4% were overweight or obese. Excessive weight gain was high for most women with 86.8% in T1 and 93.2% in T3, but most had no history of gestational diabetes mellitus (98%) or hypertensive disorders during pregnancy (96.4%) whereas anemia was common (25.0%). The majority of infants (72.5%) were AGA births; 11.8% were SGA and 15.9% were LGA (Table 1). Mean head circumference was 33.20 ± 3.17 cm, and mean abdominal circumference was 27.44 ± 3.35 cm. Apgar scores averaged 8.31 ± 1.06 at 1 minute and 9.55 ± 1.07 at 5 minutes

This page contains no comments

Table 1. Population characteristics of pregnant women in Lebanon

**Maternal health characteristics**[a]	**Mean ± SD or %**
Pre-pregnancy BMI categories, kg/m²	
Underweight (<18.5)	3.9
Normal (18.5–24.9)	63.4
Overweight (25.0–29.9)	22.5
Obese (≥30.0)	10.2
Total GWG, kg	
Low	11.1
Adequate	20.5
Excessive	68.3
GWG at T1, kg	1.17 ± 4.33
Low	3.6
Adequate	9.6
Excessive	86.8
GWG at T2, kg	5.34 ± 3.46
Low	34.5
Adequate	4.1
Excessive	61.4
GWG at T3, kg	7.09 ± 3.80
Low	4.5
Adequate	2.3
Excessive	93.2
**Newborn characteristics**	
Infant sex	
Boy	58.
Girl	42
Gestational age at delivery, weeks	37.6 ± 1.9
Mean birth weight, grams	3118.± 598
Birth weight, grams	
<2500 g	11.0
2500–4500 g	85.6
≥4500 g	3.4
Birth weight z score,	
SGA (<10th percentile)	11.8
AGA (10th–90th percentiles)	72.3

Page: 21

Author: Shrish Budree Date: 12/3/2025 10:09:52 PM

Need to indicate which are means and which are percentages for clarity

Author: Shrish Budree Date: 12/3/2025 10:11:10 PM

suggest 'male', 'female'

LGA (>90th percentile)	15.9

[a]Values are presented as means ± standard deviations unless otherwise specified. Percentages are shown for categorical variables. N = 618; 42 participants dropped out due to miscarriages and/or other reasons.

Trimestral comparisons for maternal factors among SGA, AGA and LGA infants (Table 2)

Table 2 compares maternal factors by trimester among SGA, AGA and LGA infants. Factors that differed revealed that greater adherence to the LMeD was associated in both trimesters 2 ($p<0.033$) and 3 ($p<0.038$), but not in trimester 1, with birth of an AGA compared to a SGA infant. In contrast, GWG in T1 was associated with birth of an LGA infant ($p<0.011$). With regards to blood pressure, higher mean arterial pressure (MAP) in T1 and T3 ($p<0.016$ and $p<0.002$, respectively) and higher PP ($p<0.002$, $p<0.016$, $p<0.001$) in all 3 trimesters were associated with birth of an SGA infant. In contrast, lower PP across all trimesters were observed in mothers who gave birth to LGA infants. Most women were sedentary, and mothers who gave birth to LGA infants remained largely inactive in T2.

With regards to psychosocial variables, poor sleep in T2, which was more prevalent among women who subsequently gave birth to LGA infants (86.5%, $p<0.038$), was the only psychosocial variable to emerge as significantly related to birth weight. Interestingly, other psychological variables including stress and depression did not differ across birth weight categories, but their prevalence exceeded 50% of mothers in T1, T2 and T3. Further variables were analyzed reported in Supplemental Table 1. Gestational age, weight at T3, delivery

Page: 22

Author: Shrish Budree Date: 12/6/2025 9:14:27 AM

Did this correlate with hypertension during pregnancy knowing that gestational hypertension is associated with SGA

Author: Shrish Budree Subject: Highlight Date: 12/7/2025 4:18:09 PM

Not sure why this was tested as it is expected that birth weight will correlate with GA

Author: Shrish Budree Subject: Highlight Date: 12/7/2025 4:17:33 PM

clarify as 'maternal weight'

complications, and previous macrosomia were significantly different among SGA, AGA and LGA ($p<0.05$).

Supplemental Table 2 compares the individual food group consumption among SGA, AGA and LGA. Mothers of LGA infants had the highest consumption of eggs and olive oil, whereas mothers of SGA infants had the lowest consumption of dairy products and vegetables. Interestingly, dairy product consumption was highest among AGA infants ($p<0.05$)

This page contains no comments

Table 2. Comparisons of maternal health factors across the SGA, AGA, and LGA groups across trimesters

Variables [a]	SGA (N=73)	AGA (N=447)	LGA (N=98)	p*
**Maternal Characteristics [b]**				
Adherence to the LMeD T1	16.6 ± 4.2	17.5 ± 3.9	18.0 ± 3.0	0.205
Adherence to the LMeD T2	16.5 ± 4.2 [A]	18.5 ± 3.9 [B]	18.0 ± 4.9 [AB]	0.033*
Adherence to the LMeD T3	16.5 ± 4.9 [A]	18.8 ± 4.4 [B]	18.2 ± 5.2 [AB]	0.038*
Mean GWG T1, kg	1.29 ± 2.49 [A]	1.55 ± 4.19 [A]	3.02 ± 5.66 [B]	0.011*
Mean GWG T2, kg	4.29 ± 3.00	5.43 ± 3.62	5.52 ± 2.74	0.059
Mean GWG T3, kg	6.22 ± 3.69	7.10 ± 3.78	7.64 ± 3.85	0.100
MAP T1, mm Hg	132.86 ± 12.86 [A]	130.02 ± 12.67 [B]	126.78 ± 14.42 [B]	0.016*
MAP T2, mm Hg	133.25 ± 14.80	131.79 ± 12.08	129.05 ± 14.25	0.104
MAP T3, mm Hg	140.31 ± 18.24 [A]	134.06 ± 12.52 [B]	135.67 ± 12.70 [B]	0.002*
Pulse pressure T1, mm Hg	25.69 ± 18.17 [A]	21.04 ± 18.28 [B]	15.23 ± 19.08 [B]	0.002*
Pulse pressure T2, mm Hg	25.15 ± 17.36 [A]	20.50 ± 17.53 [B]	16.44 ± 20.95 [B]	0.016*
Pulse pressure T3, mm Hg	27.74 ± 20.57 [A]	21.16 ± 18.86 [B]	15.99 ± 19.02 [B]	0.001*
Physical activity at T2				0.027*
Sedentary	80.3% [A]	88.8% [A]	94.4% [B]	
Active	19.7%	11.2%	5.6%	
Physical activity at T3				0.131
Sedentary	90.2%	94.6%	97.7%	
Active	9.8%	5.4%	2.3%	
FBG T1, mg/dL	94.19 ± 11.04	92.39 ± 8.54	92.95 ± 8.57	0.335
FBG T3, mg/dL	95.59 ± 11.15	95.29 ± 10.67	96.44 ± 11.75	0.686
IGT T1 (FBG ≥5.51 mmol/L.)				0.776
Yes	12.3%	10.3%	12.2%	
No	87.7%	89.7%	87.8%	
IGT T3 (FBG ≥5.51 mmol/L.)				0.872
Yes	21.9%	24.6%	23.5%	
No	78.1%	75.4%	76.5%	
**Psychosocial Variables**				
Sleep score T1				0.323
Bad Sleep (Score ≥5)	57.5%	50.6%	57.1%	
Good Sleep (Score <5)	42.5%	49.4%	42.9%	
Sleep score T2				0.038*
Bad Sleep (Score ≥5)	75.0% [A]	73.8% [A]	86.5% [B]	
Good Sleep (Score <5)	25.0%	26.2%	13.5%	
Sleep score T3				0.350
Bad Sleep (Score ≥5)	83.3%	82.3%	88.6%	
Good Sleep (Score <5)	16.7%	17.7%	11.4%	
Mean Perceived Stress Score T1	18.61 ± 6.04	19.68 ± 5.92	20.55 ± 6.03	0.11
Perceived Stress Score T1				0.160
Low (Score ≤13)	24.7%	16.3%	13.3%	
Moderate (Score 14–26)	68.5%	73.2%	71.4%	

Page: 24

Author: Shrish Budree Date: 12/4/2025 9:41:50 PM

clarify that the p value column is the one way anova p value. Clarify which pairs are being compared with superscripts A, B, AB

High (Score ≥27)	6.8%	10.5%	15.3%	
Mean Perceived Stress Score T2	20.26 ± 5.62	20.46 ± 6.45	22.17 ± 6.57	0.060
Perceived Stress Score T2				0.125
Low (Score ≤13)	14.8%	16.7%	11.2%	
Moderate (Score 14–26)	75.4%	66.9%	64.0%	
High (Score ≥27)	9.8%	16.4%	24.7%	
Mean Perceived Stress Score T3	20.13 ± 6.29	21.43 ± 6.98	21.92 ± 7.14	0.283
Perceived Stress Score T3				0.343
Low (Score ≤13)	19.7%	15.1%	12.5%	
Moderate (Score 14–26)	65.6%	62.6%	59.1%	
High (Score ≥27)	14.8%	22.3%	28.4%	
Mean Edinburgh Depression Scale T1	9.15 ± 5.00	9.73 ± 4.91	9.18 ± 5.49	0.465
Edinburgh Depression Scale T1				0.809
Non-Depressed (Score <10)	52.1%	51.0%	54.6%	
Depressed (Score ≥10)	47.9%	49.0%	45.4%	
Mean Edinburgh Depression Scale T2	10.38 ± 4.34	9.67 ± 4.80	10.00 ± 5.60	0.523
Edinburgh Depression Scale T2				0.149
Non-Depressed (Score <10)	31.7%	44.7%	45.5%	
Depressed (Score ≥10)	68.3%	55.3%	54.5%	
Mean Edinburgh Depression Scale T3	10.65 ± 5.09	9.41 ± 4.94	9.63 ± 5.88	0.216
Edinburgh Depression Scale T3				0.326
Non-Depressed (Score <10)	38.3%	48.6%	48.3%	
Depressed (Score ≥10)	61.7%	51.4%	51.7%	

[a] N= 618 Values are presented as means ± standard deviations unless otherwise specified. Percentages are shown for categorical variables.
[b] Superscript letters ([A], [B], [AB]) indicate significant pairwise differences between groups based on Tukey's post hoc test following one-way ANOVA.
*$p < 0.05$ indicates statistical significance.

Adjusted linear regression models for birth weight for gestational age (BWGA) and appropriate for gestational age (AGA)

Table 3 summarizes linear regression analyses of predictors for infant birth weight for gestational age (BWGA, n=618) and determinants of AGA infants among 447 pregnant women.: In Model 1-Maternal Characteristics revealed that parity, pre-pregnancy BMI, history of macrosomia, and total GWG significantly increased BWGA. Infant sex also played a role, with girls having lower BWGA. In Model 2- that included additional clinical variables such as

Page: 25

Author: Shrish Budree Subject: Highlight Date: 12/7/2025 4:25:42 PM

Clarify if this was significant

Author: Shrish Budree Subject: Highlight Date: 12/7/2025 4:24:53 PM

suggest 'significantly associated with increased BWGA'

GDM, IGT: FBG≥5.6 mmol/L, MAP, PP, PSQI, and adherence to the LMeD uncovered in addition to parity, history of macrosomia, infant sex, total GWG, pre-pregnancy BMI that both PP and PSQI were also significant. However, adherence to the LMeD did not enter the model. In Model 3-Individual Food Groups, specific food groups emerged as significant determinants of BWGA revealing that higher intakes of olive oil, dairy products, and eggs in the third trimester, in addition to poorer quality sleep, were associated with increased BWGA. Infant sex continued to be negatively associated with BWGA, with girls having lower birth weights.

In contrast, for the AGA infants, Model 1-Maternal Characteristics showed that only parity, and not pre-pregnancy BMI, total GWG, infant sex, history of macrosomia, or PP, was associated with an AGA birth. Model 2-Maternal Health Factors confirmed that higher parity was associated with birth of an AGA infants, Model 3- Individual Food Groups which focused on individual foods revealed that adherence to the LMeD in T1 was inversely associated with AGA whereas higher intakes of dried fruits in T3 increased AGA birth weights, whereas higher dairy product intake lowered it in T1. Neither olive oil nor intake of eggs entered the models for birth of an AGA infant.

Page: 26

Author: Shrish Budree Subject: Highlight Date: 12/7/2025 4:27:48 PM

clarify whether this is higher parity

Author: Shrish Budree Subject: Highlight Date: 12/7/2025 4:29:41 PM

Clarify if this means that LMED adherence was not significantly associated with BWGA

Author: Shrish Budree Subject: Highlight Date: 12/7/2025 4:33:00 PM

Suggest new sentence

Table 3. Linear regression describing determinants of infant birth weight-for-gestational age (%) (N=618) and AGA Infants (=447) in pregnant women in Lebanon

Variable	Adjusted Model of BWGA[a]			Adjusted Model for AGA[b]		
	B	SE	p*	B	SE	p*
**Model 2 – Maternal Health Factors**						
Parity, #	3.701	1.104	0.001	3.291	1.156	0.005
Infant sex, M=0 / F=1	-9.841	2.540	0.000	--	--	--
Hx Macrosomia (No=0 / Yes=1)	19.736	7.014	0.005	--	--	--
Total GWG, kg	0.960	0.225	0.000	--	--	--
Pre-pregnancy BMI, kg/m²	0.713	0.291	0.015	--	--	--
Pulse pressure T2, mm Hg	0.397	0.152	0.009	--	--	--
Pulse pressure T3, mm Hg	-0.491	0.144	0.001	--	--	--
PSQI T2	1.169	0.380	0.002	0.674	0.336	0.045
**Model 3 – Individual Food Groups**						
Parity, #	4.017	1.096	0.000	--	--	--
Infant sex, M=0 / F=1	-8.990	2.525	0.000	--	--	--
Hx Macrosomia (No=0 / Yes=1)	15.466	6.574	0.019	--	--	--
Total GWG, kg	0.765	0.216	0.000	--	--	--
PSQI T2	1.088	0.379	0.004	--	--	--
Olive Oil T3, servings/day	2.898	1.279	0.024	--	--	--
Parity, #	3.934	1.095	0.000	3.293	1.150	0.004
Infant sex, M=0 / F=1	-9.169	2.513	0.000	--	--	--
Hx Macrosomia (No=0 / Yes=1)	16.186	6.518	0.013	--	--	--
Total GWG, kg	3.934	1.095	0.000	--	--	--

Page: 27

Author: Shrish Budree Subject: Highlight Date: 12/7/2025 4:40:17 PM

Can the authors please clarify this model - is this a logistic regression model with AGA as the dependent variable?

Author: Shrish Budree Subject: Highlight Date: 12/7/2025 4:45:06 PM

why is history of macrosomia repeated?

PSQI T2	1.171	0.379	0.002	--	--	--
Dairy products T1, servings/day	-3.132	0.955	0.001	-1.892	0.690	0.006
Dairy products T2, servings/day	2.656	1.201	0.027	--	--	--
Parity, #	3.738	1.102	0.001	--	--	--
Infant sex, M=0 / F=1	-9.119	2.518	0.000	--	--	--
Hx Macrosomia (No=0 / Yes=1)	13.814	6.621	0.037	--	--	--
Total GWG, kg	0.733	0.216	0.001	--	--	--
PSQI T2	1.131	0.378	0.003	--	--	--
Eggs T3, servings/day	7.279	2.587	0.005	--	--	--
Parity, #	--	--	--	3.726	1.168	0.002
Dried fruits T3, servings/day	--	--	--	6.324	2.564	0.014
Adherence to the LMeD T1	--	--	--	-3.807	1.843	0.040

[a] N=618 for BWGA model and [b] N=447 for AGA model.
*Only significant adjusted variables are shown.
"--" indicates non-significant associations

Multiple logistic models for SGA with AGA, and LGA with AGA

SGA vs. AGA: Multiple logistic regression models were used to assess predictors of SGA compared with AGA infants (n=520). Model 1-Maternal Characteristics, which included maternal characteristics, revealed that higher total GWG was associated with a decreased risk of SGA [OR=0.894, p<0.001], whereas higher MAP in the third trimester increased the risk of SGA [OR=1.047, p<0.004]. After adjustment, total GWG and MAP during the second and third trimesters remained significant predictors (Table 4).

Model 2-Individual Food Groups focused on specific food groups, identifying burghul (bulgur wheat) as a significant factor. Burghul consumption in the first trimester was associated with

Page: 28

Author: Shrish Budree Subject: Highlight Date: 12/7/2025 4:47:13 PM
clarify why history of macrosomia is repeated 3 times

Author: Shrish Budree Subject: Highlight Date: 12/7/2025 4:50:57 PM
clarify - significant predictor of what? SGA?

Author: Shrish Budree Subject: Highlight Date: 12/7/2025 4:57:01 PM
Clarify - significant factor for what?

increased SGA risk [OR=16.329, p<0.024]. This association persisted after adjusting for total GWG and MAP in T2 and T3.

Table 4. Multiple logistic regression describing the likelihood of SGA to AGA infants in pregnant women in Lebanon (N=520)

	**Unadjusted Model**			**Adjusted Model**		
**SGA[a]**	**OR**	**CI**	**p***	**OR**	**CI**	**p***
**Model 1 - Maternal characteristics**						
Total GWG, kg	0.894	0.329-0.781	0.001	0.511	0.337-0.774	0.002
MAP T2, mm Hg	--	--	--	0.958	0.928-0.990	0.000
MAP T3, mm Hg	1.047	1.015-1.078	0.004	1.045	1.017-1.073	0.001
Pulse pressure T3, mm Hg	1.039	1.002-1.076	0.037	--	--	--
**Model 2 - Individual Food Groups**						
Total GWG, kg	0.430	0.267-0.695	0.001	0.483	0.315-0.740	0.001
MAP T2, mm Hg	0.948	0.909-0.988	0.012	0.956	0.925-0.987	0.007
MAP T3, mm Hg	1.047	1.012-1.084	0.009	1.049	1.020-1.079	0.001
Burghul T1, Serving/day	16.329	1.440-185.213	0.024	3.130	1.145-8.560	0.026

[a] N=520
*Only significant adjusted variables are shown.
"--" indicates non-significant associations

LGA vs AGA: Multiple logistic regression models were used to assess predictors of LGA infants compared with AGA infants (n=545) (Table 5). Model 1-Maternal Characteristics revealed that

This page contains no comments

higher parity and previous macrosomia were associated with increased odds of LGA infants [OR=1.369, p<0.010; OR=8.482, p<0.001], whereas being a girl was associated with a lower likelihood of LGA [OR=0.340, p<0.002]. Higher MAP in the third trimester was linked to increased LGA risk [OR=1.039, p<0.016]. After adjustment using the stepwise forward model, parity, infant sex, and previous history of macrosomia remained significant, while family history of diabetes and higher pulse pressure (PP) in the first trimester were associated with a decreased risk of LGA [OR=0.456, p<0.041; OR=0.977, p<0.007].

Model 2-Individual Food Groups identified olive oil consumption as a significant predictor of LGA. A higher intake of olive oil was associated with an increased risk of LGA [OR=1.447, p<0.010], along with significant associations with parity, infant sex, and previous history of macrosomia. (Table 5)

Author: Shrish Budree Subject: Highlight Date: 12/7/2025 5:01:46 PM
Were maternal factors such as gestational diabetes or imparied glucose tolerance associated with LGA? This would be an expected finding.

Table 5. Multiple logistic regression describing the likelihood of LGA to AGA infants in pregnant women in Lebanon (N=458)

	**Unadjusted Model**			**Adjusted Model**		
**LGA [a]**	**OR**	**CI**	**p***	**OR**	**CI**	**p***
**Model 1 - Maternal characteristics**						
Parity, #	1.369	1.077-1.740	0.010	1.452	1.165-1.811	0.001
Infant sex, M=0/F=1	0.340	0.173-0.670	0.002	0.314	0.166-0.594	0.000
Family Hx DM, No/Yes	--	--	--	0.456	0.215-0.968	0.041
Hx Macrosomia, No/Yes	8.482	2.533-28.407	0.001	6.447	2.145-19.376	0.001
MAP T3, mm Hg	1.039	1.007-1.072	0.016	--	--	--
Pulse pressure T1, mm Hg	--	--	--	0.977	0.961-0.994	0.007
PSQI T3	--	--	--	1.081	1.000-1.167	0.049
**Model 2 - Individual Food Groups**						
Parity, #	1.367	1.074-1.740	0.011	1.498	1.208-1.859	0.000
Infant sex, M=0/F=1	0.327	0.165-0.650	0.001	0.352	0.187-0.663	0.001
Hx Macrosomia, No/Yes	8.321	2.433-28.460	0.001	6.145	2.231-16.928	0.000
Olive oil T2, Serving/day	--	--	--	1.447	1.094-1.913	0.010

[a] N=458
*Only significant adjusted variables are shown.
"--" indicates non-significant associations

This page contains no comments

Discussion

This national cohort study is the first in Lebanon to explore the associations between the adherence to the LMeD, psychosocial, and maternal variables by trimester with 3 birth weight categories: appropriate (AGA), small (SGA), and large-for-gestational age (LGA) infants. Several novel features emerged as determinants of these infant birth weight categories. Adherence to the LMeD in T1 was associated with birth of AGA infants. In contrast, adherence to LMeD did not enter models for SGA or LGA but intakes of specific foods and maternal health status indicators did. For SGA infants, an appropriate GWG mitigated against low birth weight whereas higher burghul intake in T1 and elevated MAP in T2 and T3 were linked to increased SGA risk. For LGA infants, previous macrosomia and poor sleep quality in T3, and higher intake of olive oil in T2 were associated with higher risk of an LGA birth whereas higher pulse pressure in T1 was associated with lower odds of a LGA birth. Collectively, results show that adherence to the LMeD is associated with birth of an AGA infant, whereas GWG and undiagnosed blood pressure perturbations related to eMAP and PP, and poor food choices increase the likelihood of SGA and LGA births.

Adherence to LMeD and infant birth weight

Page: 32

Author: Shrish Budree Subject: Highlight Date: 12/7/2025 5:14:38 PM

Suggest 'were not significant associated with' or similar

Author: Shrish Budree Subject: Highlight Date: 12/7/2025 5:16:57 PM

Low birth weight (ie. birth weight <2500g) was not included as a dependent variable in any model. Suggest rewording

Author: Shrish Budree Subject: Highlight Date: 12/7/2025 5:18:22 PM

Clarify 'eMAP'

BWGA: Our first objective had been to characterize the determinants of BWGA in Lebanon. Nearly 40 years earlier, Kramer had concluded that major predictors of BWGA in both developed and developing were parity, infant sex, pre-pregnancy weight and total GWG [52]; in 2009, an additional modification was incorporated when the Institute of Medicine (IOM) and the National Research Council (NRC) included pre-pregnancy BMI and created targeted GWG guidelines by pre-pregnancy BMI [53]. Today, given the widespread attention to the health benefits of the MeD during pregnancy [54], we were curious to investigate in Lebanon, a LMIC undergoing a nutrition transition from a Mediterranean to a more Westernized diet, if adherence to the LMeD would confer health benefits in this dynamically changing food environment. We were able to confirm in this large healthy, university educated population with access to health care that predictors of BWGA did include parity, infant sex, pre-pregnancy BMI and total GWG but also revealed that sleep quality, history of macrosomia, pulse pressure in T1 and mean arterial pressure in T2 and T3 emerged and were associated with infant birth weights. Surprisingly, adherence to the LMeD in each trimester did not enter models for overall BWGA, but individual food groups did.

AGA: In sharp contrast with BWGA, a key observation was that greater adherence to the LMeD, particularly in the second and third trimesters, was associated with birth of AGA infants, confirming earlier reports highlighting the health benefits of a MeD for normal fetal growth [55] and evidence that greater adherence to the MeD was associated with increased birth weight and a lower risk of SGA infants [56, 57]. However, other studies highlighted that the first trimester is a critical window for promoting the benefits of the MeD during pregnancy [58] Importantly, our

This page contains no comments

adjusted regression models contrasting determinants of BWGA with AGA infants further demonstrated that adherence to LMeD in T1 emerged and was a determinant of an AGA birth.

Mechanistically, strong adherence to a MeD in the first trimester has been associated with a lower resistance of the uterine and umbilical arteries in the second and third trimester [59, 60]. Adherence to a MeD during pregnancy has also been associated with higher total antioxidant capacity [61] whereas low adherence was associated with high cord-blood insulin levels and insulin resistance at delivery [62].

There is also evidence that adherence to a MeD throughout pregnancy is associated with several health benefits for the mother that benefit the infant. A recent meta-analysis describing these health benefits of the MeD during pregnancy reported improved maternal outcomes including lower prevalences of maternal obesity and overweight, GDM, hypertension and improved sleep [63] reinforcing the health benefits of early maternal adherence to the LMeD beginning in T1 in promoting healthy AGA infant birth weights for gestational age as none of these risk factors emerged as determinants of an AGA birth if mothers adhered to the LMeD.

Association of specific food groups with BWGA outcomes: AGA, SGA and LGA

Page: 34

Author: Shrish Budree Subject: Highlight Date: 12/7/2025 5:28:42 PM

reference table that author is referring to here

Author: Shrish Budree Subject: Highlight Date: 12/7/2025 5:51:45 PM

Unclear - suggest rewording

As adherence to the LMeD was only associated with birth of an AGA infant and not with either SGA or LGA births, it was interesting to observe that specific food groups entered models for the 3 birth weight categories, highlighting that both quality and quantity of specific foods associated with the LMeD were important. In our study, these five food groups were olive oil, dairy products, eggs, dried fruits, and burghul.

Olive oil intake is an important cornerstone of MeD. Numerous studies have reported favorable outcomes with regular olive oil consumption as part of the MeD [64, 65]. However, in our study, this food group was also associated with higher BWGA in T3 and an increased likelihood of a LGA birth, suggesting that excess fat intake specifically in the second half of pregnancy — even healthy sources such as olive oil — could contribute to greater fetal growth. A recent study from Iran showed that a low intake ratio of monounsaturated to saturated fatty acids was inversely associated with GWG, suggesting that not only the quantity but the quality of the fat intake in a MeD is important during pregnancy [66]. This was observed in our study with higher olive intake in T2 and T3 among women who delivered LGA infants.

Dairy product consumption in our study was inversely associated with BWGA and AGA births in T1 but positively associated with BWGA in T2, reinforcing earlier studies where milk intake was associated with higher BWGA and both lower risk of SGA and higher risk of LGA births [67, 68]. Others have reported that low dairy intake during pregnancy is associated with risk of an SGA birth and that as little as 100g/day during the first half of pregnancy decreased the

Author: Shrish Budree Subject: Highlight Date: 12/7/2025 5:57:03 PM

For clarity, can the authors please clarify whether they are referring to higher or lower BWGA which referencing associations

likelihood of a SGA birth [69]. However higher intakes of dairy products in the first trimester have been associated with greater fetal weight gain in the third trimester and higher birth weights [70, 71]. Some authors had linked the association to the milk protein content and not to the fat or carbohydrate fraction of milk [71] whereas others had highlighted particularly whey proteins [72].

Milk is also recognized as having an abundant supply of insulin like growth factors (IGFs) [73]. IGF-1 levels have been positively associated with birth weight [74]. Interestingly, these authors also reported that among maternal demographic and anthropometric characteristics, only parity was correlated with cord IGF-1 concentrations. In our study, we also observed that parity was the only maternal factor consistently associated with an AGA birth.

Egg consumption was associated in our study with an increased BWGA in T3. Their inclusion in the maternal diet does contribute to overall nutrient adequacy, however excessive consumption as seen in previous research by Xue et al. 2024 contributed to the birth of an LGA infant [75].

Dried fruits, when consumed during the third trimester, were positively associated with AGA birth weights. This observation aligns with findings from earlier studies that identified improved pregnancy outcomes with diets high in dried fruits and legumes [76]. It is presumed that the nutrient density and antioxidant content of dried fruits may play a protective role in achieving optimal fetal size and as part of the MeD may enhance placental function and nutrient transfer [60, 77]. More recently, a revised systematic review and dose response meta-analysis highlighted

Author: Shrish Budree Subject: Highlight Date: 12/7/2025 6:02:07 PM

This reference to the association of parity with birth weight is oddly placed here and has no association with dairy intake in pregnancy. Suggest removing

the role of date palm fruit during the final month of gestation for promoting maternal and neonatal health [78].These authors noted some improvements in parturition and breastfeeding practices, but concluded that further exploration of cultural foods during pregnancy is warranted.

Burghul (bulgur wheat) intake during the first trimester was unexpectedly linked to increased risk of SGA. Previous research has shown that high fiber, vegetarian or grain-heavy dietary patterns may reduce birth weight [56, 57]. This finding warrants further investigation to determine whether higher burghul intake is a proxy for limited dietary diversity or dietary restrictions due to nausea or socioeconomic factors and food availability.

Pre-pregnancy BMI, gestational weight gain (GWG) and birth outcomes

Previous research in Lebanon had linked pre-pregnancy overweight and obesity to increased risk of LGA or macrosomia [79], but this association was not observed in our national cohort. In our study, more than 67% of mothers were classified as having normal weights or were underweight before pregnancy whereas overweight and obese women constituted slightly more than 32% of the study population. However, regardless of pre-pregnancy BMI, more than 86% exceeded GWG guidelines in T1, 61. 4% in T2 and 93.4% in T3. Moreover, overweight and obese pregnant women exceeded the recommended GWG, whereas underweight women rarely

This page contains no comments

did—a pattern observed in other LMICs [80, 81, 82]. Despite exceeding GWG, the mean total GWG was 14.17±5.71 among the AGA infants which constituted 72% of the study population.

Total GWG

Our study further reinforces the critical role of total GWG in determining infant birth weight. As expected, higher overall GWG was associated with reduced risk of SGA and higher birth weights, consistent with global evidence from (LMICs) [17]. These findings align with WHO and IOM guidelines that emphasize adequate weight gain as a modifiable factor supporting optimal fetal development as per WHO and IOM guidelines [53]. However, recommendations also emphasize that little weight gain is required in the first trimester and several countries have incorporated these recommendation into their national recommendations [83]. These observations highlight the critical need for targeted nutritional counseling and weight monitoring early in and throughout pregnancy.

First trimester GWG

Weight gain in the first trimester reflects both maternal nutritional reserves and early placental development [84]. Insufficient gain during this period may signal poor nutrient availability during organogenesis, potentially leading to restricted fetal growth and placental

This page contains no comments

development [77]. Our findings identified an important impact of GWG during the first trimester (T1) on birth weight, which reinforces observations associated with trimestral GWG patterns related to both SGA and LGA infants [80, 85]. While several studies have emphasized total or late-pregnancy GWG below or above IOM recommendations [80, 86], data also show that early gestational weight changes may have lasting implications on fetal growth trajectories regardless of GWG later in pregnancy and may in some instances even override the influence of pre-pregnancy BMI [86, 87]. Thus, the timing of the GWG cannot be overlooked as GWG in T1 and total GWG throughout pregnancy may play an underappreciated role in maternal and placental health and ultimately impact SGA, AGA and LGA birth.

Blood pressure and fetal growth

Mean arterial pressure

Hypertensive disorders of pregnancy affect approximately 10% of pregnant women worldwide and are traditionally defined as having a systolic:dystolic blood pressure >140:90 mmHg [47]. However, some have questioned whether lower cutoffs should be considered [88]. In our study, most women had SBP<140 (98%) and DBP<90 mmHg (94%). Few exceeded SBP≥140 mmHg or DBP≥90 mmHg mmHg in T1, T2 and T3. In contrast, elevated eMAP was more prevalent, increasing from 24% in T1 to 41% in T2 to 49% in T3, suggesting that eMAP might be a more sensitive indicator of hypertension risk compared to traditional SBP and DBP measurements in our sample of healthy pregnant mothers consuming a MeD that mitigated against other competing determinants of birthweight including GWG and GDM.

This page contains no comments

In our hierarchical modeling, we observed that higher MAP in T2 was associated with lower risk of SGA. This change coincides with the known declines in SBP and DBP in normotensive mothers in T2 [89]. Authors have suggested that a lack of a mid-pregnancy SBP drop might reflect increased vascular resistance in women destined to develop hypertensive pregnancy complications. Elevated MAP in the third trimester was significantly associated with increased SGA risk but not with LGA in our adjusted model. This is consistent with findings from the Generation R study [90] and the NICHD cohort [91], which reported a near threefold increase in SGA risk in women with hypertensive disorders—even after adjusting for other risk factors. Similarly, Iwama et al. [92] found that MAP levels even before 20 weeks can predict SGA risk. These findings support the use of established cut-offs for MAP as an early and continuous monitoring tool in prenatal care.

Pulse pressure

Pulse pressure (PP) is a proxy to measure arterial stiffness. Increased arterial stiffness has been linked to the inability of the cardiovascular system to adapt to the physiological demands of pregnancy including the need for increased blood volume and efficient placental blood flow. This pathology has been associated with intrauterine growth retardation in third trimester [93, 94]. However, in our study PP emerged in T1 and was inversely associated with birth of an LGA infant, possibly underscoring early placental adaptation to increased blood flow and blood volume expansion whereas I then same model, history of macrosomia increased the odds of an LGA birth 6-fold. PP measures during pregnancy in a normotensive population may be an overlooked screening tool for both SGA and LGA births in LMIC, but further research is warranted.

Page: 40

Author: Shrish Budree Subject: Highlight Date: 12/7/2025 6:16:48 PM

Typo - please review

Other determinants of infant birth weight

Parity and infant sex

Female infants consistently had lower birth weights than males, in line with patterns observed globally [95]. However, in our study higher parity was consistently associated with increased likelihood of AGA births, contradicting some prior reports suggesting a negative influence of parity on birth weight [96]. Others have reported that among maternal demographic and anthropometric characteristics, parity was correlated with cord IGF-1 concentrations [97]. In our study, parity was the only maternal factor consistently associated with an AGA birth, suggesting that IGF-1 plays a sustaining role during fetal growth.

History of macrosomia

In our study, history of macrosomia was positively associated with BWGA. Studies have demonstrated that women with a priori macrosomic birth have a markedly higher risk of recurrence. For instance, research indicates that these women are 21% more likely to deliver another macrosomic infant compared to those without this history [98], underscoring the importance of considering obstetric history when assessing risks for elevated BWGA. Although macrosomia is often associated with GDM and unregulated FBG, we found no associations between FBG and GDM as they did not enter our models for any infant birth weight category. However, it is important to note that only a small percentage of women in this cohort developed

Page: 41

Author: Shrish Budree Subject: Highlight Date: 12/7/2025 6:19:35 PM

clarify that authors are referring to higher parity

Author: Shrish Budree Subject: Highlight Date: 12/7/2025 6:20:00 PM

prior

GDM (5.6%) which is lower than the percentage reported in other Mediterranean countries [64]. Moreover, the average FBG in trimesters 1 and 3 was less than 5.51 mmol/L. Thus, the majority of our cohort had normal FBG and were considered healthy. This contrasts with findings from other Mediterranean countries, where GDM has been linked to an increased risk of LGA or SGA infants [29, 30]

Sleep

Among sleep, stress, and depression, only poor sleep quality emerged as another important determinant of birth weight for LGA infants. Recent studies in high-income countries have linked poor sleep with increased maternal cortisol levels, impaired glucose metabolism, and gestational diabetes—all of which can contribute to fetal macrosomia [99, 100]. In the U.S, Reutrakul and Van Cauter (2018) reported that women experiencing sleep deprivation or sleep-disordered breathing were more likely to deliver a LGA infant [101]. One study in New Zealand reported that poor sleep quality was associated with adverse pregnancy outcomes and elevated birth weights [102]. However, in LMIC settings, the relationship among MedD, sleep quality and birth outcomes remains underexplored.

Clinical implications

In the context of Lebanon's ongoing economic crisis and strained health infrastructure, the study's implications are highly relevant as it highlights affordable, feasible screening tools including weight gain tracking, blood pressure monitoring, and brief

Author: Shrish Budree Subject: Highlight Date: 12/7/2025 6:23:30 PM

A normal mean blood glucose across trimesters 1 and 3 do not necessarily indicate that the majority of the study population were healthy. What proportion of participants had an abnormal FBG?

Author: Shrish Budree Subject: Highlight Date: 12/7/2025 6:30:19 PM

Authors should comment on why this study did not find an association of maternal depression with birth outcomes given the high prevalence of depression during pregnancy (as have been previous describe in other birth cohort studies).

Author: Shrish Budree Subject: Highlight Date: 12/7/2025 6:27:21 PM

The finding in this study contradicts these previous study finding as there was a low prevalence of GDM and impaired glucose tolerance. Therefore, not sure that this is relevant.

psychosocial assessments that can be integrated into routine healthcare protocols without the need for sophisticated equipment or high-cost interventions. These findings offer a pathway toward more personalized, preventive prenatal care that targets both biological and psychosocial determinants of birth weight.

The high prevalence of excessive GWG among most women reinforces the need for BMI-specific GWG monitoring, which can be implemented with simple weight charts or digital tools. Trimestral calculation of MAP and PP in normotensive mothers to be may serve as an early warning system for identification of women at risk for SGA and LGA births, thus facilitating timely interventions to reduce these adverse outcomes. Finally, the association between poor sleep quality and risk of LGA adds a novel psychosocial dimension, suggesting the possible incorporation of sleep quality screening questionnaires such as the Pittsburgh Sleep Quality Index during clinic visits.

Strengths and limitations

One of the major strengths of this national prospective cohort study lies in its longitudinal and regionally representative design, capturing diverse maternal characteristics across three distinct time points in pregnancy. This enabled not only temporal analysis stressing the health promoting benefits of adherence to the MeD for both mothers and infants and also provided insight into the dynamic progression of key clinical markers, such as GWG and blood pressure. The inclusion established predictors of birth weight, diet, and maternal psychosocial

Author: Shrish Budree Subject: Highlight Date: 12/7/2025 6:33:54 PM

The findings of high MAP associated with LGA is unusual. Current evidence shows that mean arterial pressure (MAP) in pregnancy is primarily studied as a predictor of hypertensive disorders and fetal growth restriction, not high birth weight or large-for-gestational-age (LGA) infants

Author: Shrish Budree Subject: Highlight Date: 12/7/2025 6:35:49 PM

A more important question is whether the PSQI is a validated tool in pregnancy and perhaps this is a spurious funding

Author: Shrish Budree Subject: Highlight Date: 12/7/2025 6:37:35 PM

check grammar

variables adds to the robustness and generalizability of the findings Using a hierarchical modeling approach, we were able to identify GWG and MAP as strong, measurable predictors of birth outcomes — specifically LGA and SGA and to reinforce the health claims associated with adherence to the MeD beginning in the first trimester and continuing throughout pregnancy for the mother-infant dyad. On the other hand, the study has several limitations, such as potential biases from self-reported data and the impact of external factors such as the economic crisis and COVID-19 and the lack of assessment of total energy intake.

Conclusion

In conclusion, this study emphasizes the need for a comprehensive approach in prenatal care that integrates dietary, lifestyle, and health management strategies to optimize infant birth outcomes. Given the high prevalence of excessive GWG, poor sleep quality and variations in dietary intake, targeted interventions that promote healthy eating patterns, better sleep and address weight management are essential. Further research is warranted to explore the long-term implications of these factors for maternal and child health in the Eastern Mediterranean region, specifically Lebanon with particular attention to the socio-economic and cultural factors influencing dietary habits and health behaviors.

Acknowledgments

Author: Shrish Budree Subject: Highlight Date: 12/7/2025 6:40:09 PM

this should include access to healthcare services and monitoring during pregnancy

The authors would like to thank the women participants in this study and the nurses and clinicians.

References

1. Zhu Y, Zhang C. Dietary patterns and risk of gestational diabetes mellitus: a systematic review and meta-analysis. PLoS One. 2016;11(5):e0152794.

2. Khashan AS, et al. The impact of maternal diet on birth weight: a systematic review. BJOG. 2014;121(11):1356-1369.

3. Mendez MA, et al. Maternal dietary patterns and birth weight: a systematic review. Int J Epidemiol. 2017;46(6):2043-2055.

4. Martínez-Galiano JM, et al. Maternal diet and birth outcomes: a systematic review. Nutrients. 2018;10(4):470.

5. Tovar A, et al. Mediterranean diet and pregnancy outcomes. Eur J Clin Nutr. 2020;74(4):481-488.

6. Chatzi L, et al. Mediterranean diet during pregnancy and childhood health outcomes: a systematic review. Nutr Rev. 2012;70(9):491-506.

7. Fernández-Barrés S, et al. Mediterranean diet adherence and infant birth weight. Eur J Clin Nutr. 2019;73(5):660-668.

8. Parlapani E, et al. Mediterranean diet and maternal outcomes: A review of the literature. Arch Gynecol Obstet. 2019;300(6):1425-1433.

9. Saunders DH, et al. Maternal diet and pregnancy outcomes: A review of the Mediterranean diet. Nutrients. 2014;6(5):1919-1932.

10. Smith M, et al. The effects of diet on pregnancy outcomes in Mediterranean populations. Int J Womens Health. 2015;7:231-239.

11. Colón-Ramos U, et al. The Mediterranean diet and maternal and infant health outcomes. Nutr Rev. 2015;73(3):155-170.

This page contains no comments

12. Kjøllesdal MK, Holmboe-Ottesen G. Mediterranean diet and its impact on birth outcomes: Evidence from longitudinal studies. J Nutr. 2014;144(8):1296-1303.

13. Assaf-Balut C, et al. Mediterranean diet and pregnancy outcomes: a review. Nutrients. 2017;9(4):317.

14. Timmermans S, et al. Mediterranean diet and pregnancy outcomes: a systematic review. J Maternal Fetal Neonatal Med. 2012;25(12):2637-2645.

15. Xie W, et al. Early pregnancy adherence to the Mediterranean diet and pregnancy outcomes. J Am Coll Nutr. 2019;38(7):567-575.

16. Falcone V, et al. Mediterranean diet and pregnancy outcomes: a review. Clin Nutr. 2020;39(9):2701-2709.

17. Shepherd A, et al. Gestational weight gain and birth outcomes: A systematic review. J Nutr. 2017;147(7):1365-1373.

18. Black RE, et al. Maternal weight gain and birth outcomes. Lancet. 2019;373(9674):2265-2274.

19. Shen L, et al. The effect of inadequate weight gain during pregnancy on birth weight. Int J Gynaecol Obstet. 2018;141(1):75-81.

20. Shulman GI. Increased insulin resistance during pregnancy: a reappraisal of insulin resistance and metabolic adaptations. Am J Physiol Endocrinol Metab. 2015;308(1):E1-13.

21. Rizzo T, et al. Gestational diabetes and the risk of large-for-gestational-age infants. Obstet Gynecol. 2017;130(6):1203-1212.

22. Zhang Y, et al. Hypertensive disorders in pregnancy and their impact on birth weight: A systematic review. J Hypertens. 2016;34(8):1679-1687.

23. Uzan J, et al. Hypertensive disorders of pregnancy and low birth weight: A systematic review. Am J Obstet Gynecol. 2013;208(2):87-98.

24. Vinter CA, et al. The impact of hypertensive pregnancy disorders on birth outcomes. Hypertension. 2013;62(2):278-283.

25. Vatten LJ, et al. Gestational hypertension, preeclampsia and birth weight: A cohort study. Hypertension. 2013;62(4):771-776.

26. Lisonkova S, et al. Hypertensive disorders and neonatal outcomes: A review of the literature. Acta Obstet Gynecol Scand. 2015;94(7):706-713.

This page contains no comments

27. Brown MA, et al. Hypertension in pregnancy and neonatal complications. J Hypertens. 2018;36(5):1146-1155.

28. Escobar MF, Benitez-Díaz N, Blanco-Londoño I, et al. Synthesis of evidence for managing hypertensive disorders of pregnancy in low middle-income countries: a scoping review. BMC Pregnancy Childbirth. 2024;24(1):622.

29. Xu JC, Coelho Â. Association between body mass index and gestational weight gain with obstetric and neonatal complications in pregnant women with gestational diabetes. Acta Méd Port. 2022;35(10):718-728.

30. Teshome AA, Li Q, Garoma W, et al. Gestational diabetes mellitus, pre-pregnancy BMI and gestational weight gain predict fetal growth and neonatal outcomes. Clin Nutr ESPEN. 2021;42:307-312.

31. Christian LM, Carroll JE, Teti DM, Hall MH. Maternal sleep in pregnancy and postpartum part I: mental, physical, and interpersonal consequences. Curr Psychiatry Rep. 2019;21:1-8.

32. Nuthalapaty FS, et al. Maternal stress and fetal development: A systematic review. JAMA. 2017;177(6):300-309.

33. Dean AG, Sullivan KM, Zubieta J, Delhumeau C. Epi Info 2000: a database, and statistics program for public health professionals using Windows 95, 98, NT, and 2000 computers.

34. Cohen S, et al. Perceived stress scale (PSS10). J Health Soc Behav. 1994;24(4):384-396.

35. Buysse DJ, et al. Pittsburgh Sleep Quality Index (PSQI). Sleep. 1989;12(1):65-72.

36. Cox JL, et al. Edinburgh Postnatal Depression Scale (EPDS). J Psychosom Res. 1987;31(3):193-197.

37. Naja F, et al. FFQ validation for the Lebanese population. Nutr J. 2015;14(1):36-42.

38. Warburton DE, Jamnik VK, Bredin SS, Gledhill N. The physical activity readiness questionnaire for everyone (PAR-Q+) and electronic physical activity readiness medical examination (ePARmed-X+). Health & Fitness Journal of Canada. 2011;4(2):3-17.

39. Agnoli C, Krogh V, Grioni S, et al. A priori-defined dietary patterns are associated with reduced risk of stroke in a large Italian cohort. J Nutr. 2011;141(8):1552-1558.

40. Buckland G, González CA, Agudo A, et al. Adherence to the Mediterranean diet and risk of coronary heart disease in the Spanish EPIC Cohort Study. Am J Epidemiol. 2009;170(12):1518-1529.

This page contains no comments

41. Panagiotakos DB, Pitsavos C, Stefanadis C. Dietary patterns: a Mediterranean diet score and its relation to clinical and biological markers of cardiovascular disease risk. Nutr Metab Cardiovasc Dis. 2006;16(8):559-568.

42. Gerber M. Qualitative methods to evaluate Mediterranean diet in adults. Public Health Nutr. 2006;9(1A):147-151.

43. Trichopoulou A, Orfanos P, Norat T, et al. Modified Mediterranean diet and survival: EPIC-elderly prospective cohort study. BMJ. 2005;330(7498):991.

44. American College of Obstetricians and Gynecologists. Obesity in pregnancy. ACOG Practice Bulletin No. 230. Obstet Gynecol. 2021 Jun;137(6):e128–44. doi:10.1097/AOG.0000000000004395. .

45. American College of Obstetricians and Gynecologists. ACOG Committee Opinion No. 548: Weight gain during pregnancy. Obstet Gynecol. 2013 Jan;121(1):210-2.

46. Li-Zhen L, Yun X, Xiao-Dong Z, Shu-Bin H, Zi-Lian W, Sandra DA, Bin L. Evaluation of guidelines on the screening and diagnosis of gestational diabetes mellitus: systematic review. BMJ Open. 2019 May 30;9(5):e023014.

47. Wright A, Wright D, Ispas CA, Poon LC, Nicolaides KH. Mean arterial pressure in the three trimesters of pregnancy: effects of maternal characteristics and medical history. Ultrasound Obstet Gynecol. 2015 Jun;45(6):698-706.

48. Von Dadelszen P, Ornstein MP, Bull SB, Logan AG, Koren G, Magee LA. Fall in mean arterial pressure and fetal growth restriction in pregnancy hypertension: a meta-analysis. Lancet. 2000 Jan 8;355(9198):87-92.

49. Hale S, Choate M, Schonberg A, Shapiro R, Badger G, Bernstein IM. Pulse pressure and arterial compliance prior to pregnancy and the development of complicated hypertension during pregnancy. Reprod Sci. 2010 Sep;17(9):871-7.

50. Hediger ML, Overpeck MD, Maurer KR, Kuczmarski RJ, McGlynn A, Davis WW. Growth of infants and young children born small or large for gestational age: findings from the Third National Health and Nutrition Examination Survey. Arch Pediatr Adolesc Med. 1998 Dec;152(12):1225-31.

51. Rebelo F, Carrilho TR, Canuto R, Schlussel MM, Farias DR, Ohuma EO, Kac G. Estimated fetal weight standards of the INTERGROWTH-21st project for the prediction of adverse outcomes: a systematic review with meta-analysis. J Matern Fetal Neonatal Med. 2023 Feb;36(2):2230510.

52. Kramer MS. Determinants of low birth weight: methodological assessment and meta-analysis. Bull World Health Organ. 1987;65(5):663-737.

This page contains no comments

53. Rasmussen KM, Yaktine AL, Institute of Medicine. Weight gain during pregnancy: Reexamining the guidelines. Washington, DC: The National Academies Press; 2009.

54. Xu J, Wang H, Bian J, et al. Association between the maternal Mediterranean diet and perinatal outcomes: a systematic review and meta-analysis. Adv Nutr. 2024;15:100159. doi:10.1016/j.advnut.2024.100159.

55. Haugen M, Brantsaeter AL, Trogstad L, et al. Infant birth weight and maternal diet in pregnancy: a Norwegian cohort study. Acta Obstet Gynecol Scand. 2008 May;87(5):532-40.

56. Knudsen VK, Orozova-Bekkevold IM, et al. Major dietary patterns in pregnancy and fetal growth. Eur J Clin Nutr. 2008 Apr;62(4):463-70. doi:10.1038/sj.ejcn.1602753.

57. Xu J, Wang H, Bian J, Xu M, Jiang N, Luo W, Zu P, Yin W, Zhu P. Association between the maternal Mediterranean diet and perinatal outcomes: a systematic review and meta-analysis. Adv Nutr. [Year missing];15. [Duplicate of 54? Please confirm.]

58. Díaz-López A, Díaz-Torres S, Martín-Luján F, Basora J, Arija V. Prenatal adherence to the Mediterranean diet decreases the risk of having a small for gestational age baby: The ECLIPSES study. Sci Rep. 2022 Sep 22;12(1):13794. doi:10.1038/s41598-022-18036-2.

59. Reijnders IF, et al. The impact of periconceptional maternal lifestyle on clinical features and biomarkers of placental development and function: a systematic review. Hum Reprod Update. 2019 Jan 1;25(1):72-94. doi:10.1093/humupd/dmy037.

60. Garcia-Montero C, Fraile-Martinez O, De Leon-Oiva D, et al. Exploring the role of Mediterranean and Westernized diets and their main nutrients in the modulation of oxidative stress in the placenta: a narrative review. Antioxidants (Basel). 2023 Oct 26;12(11):1918. doi:10.3390/antiox12111918.

61. Karbasi S, Mohamadian M, Naseri M, Khorasanchi A, Zarban A, Bahrami A, Ferns GA. A Mediterranean diet is associated with improved total antioxidant content of human breast milk and infant urine. Nutr J. 2023 Jan 19;22(1):11. doi:10.1186/s12937-023-00841-0.

62. Gesteiro E, Rodriguez Bernal B, Bastida S, et al. Maternal diets with low healthy eating index or Mediterranean diet adherence scores are associated with high cord blood insulin levels and insulin resistance markers at birth. Eur J Clin Nutr. 2012 Sep;66(9):1008-15. doi:10.1038/ejcn.2012.92.

63. Zaragoza-Martí A, Ruiz-Ródenas N, Herranz-Chofre I, Sánchez-SanSegundo M, Serrano Delgado VDLC, Hurtado-Sánchez JA. Adherence to the Mediterranean diet in pregnancy and its benefits on maternal-fetal health: a systematic review of the literature. Front Nutr. 2022 Jul 19;9:813942. doi:10.3389/fnut.2022.813942.

This page contains no comments

64. Assaf-Balut C, García de la Torre N, Durán A, Fuentes M, Bordiú E, Del Valle L, Familiar C, Ortolá A, Jiménez I, Herraiz MA, Izquierdo N. A Mediterranean diet with additional extra virgin olive oil and pistachios reduces the incidence of gestational diabetes mellitus (GDM): a randomized controlled trial: The St. Carlos GDM prevention study. PLoS One. 2017 Oct 19;12(10):e0185873.

65. Martínez-González MA, García-Arellano A, Toledo E, Salas-Salvado J, Buil-Cosiales P, Corella D, Covas MI, Schröder H, Aros F, Gomez-Gracia E, Fiol M. A 14-item Mediterranean diet assessment tool and obesity indexes among high-risk subjects: the PREDIMED trial. [Journal name and year missing—please provide.]

66. Almulla AA, Augustin H, Ahmed LA, Barebring L. Adherence to a Mediterranean-style dietary pattern during pregnancy in relation to gestational weight gain: The Mutaba'ah study. J Matern Fetal Neonatal Med. 2025;38(1):2496392. doi:10.1080/14767058.2023.2496392.

67. chón M, Úbeda N, García-González Á, Partearroyo T, Varela-Moreiras G. Effects of milk and dairy product consumption on pregnancy and lactation outcomes: A systematic review. Adv Nutr. 2019;10(Suppl_2):S74–87.

68. Olsen SF, Halldorsson TI, Willett WC, Knudsen VK, Gillman MW, Mikkelsen TB, et al. Milk consumption during pregnancy is associated with increased infant size at birth: prospective cohort study. Am J Clin Nutr. 2007;86(4):1104–10.

69. Olmedo-Requena R, Amezcua-Prieto C, et al. Association between low dairy intake during pregnancy and risk of small for gestational age infants. Matern Child Health J. 2016;20(6):1196–204.

70. Huang D, Wu Q, Xu X, et al. Maternal consumption of milk or dairy products during pregnancy and birth outcomes: A systematic review and dose-response meta-analysis. Front Nutr. 2022;9:900529.

71. Heppe DHM, van Dam RM, Willemsen SP, et al. Maternal milk consumption, fetal growth and the risks of neonatal complications: The Generation R Study. Am J Clin Nutr. 2011;94(2):501–9.

72. Clark DC. Dairy and growth, latest findings, and lessons learned. Food Nutr Bull. 2016;37(1_suppl):S22–8.

73. Meyer Z, Höflich C, Wirthgen E, Olm S, Hammon HM, Hoeflich A. Analysis of the IGF-system in milk from farm animals—Occurrence, regulation, and biomarker potential. Growth Horm IGF Res. 2017;35:1–7.

74. Wiley AS, Lubree HG, Joshi SM, et al. Pediatric obesity.

This page contains no comments

75. Xue H, Qin R, Xi Q, et al. Maternal dietary cholesterol and egg intake during pregnancy and large-for-gestational-age infants: A prospective cohort study. J Nutr. 2024;154(6):1880–9.

76. Díaz-López A, Bulló M, Martínez-González MA, et al. Mediterranean diet and its effect on birth weight outcomes: A systematic review. Am J Obstet Gynecol. 2022;226(1):53–60.

77. Reijnders IF, et al. The impact of periconceptional maternal lifestyle on clinical features and biomarkers of placental development and function: A systematic review. Hum Reprod Update. 2019;25(1):72–94.

78. Salajegheh Z, Nasiri M, Imanipour M, et al. Is oral consumption of dates (Phoenix dactylifera L. fruit) in the peripartum period effective and safe integrative care to facilitate childbirth and improve perinatal outcomes: A comprehensive revised systematic review and dose-response meta-analysis. BMC Pregnancy Childbirth. 2024;24(1):12.

79. El Rafei M, Fadlallah R, Khachfe H, et al. Total weight gain during pregnancy and its association with birth weight: A Lebanese cohort study. J Perinat Med. 2016;44(5):531–8.

80. Goldstein RF, Abell SK, Ranasinha S, et al. Association of gestational weight gain with maternal and infant outcomes: A systematic review and meta-analysis. JAMA. 2017;317(21):2207–25.

81. Black RE, Victora CG, Walker SP, et al. Maternal and child undernutrition and overweight in low-income and middle-income countries. Lancet. 2013;382(9890):427–51.

82. Goldstein RF, Abell SK, Ranasinha S, et al. Gestational weight gain across continents and ethnicity: systematic review and meta-analysis of maternal and infant outcomes in more than one million women. BMC Med. 2018;16:1–14.

83. Lowell H, Miller DC. Weight gain during pregnancy: adherence to Health Canada's guidelines. Health Rep. 2010;21(2):31.

84. Cheney K, Berkemeier S, Sim KA, et al. Prevalence and predictors of early gestational weight gain associated with obesity risk in a diverse Australian antenatal population: A cross-sectional study. BMC Pregnancy Childbirth. 2017;17:1–9.

85. Broskey N, Wang P, Lin Y, et al. Early pregnancy weight gain exerts the strongest effect on birth weight, posing a critical time to prevent childhood obesity. Obesity (Silver Spring). 2017;25(9):1569–76.

86. Sridhar SB, Xu F, Hedderson MM. Trimester-specific gestational weight gain and infant size for gestational age. PLoS One. 2016;11(7):e0159500.

This page contains no comments

87. Petersen JM, Hutcheon JA, Bodnar LM, et al. Weight gain patterns among pregnancies with obesity and small-and large-for-gestational-age births. Obesity. 2023;31(4):1133–45.

88. Sisti G, Fochesato C, Elkafrawi D, et al. Is blood pressure 120–139/80–89 mmHg before 20 weeks a risk factor for hypertensive disorders of pregnancy? A meta-analysis. Eur J Obstet Gynecol Reprod Biol. 2023;284:66–75.

89. de Haas S, Mulder E, Schartmann N, et al. Blood pressure adjustments throughout healthy and hypertensive pregnancy: A systematic review and meta-analysis. Pregnancy Hypertens. 2022;27:51–8.

90. JaddoeVW, Mackenbach JP, Moll HA, et al. The Generation R Study: design and cohort profile. Eur J Epidemiol. 2006;21:475–84.

91. Grewal J, Grantz KL, Zhang C, et al. Cohort profile: NICHD fetal growth studies–singletons and twins. Int J Epidemiol. 2018;47(1):25–31.

92. Iwama N, Kurosaki Y, Uchiyama S, et al. Maternal blood pressure and its association with fetal growth: A retrospective cohort study. Hypertens Pregnancy. 2016;35(3):268–76.

93. Elvan-Taşpınar A, Franx A, Bots ML, et al. Arterial stiffness and fetal growth in normotensive pregnancy. Am J Hypertens. 2005;18(3):337–41.

94. Cheung YF, Wong KY, Lam BC, et al. Relation of arterial stiffness with gestational age and birth weight. Arch Dis Child. 2004;89(3):217–21.

95. Galjaard S, Ameye L, Lees CC, et al. Sex differences in fetal growth and immediate birth outcomes in a low-risk Caucasian population. Biol Sex Differ. 2019;10(1):48.

96. Shah PS, Knowledge Synthesis Group on Determinants of LBW/PT births. Parity and low birth weight and preterm birth: a systematic review and meta-analyses. Acta Obstet Gynecol Scand. 2010;89(7):862–75.

97. Carlsen EM, Renault KM, Jensen RB, et al. The association between newborn regional body composition and cord blood concentrations of C-peptide and insulin-like growth factor I. PLoS One. 2015;10(7):e0121350.

98. Mohammadbeigi A, Farhadifar F, Zadeh NS, et al. Fetal macrosomia: risk factors, maternal, and perinatal outcome. Ann Med Health Sci Res. 2013;3(4):546–50.

99. Okun ML. Sleep disturbances and modulations in inflammation: implications for pregnancy health. Soc Personal Psychol Compass. 2019;13(5):e12451.

This page contains no comments

100. Facco FL, Grobman WA, Reid KJ, et al. Objectively measured short sleep duration and later sleep midpoint in pregnancy are associated with a higher risk of gestational diabetes. Am J Obstet Gynecol. 2017;217(4):447.e1–e6.

101. Reutrakul S, Van Cauter E. Sleep influences on obesity, insulin resistance, and risk of type 2 diabetes. Metabolism. 2018;84:56–66.

102. Howe LD, Signal TL, Paine SJ, et al. Self-reported sleep in late pregnancy in relation to birth size and fetal distress: the E Moe, Māmā prospective cohort study. BMJ Open. 2015;5(10):e008910.

Supporting information

S1 Table. Differences in maternal and infant risk Factors across SGA, AGA and LGA infants

S2 Table. Dietary characteristics of mothers delivering SGA, AGA and LGA infants

This page contains no comments

Click here to access/download
Supporting Information
Supporting information 1.docx

This page contains no comments

Click here to access/download
Supporting Information
Supporting information 2.docx

This page contains no comments
