# Supplementary material for: Predictors of infant birth weights: Role of the Lebanese mediterranean diet, psychosocial factors and maternal health status
Source: PLoS One. 2026 Jun 10;21(6):e0351497. doi: 10.1371/journal.pone.0351497 (PMC13252803; doi:10.1371/journal.pone.0351497)
Supplement: Attachment — Submitted filename: Response to Reviewers.docx [file pone.0351497.s003.docx]

**Response to Reviewers’ Comments**

We sincerely thank the Editor and Reviewers for their detailed and constructive feedback. We have carefully addressed each comment and revised the manuscript accordingly. Changes have been highlighted in the revised manuscript. Our responses are presented below in a point-by-point format.

**Editor and Major Comments**

**Abstract**

**Comment:** The abstract ends abruptly monitoring MAP, PP and sleep quality and promoting adherence to the LMeD. Please add a concluding sentence summarizing implications.

**Response:** We added a concluding sentence highlighting the study implications:

“Screening for family history of diabetes and macrosomia, targeting trimester-specific gestational weight gain, monitoring maternal blood pressure, pulse pressure, and sleep quality, and promoting adherence to the Lebanese Mediterranean diet are important strategies to optimize infant birth outcomes.”

**Introduction**

**Comment:** The manuscript is overly lengthy, with extensive detail that at times obscures the main findings. Recommendation: Streamline the Introduction and Discussion by eliminating redundancies and emphasizing the most relevant evidence.

**Response:** Introduction and Discussion were revised to remove redundancies, improve clarity, and emphasize literature most relevant to maternal diet, psychosocial factors, and infant birth outcome.

**Study Design and Sampling**

**Comment:** The description of the sampling strategy is insufficient and requires substantial clarification. The manuscript states that “Among 732 obstetric clinics, a total of 20 private and hospital‑based private clinics were selected using simple random sampling.” However, the process by which this random selection occurred is unclear. Please clarify the following points:
o How were the 20 clinics randomly selected? (Was a random number generator used? Or Were clinics stratified by governorate before random selection?)
o How were participants recruited within each clinic? (consecutively or randomly recruited?)
o Explain the apparent inconsistency in regional recruitment (30.7% of participants were recruited from Mount Lebanon, drawn from 3 clinics, while 12.4% were recruited from Beirut, drawn from 5 clinics, and that a minimum of 30 women were recruited from each clinic). These numbers are difficult to reconcile. If each clinic contributed approximately 30 participants, then regions with more clinics should contribute more participants. Instead, the opposite appears to be true.
o Given the unequal distribution of clinics and participants across governorates, weighting may be necessary to avoid over‑ or under‑representation of certain regions. If no weighting was applied, please justify this decision

**Response:**

- **Clinic Selection:** The 20 clinics were stratified by governorate. This was added to the study participants section line 124-126.
- **Participant Recruitment:** Participants were randomly recruited within each clinic at their first prenatal visit. This was added to the study participants section line 128.
- **Regional Distribution:** Clinics in Mount Lebanon recruited more women per clinic to achieve target sample, explaining apparent discrepancies
- **Weighting:** No post-stratification weighting was applied; governorate of residence was included as an adjustment variable in analyses to account for regional differences.

**Comment:** Line 128 refers to “South China” and “North China.”

**Response:** Corrected to South Lebanon and North Lebanon in line 126-127.

**Study Design**

**Comment:** The manuscript states that “the questionnaires were re-administered in the 2nd and 3rd trimesters”, yet Table 2 presents data for certain instruments (e.g., Pittsburgh Sleep Quality Index and Perceived Stress Scale) across T1, T2, and T3, implying administration in the first trimester as well. In contrast, PARmed-X for Pregnancy is only reported for T2 and T3. This inconsistency suggests a reporting bias

**Response:** Clarified in the Methods lines 149-150 that the PSS-10, PSQI and PARmed-X were administered at all three trimesters. Table 2 was updated to reflect this.

**Comment:** Abbreviations need to be spelled out at first mention (SBP, DBP, MAP, PP, LMIC).

**Response:** All abbreviations have been defined at first mention in the revised manuscript.

**Comment:** Line 176-177:” this tool had a good correlation (r=0.56) with the Italian MeD tool.” Saying "good correlation" for r = 0.56 is a bit subjective. In healthcare research, a correlation coefficient of r ≈ 0.5–0.6 is generally interpreted as moderate, not “good.”, you might want to phrase it more precisely: “This tool demonstrated a moderate positive correlation (r=0.56) with the Italian MeD tool”. and consider moving this statement to the first paragraph of the “Dietary assessment and adherence to the Lebanese Mediterranean diet” section to enhance readability and emphasize its importance

**Response:** Changed to:

“This tool demonstrated a moderate positive correlation (r=0.56) with the Italian MeD tool” and moved to the first paragraph of the Dietary Assessment section Lines 164-165.

**Comment:** Line 171-172: The authors reported “in the LMeD, other main components, such as fish, red meat, poultry, and wine consumption, are missing. While this adaptation reflects cultural dietary practices, the absence of these elements may limit comparability with other Mediterranean populations and should be acknowledged as a methodological limitation

**Response:** This has been added in the limitations section lines 538-540.

**Comment:** Line 171 notes wine consumption as missing, while Line 177 explains that alcohol intake (including wine) was excluded due to cultural absence. This overlap creates redundancy and potential confusion regarding whether wine is missing due to cultural adaptation or excluded as part of alcohol intake. Consider merging these into a single, consistent explanation to improve clarity.

**Response:** The sentence about alcohol intake has been deleted to prevent confusion line 178. It has been excluded in the original validation by Naja et al. 2015 as part of cultural absence. We mentioned that these elements did not load high on the pattern the LMeD lines 174-175.

**Comment:** The LMeD score is based on tertiles of intake, but the manuscript does not specify whether tertiles were calculated per trimester or based on baseline distribution.

**Response:** LMeD tertiles were calculated per trimester. This has been clarified in Methods lines 178.

**Comment:** The text states that FBG values were collected only in trimesters 1 and 3, yet categorization is described as occurring during the second trimester visit. This creates confusion: how can categorization be based on a trimester where no values were collected?
o The unit “mmol/” is incomplete; it should be “mmol/L. line 201

**Response:** There has been a misplacement of the punctuation mark. We have corrected this section to indicate that FBG were collected in trimesters 1 and 3, while GDM diagnosis was made in trimester 2 based on the two-step OGTT test, and the unit has been corrected to mmol/L line 204.

**Comment:** Line 214: Infant birth outcomes: Clarify whether gestational age was based on LMP or ultrasound. Line 208-212: The use of trimester-specific MAP cutoffs is appropriate; however, the rationale for applying PP cutoffs derived from a single population study with only 20 women (reference 49) requires justification. Line 208-212: Given that the study population was drawn from multiple clinics across different geographic areas, the authors should specify whether blood pressure measurements were obtained using standardized protocols and uniform devices. This information is essential to ensure consistency, reliability, and comparability of measurements across sites. If standardized procedures or devices were not used, this should be explicitly acknowledged and discussed as a limitation of the study.

**Response:** Gestational age was based on last menstrual period (LMP) confirmed by first-trimester ultrasound. This sentence has been added to the methods section Line 224-225.

As for pulse pressure, previous studies have defined these categories based on the distribution of measurements or means within their respective cohorts. In a recent study by Sampson et al., 2024, they chose to use the dichotomous cutoff of PP > 55 mmHg based on previous studies (Mullan et al., 2021; Thadhani et al., 2001; Maykin et al., 2024). The study that we cited before was the most recent one at the time. Reference 49 has been updated to reflect the new study by Sampson et al. 2024 lines 214-216. Blood pressure measurements were obtained using standardized protocols. A sentence in the limitations was added that the devices used by gynecologists were not uniform line 543-544.

References below:

Reference: Mullan S.J., Vricella L.K., Edwards A.M., Powel J.E., Ong S.K., Li X., Tomlinson T.M. Pulse pressure as a predictor of response to treatment for severe hypertension in pregnancy. Am. J. Obstet. Gynecol. MFM. 2021;3:100455. doi: 10.1016/j.ajogmf.2021.100455. [[DOI](https://doi.org/10.1016/j.ajogmf.2021.100455)] [[PubMed](https://pubmed.ncbi.nlm.nih.gov/34375751/)] [[Google Scholar](https://scholar.google.com/scholar_lookup?journal=Am.%20J.%20Obstet.%20Gynecol.%20MFM&title=Pulse%20pressure%20as%20a%20predictor%20of%20response%20to%20treatment%20for%20severe%20hypertension%20in%20pregnancy&author=S.J.%20Mullan&author=L.K.%20Vricella&author=A.M.%20Edwards&author=J.E.%20Powel&author=S.K.%20Ong&volume=3&publication_year=2021&pages=100455&pmid=34375751&doi=10.1016/j.ajogmf.2021.100455&)][[Ref list](https://pmc.ncbi.nlm.nih.gov/articles/PMC11312723/#B11-jcm-13-04318)]

Thadhani R., Ecker J.L., Kettyle E., Sandler L., Frigoletto F.D., Jr. Pulse pressure and risk of preeclampsia: A prospective study. Obstet. Gynecol. 2001;97:515–520. doi: 10.1016/s0029-7844(00)01192-3. [[DOI](https://doi.org/10.1016/s0029-7844(00)01192-3)] [[PubMed](https://pubmed.ncbi.nlm.nih.gov/11275020/)] [[Google Scholar](https://scholar.google.com/scholar_lookup?journal=Obstet.%20Gynecol.&title=Pulse%20pressure%20and%20risk%20of%20preeclampsia:%20A%20prospective%20study&author=R.%20Thadhani&author=J.L.%20Ecker&author=E.%20Kettyle&author=L.%20Sandler&author=F.D.%20Frigoletto&volume=97&publication_year=2001&pages=515-520&pmid=11275020&doi=10.1016/s0029-7844(00)01192-3&)][[Ref list](https://pmc.ncbi.nlm.nih.gov/articles/PMC11312723/#B17-jcm-13-04318)]

Maykin M.M., Mercer E., Saiki K.M., Kaneshiro B., Miller C.B., Tsai P.-J.S. Furosemide to lower antenatal severe hypertension: A randomized placebo-controlled trial. Am. J. Obstet. Gynecol. MFM. 2024;6:101348. doi: 10.1016/j.ajogmf.2024.101348.

Sampson R, Davis S, Wong R, Baranco N, Silverman RK. Pulse Pressure as a Hemodynamic Parameter in Preeclampsia with Severe Features Accompanied by Fetal Growth Restriction. J Clin Med. 2024 Jul 24;13(15):4318. doi: 10.3390/jcm13154318. PMID: 39124585; PMCID: PMC11312723.

**Comment:**  Psychosocial variables (line 220-226)
o In Lines 220–226, the authors claim that the Perceived Stress Scale (PSS-10) has been validated among the Lebanese population, but no supporting reference is provided. The appropriate citation is: Chaaya, M., Osman, H., Naassan, G., et al. (2010). Validation of the Arabic version of the Cohen Perceived Stress Scale (PSS-10) among pregnant and postpartum women. BMC Psychiatry, 10, 111. https://doi.org/10.1186/1471-244X-10-111. In this validation study, Cronbach’s alpha was used to assess internal consistency reliability, while test–retest reliability was evaluated using Spearman’s correlation coefficient. These two values should be reported separately to clearly distinguish between the two scales.
o Line 223-224: The author reported that Lebanese version is Correlated with and with a general health questionnaire (r=0.48) and EPDS (r=0.58), but was not clear from where these value came, the original article in table-2 (Chaaya 2010) reported different values (.59) and .49 respectively.”

**Response:** We thank the reviewer for pointing out the missing reference and clarifying the reliability and validity values. We have updated the manuscript to correctly cite Chaaya et al. (2010) and report Cronbach’s alpha (0.74) and test–retest reliability (Spearman’s r = 0.49) separately. Correlations with the General Health Questionnaire (r = 0.59) and EPDS (r = 0.49) have also been corrected to reflect the original validation study lines 231-235.

**Comment:** Lines 228–232: The study validating the Arabic version of the Pittsburgh Sleep Quality Index (PSQI) is not cited. The appropriate reference is: Suleiman K., Al-Hadid L., Duhni A. (2012). Psychometric testing of the Arabic version of the Pittsburgh Sleep Quality Index (A‑PSQI) among coronary artery disease patients in Jordan. Journal of Natural Sciences Research, 2(8), 15–20

**Response:** We have updated the reference for the validation study line 243.

o Line 236: The term “global sum” is ambiguous. Do you mean the “total score”? Please clarify the terminology.

**Response:** This has been corrected line 240.

o Line 237-239: The authors reported that Edinsburgh Perinatal/Postnatal Depression Scale (EPDS) tool was used to assess depression, however, the reference cite the tool Edinsburgh Postnatal Depression Scale (EPDS) 1987 which used only for POST natal. Furthermore, the authors claim that the tool has been validated among the Lebanese population, but no supporting reference is provided. The appropriate citation is “Ghubash, R., Abou-Saleh, M. T., & Daradkeh, T. K. (1997). The validity of the Arabic Edinburgh Postnatal Depression Scale. Social Psychiatry and Psychiatric Epidemiology, 32(8), 474–476. <https://doi.org/10.1007/BF00789142>”

Response: The name of the tool was corrected to Edinsburgh Postnatal Depression Scale (EPDS) tool line 246. The citation for validation has been updated line 249. The word Lebanese has been replaced with Arabic population line 248.

**Results**

**Comment:** Results simply restate tables; sample size inconsistencies (618 vs 576).

**Response:**

- Narratives were rewritten to interpret findings, emphasizing trends and group differences.
- Sample size inconsistencies were clarified: 618 women is the exact number of pregnant women who completed the study. The 42 participants dropped from the initial sample size of N=660. This has been added to the methods section to clarify and removed from the table line 158-159.

**Comment:** Model 1 referenced but not presented; non-significant regression results omitted.

**Response:** Full regression tables, including Model 1 and non-significant variable values, have been added to **Supplementary Tables S1–S3** for transparency line 1019-1025.

**Comment:** Tables restructured for clarity (percentages for categorical variables, moved long variables to Supplementary Tables).

**Response:** Tables revised per reviewer recommendations.

**Comment:** Education skew discussed as a limitation.

**Response:** We added in the limitations the following statement “Limitations include reliance on self-reported data, a highly educated sample (76%) which may skew results and limit generalizability” line 536.

**Comment:** Stress and depression not included in models; discuss prevalence >50%.

**Response:** Explanation that these variables were not significant in multivariable models has been explained in the results line 325, discussion line 517, and limitations lines 537.

**Comment:** Dairy association with BWGA corrected (T2, not T3).

**Response:** Table 3 and Results text updated accordingly line 359.

**Discussion**

**Comment:** The Discussion section is overly narrative and includes tangential literature.
The discussion section is relatively long, and several paragraphs repeat or restate results rather than providing interpretation. For example, the paragraph in lines 577–585 (“Pre‑pregnancy BMI, gestational weight gain (GWG) and birth outcomes”) reads more like a results summary than a critical discussion. In addition, multiple sections are overly lengthy and contain redundancies. Streamlining the discussion, removing repeated results, and focusing on interpretation, implications, and comparison is needed. The authors used sometimes overstates causality, therefore, using cautious language such as “associated with,” “may contribute to” is highly recommended.

**Response:** We have streamlined discussion to emphasize interpretation, implications, and comparison with literature. Language revised to **“associated with”** rather than implying causality throughout the discussion.

**Comment:** Lines 533–537: The Iranian study you cite does not support your findings as stated. The Iranian study reports that as the ratio of MUFA (olive oil) to SFA increases, gestational weight gain decreases—meaning higher olive intake is associated with lower GWG. In contrast, your results indicate that women who delivered LGA infants had higher olive intake in Trimester 2. These two interpretations appear contradictory. Please clarify this discrepancy and explain how the cited study supports your findings. You may need to discuss potential confounding such as total energy intake, cooking methods.

**Response:** A statement about the potential confounding (energy intake, cooking methods) has been added to the limitations lines 540-541

The reference has been amended in the text line 482 and in the reference list “66”.

**Comment:** Line 495: The author reported that LMeD adherence did not enter models for SGA or LGA, yet specific LMeD components did. This distinction needs clearer framing in the discussion: why does the overall pattern fail to predict risk while individual components like olive oil in T2 increase LGA risk?

**Response:** LMeD is associated with AGA. I added explanation that individual dietary components may have distinct associations than composite scores due to specific nutrient effects Line 490-492.

**Comment:** Line 498-499: Is there any explanation why the AGA mothers consistently show better adherence to the LMeD in later trimesters.

**Response:** A sentence has been added to the discussion lines 466-470 to explain this.

**Comment:** Line 555: Eggs show the highest positive coefficient among the listed food groups. It is recommended to explicitly link this finding to its nutritional significance. Eggs are a high‑quality protein source and provide key nutrients such as choline and essential fatty acids, which play critical roles in fetal brain development, cellular growth, and overall pregnancy outcomes. Highlighting these mechanisms would strengthen the interpretation of this result.

**Response:** Added text:

“Eggs provide high-quality protein, choline, and essential fatty acids, which contribute to fetal growth and brain development. Line 485-486.

**Comment:** Line 696: The authors describe the association between poor sleep in T3 and increased LGA risk as a “novel psychosocial dimension.” I disagree with this characterization, as multiple previous studies have already reported similar associations between maternal sleep disturbances and fetal overgrowth. The authors should revise this statement to accurately reflect the existing body of evidence

**Response:** Revised to: “Poor sleep quality in T3 was associated with higher LGA odds, consistent with prior evidence linking maternal sleep with elevated cortisol, as well as altered glucose metabolism and insulin regulation increasing the risk of LGA line 515-517.

**Other Comments**

**Comment:** The authors state that the data are available within the manuscript and supporting files. However, PLOS ONE requires that the minimal data set be provided in a form that allows full replication of the analyses. In the current submission, non‑significant results appear to be omitted—even from the supplementary materials. Without access to the complete regression outputs, including non‑significant predictors, it is not possible to adequately review or interpret the models.
A major concern relates to the reference list, as a substantial number of the cited sources are not retrievable using the information provided in the manuscript. This issue significantly undermines the credibility of the work. Ensure all references are current and correctly formatted and are complete (some missing journal names or years). Reference 57 appears to be a duplicate of Reference 54.

**Response:** Full dataset, including non-significant predictors, is now provided in the manuscript and in supplementary Files.

Reference list has been carefully reviewed, corrected, and duplicates removed; all author names included according to journal guidelines.

Abstract :
Some editing mistakes (L41 « . ») / review the conclusion (nonverbal phrase).

**Response:** Done line 44-47

Introduction :
L108 : abbreviation of Body mass Index (BMI)

**Response:** Done line 101

Materials and methods:
L114 : how did u define the study being « national » ?, while the recrutment was exclusively conducted in private clinics and not public hospitals and the recrutment from the capital Beirut was just 12.4%.

**Response**: Recruitment was done through private and public clinics, and participants were enrolled proportionally from different governorates based on population distribution in Lebanon. Mount Lebanon, being the most densely populated region, contributed the largest share of participants, while Beirut represented 12.4% of the sample. Therefore, the study still reflects a national-level representation in terms of geographic distribution.” Line 111-113 and lines 124-126. However, a statement was added in the limitations section to highlight that our findings do not reflect the true national distribution, as variations in response rates and sample composition could limit representativeness. (line 533-535)

L119 : If women were recruited in their 1st trimester of gestation, are the structural and chromosomal anomalies diagnosted at this early stage of pregnancy ?

**Response:** Such cases were not present, however we originally intended to exclude them once a diagnosis was made.

L126 : how did you ended up with the 732 obstetric clinics ? was the data from the ministry of health ? or other organisation ?

**Response:** The data was obtained from the syndicate of physicians in Lebanon and this statement was added in line 124.

L128 : The study is conducted in Lebanon and not in South or North China like mentionned.

Response: This has been corrected line 126

L133 : Provide the ethical consent of the affiliated academic institution.

**Response:** The ethical approval was mentioned in the methods section line 130-131.

L149 : Provide more information about the physical activity status of participating pregnant women. Why the research team did not rely on a validated tool such as the PPAQ ?

**Response:** The PPAQ is very long, and we preferred to use a short tool to assess physical activity as it was not our main outcome. Most women were sedentary (>84%) as mentioned in table 2 and it was not included in the regression models.

L153 : why anemia was assessed ? and not explored later in regard to neonatal outcomes ? were the participants supplemented ?

Anemia was assessed and the result was 25%, however we did not include it in the analyses as it was not our main variable of interest. A sentence was added line 294.

**Response:**
L160/161 : Pls note that this FFQ was not validated among pregnant Lebanese women. This should be cited in the limitations.

**Response:** This has been added to the limitations line 538.

L168 : dried fruits or nuts ? since oleaginous fruits are more consumed by the Lebanese population.

**Response:** The LMeD tool found that dried fruits alone consistently loaded high on this pattern and therefore was included as a main element of this tool line 171.

L171 : fish, red meat and wine are major food components implicated in measuring the adherence in the Mediterranean diet. Provide an explanation why they were excluded from the initial tool, since it may affect the results and not considered a reliable tool?

**Response:** Fish, red meat and wine are not highly consumed by the Lebanese population and did not load high on the pattern in contrast to other elements of the LMeD such as burghul, fruits, dairy products, legumes, dried fruits, eggs, and others that loaded high on the patter, this was highlighted in line 175. We also mentioned this as a limitation line 539.

L173 : Provide a detailed measurement analysis of this tool. How was the final adherence to Med Diet among this sample? Compare and cite with studies assessing dietary patterns among Lebanese pregnant women.

**Response:** The following sentence has been added: Adherence to the LMeD was significantly different between trimester 1, 2 and 3. In trimester 3, a higher percentage of women (26.0%) had low adherence scores compared to trimester 1 and 2 (19.0% and 24.0% respectively) (p<0.001), and in trimester 2 more women were identified in the high adherence group (30.0%) compared to trimester 1 (21.0%) and trimester 3 (25.0%) Line 298-302.

In response to the reviewer’s comment, we would like to clarify that dietary patterns among Lebanese pregnant women have already been contextualized through the citation of Naja et al. 2015 which represents the most recent and comprehensive assessment of dietary patterns in this population. This study specifically examined dietary pattern adherence during pregnancy in Lebanon and therefore closely aligns with the objectives of the present work. Other recent Lebanese studies were not cited due to differences in scope and focus. For instance, the study by Papazian et al. did not identify significant associations between dietary patterns and maternal or infant outcomes, which was beyond the focus of the present analysis. Additionally, the study by Mahfouz et al. primarily assessed macronutrient, micronutrient, and energy intake among pregnant women rather than dietary patterns or Mediterranean diet adherence. While these studies provide important insights into maternal nutrition in Lebanon, they were not directly comparable to the aims or methodology of the current study. Collectively, these represent the most recent research conducted among pregnant women in Lebanon and support the relevance of the present findings.

Papazian T, Salameh P, Abi Tayeh G, Kesrouani A, Aoun C, Abou Diwan M, Rabbaa Khabbaz L. Dietary patterns and birth outcomes of healthy Lebanese pregnant women. *Frontiers in Nutrition*. 2022;9:977288. doi:10.3389/fnut.2022.977288.

Mahfouz R, Akiki MT, Ndayra V, El Khoury R, Chawi M, Hatem M, Hanna-Wakim L, Sacre Y, Hoteit M. Energy, Macronutrients and Micronutrients Intake Among Pregnant Women in Lebanon: Findings from the Updated Lebanese National Food Consumption Survey (LEBANON-FCS). *Nutrients*. 2024 Nov 26;16(23):4059. doi: 10.3390/nu16234059. PMID: 39683453; PMCID: PMC11643267.

L177 : Reference 35 is Buysse DJ, et al. Pittsburgh Sleep Quality Index (PSQI). Sleep. 1989;12(1):65-72 and not an italian validated Med tool !

**Response:** This is a typing mistake. We have corrected the reference 35 to 39 in the text line 165 and in the reference list.

L188 : change the word « according » written twice in the same phrase.

**Response:** This has been corrected line 191.

L204 : is it 50 g or 75 g or 1g / kg body weight ? (75g is more widely used)

**Response:** We did a survey among the obstetricians participating in the study and they all used the 50 g oral glucose tolerance test. This was added in the methods section line 206-207.

L214 : cite the other birth outcomes, since they were mentioned in the result part.

**Response:** Done line 220 and 224 where we included birth weight, gestational age and infant sex as birth outcomes.

L250 : how was the physical activity assessed and categorized ?

**Response:** This has been explained in the methods section line 148-149.

L280 : pls correct all values ( 63.4% instead of 67.6% , 32.7% instead of 32.4% and change « or » with « and » )

**Response:** Done line 290.

L293 : Add N= … the number of participants in the title / L336 too
Table 1 Some editing mistakes « , » near GWG T3 and remove « . » near 58 (Boy) /

**Response:** Done line 304

L315 : Delivery mode was not cited in the results. Why this variable was not taken into account during the analyses?
**Response:** We did collect data on delivery mode; however, it was not included in the analyses as the number of study variables was already extensive and the inclusion of additional variables would have increased model complexity without directly addressing the primary objectives of the study. Future analyses may explore the role of delivery mode in greater detail.

L316 Participants suffering from delivery complications, were they dropped from the study ? Data is not reported in the tables.

**Response:** They were not dropped from the study. The prevalence was 12.3%. A sentence has been added line 293-294 and table 1 was updated accordingly.
L340 : the format of the footer in table 1 is different from table 2, 3… pls review the formatting
**Response:** The format of the footer was unified across all tables.

Discussion :
- No mention about the smoking status of the participants, especially that it may be linked to negative neonatal outcomes and linked to HBP.

**Response:** The following sentence has been added: Less than 4% smoked in T1, and less than 2% in T2 and less than 1.1% in T3 line 289-290.

- L503 editing mistake « . » after ref 58.

**Response:** Done

- L536 : higher olive intake ? or olive oil ? and specifically how much in ml ?

**Response:** The association refers specifically to higher olive oil intake. Olive oil consumption was entered into the logistic regression model as a continuous variable (servings/day or 1 tablespoon/day); therefore, the reported odds ratio represents the change in risk associated with each additional tablespoon per day. Because intake was modeled continuously, the effect is incremental rather than categorical, and no specific volume (ml) can be inferred. Receiver operating characteristic (ROC) curve analysis was conducted to explore the presence of a potential threshold or cut-off value for olive oil intake; however, no discriminatory cut-off was identified, as reflected by the absence of a meaningful AUC.

- L559 : fruits or nuts ?

**Response:** dried fruits line 486

- L571 – 573 : the statement proposed is not clear. Pls rephrase.

**Response:** done line 551-553

- L577 : replace reference 79, by recent published studies conducted among Lebanese pregnant women in regard to neonatal outcomes.

**Response:** Done line 498

- L707 editing mistake « . » after « findings ».
**Response:** Done


Limitations :
L713 Mention about the limitations of the tool used to assess the Med-diet adherence and the lack of assessment of not only energy intake but also macro and micronutrient and fiber content of their diet, since neonatal outcomes are highly influenced by caloric intake and the contribution of fat and protein during pregnancy.

**Response:** A sentence has been added to the limitations section lines 540-542


References :
Review pls the referencing of some citations too (57, 74..). Provide the names of all authors, unless if the publication is authored by more than 6 authors and not just the first author with al. The name of some contributing authors are lacking in the citations.

**Response:** Done

Reviewer #2: The authors are commended for conducting such a robust study in a population where there is a paucity of data related to pregnancy outcomes.
However, there are several concerns about some of the unusual finding from this study such as
associations of healthy fat intake (olive oil) and wheat with poor pregnancy outcomes and (LGA and SGA respectively), high MAP during pregnancy being associated with LGA and poor sleep being associated with LGA. The authors are encouraged to robustly explain these findings and how they contradict current understandings in this field.

**Response:** Thank you for your comment. Actually, we mentioned in the discussion that olive oil intake is associated with higher birth weight which may be due to higher intake of healthy fats, and we compared it with another study that also showed a decrease in SGA risk line 479-482.

Moreover higher MAP was associated with SGA which is in line with previous studies that reported an association between hypertension during pregnancy and impaired fetal growth line 504-506.

With regards to sleep and LGA, we explained that this observation may be due to elevated cortisol, altered glucose metabolism, and insulin resistance leading to LGA infants line 514-517.
