# Supplementary material for: Predictors of infant birth weights: Role of the Lebanese mediterranean diet, psychosocial factors and maternal health status
Source: PLoS One. 2026 Jun 10;21(6):e0351497. doi: 10.1371/journal.pone.0351497 (PMC13252803; doi:10.1371/journal.pone.0351497)
Supplement: Attachment — Submitted filename: Point by Point Response.docx [file pone.0351497.s004.docx]

Point by Point Response

• Manuscript length and focus: I still believe that the manuscript is overly lengthy, with extensive detail that at times obscures the main findings. Further streamlining of the Introduction and Discussion is needed, particularly by removing redundancies and focusing on the key messages.

Response: Thank you for your response. Both introduction and discussion have been streamlined to focus on the key messages and remove redundancies.

• Line 125 – Clarification of clinic types: “A total of 20 private and hospital-based private clinics were stratified by governorate.” Please clarify the distinction between private clinics and hospital-based private clinics. How do these settings differ operationally or structurally, and why is this distinction relevant to the study?

Response: To our knowledge, there are no publicly available national data in Lebanon that quantify the number of obstetric clinics by type (private vs hospital-based private). This is partly due to the absence of a centralized registry that classifies outpatient facilities according to their structural affiliation.

However, available evidence suggests that obstetric care in Lebanon is predominantly delivered in the private sector. For example, a national study reported that approximately 64.9% of obstetricians primarily practice in private clinics, while 21.6% practice in hospital-based private clinics (El Khoury et al., 2024).

Given this distribution and the overlap in practice settings among physicians, our study stratified clinics by type to ensure representation of both independent and hospital-affiliated outpatient care environments rather than to reflect exact national counts.

We have the added the following sentence in lines 111-112: The sample was proportionally allocated to include 15 private clinics and 5 hospital-based private clinics

Reference: El Khoury T, Rebeiz MC, Abi Zeid B, Mansour S, Yared G, El Kak F, Akik C, McCall SJ. An assessment of the content of antenatal care provided by obstetricians in Lebanon: A cross-sectional study. PLOS Glob Public Health. 2024 Nov 4;4(11):e0003853. doi: 10.1371/journal.pgph.0003853. PMID: 39495764; PMCID: PMC11534237.

• Line 128 – Definition of “first prenatal visit” and recruitment strategy
“Participants were then randomly recruited within each clinic at their first prenatal visit.”
Please clarify what is meant by “their first prenatal visit.” Does this refer to women attending their first-ever prenatal visit at that clinic during the study period? If so, this appears closer to consecutive recruitment rather than random recruitment.

Response: Thank you for your comment. I have adjusted lines 115-118 to the following: Participants were consecutively recruited within each clinic at their first prenatal visit during the study period. This refers to women presenting for their initial antenatal consultation for the current pregnancy, all of whom were in their first trimester (<12 weeks of gestation) at the time of enrollment.

• Lines 112–113 – Governorate distribution and percentages: “Governorates of Lebanon that included Mount Lebanon (30.7%), Beirut (12.4%), Bekaa (16.4%), South Lebanon (and Nabatieh) (29.4%), and Akkar (3.7%).”
The percentages sum to 92.6%, not 100%.

Response: Thank you for your response. There was a typing mistake and we have edited the sentence to include the 6^th^ governorate which is North Lebanon (7.4%) line 99.

o Please provide the total number of participants per governorate in addition to percentages.
o Ensure that these numbers and percentages are consistent with what is reported in lines 126–127 and elsewhere in the manuscript.

Response: We have added next to the percentage the total number of participants for each governorate Lines 112-115.

• Pulse pressure cutoff and supporting literature
I recommend citing additional studies with larger populations and clearly defined pulse pressure (PP) thresholds to justify the cutoff used in this study. the rationale for the PP cutoff adopted in this study is not sufficiently supported by the literature and should be more clearly justified, For example:
o Mullan et al., 2021: PP cutoff 55 mmHg
o Thadhani et al., 2001: PP cutoff 50 mmHg
o Maykin et al., 2024: furosemide RCT that did not define or analyze a PP threshold.
o Sampson et al., 2024: PP cutoff 50 mmHg

Response: We thank the reviewer for this important comment. We would like to clarify that, in our study, pulse pressure (PP) was **not categorized using a predefined cutoff.** Instead, PP was analyzed as a **continuous variable** in all statistical models.

While several studies have proposed thresholds (e.g., 50–55 mmHg), these cutoffs are not consistently defined across the literature and vary depending on study population and clinical context. For instance, prior research has highlighted that there is **no universally accepted definition of “elevated” pulse pressure in pregnancy**, and thresholds are often derived empirically within individual cohorts rather than standardized across studies.

Given this variability and lack of consensus, we opted to retain PP as a continuous variable to preserve statistical power and avoid arbitrary categorization. This approach is methodologically robust and allows for a more precise assessment of the association between PP and the outcomes of interest.

I have updated the methods section to reflect the above justification lines 201-208.

In its current form,
• Lines 158–159: “Initially, 660 participants were recruited; however, 42 dropped out due to miscarriage or unwillingness to participate, making the total sample size N = 618.”
This statement is more appropriate for the Results section rather than the Methods. Please relocate this description of participant flow to the Results

Response: Thank you for your comment. We have relocated this sentence to the results section line 276-277.

• Table 1 –It would be preferable to display the units in parentheses (e.g., kg, %) and to standardize unit presentation across all variables in Table 1.

Response: The table has been updated to reflect units in parentheses

• Supplementary tables : Please ensure that the numbering and labeling of all supplementary tables are consistent between the text and the supplementary material.

Response: All supplementary tables have been cross-checked with the text, We have fixed the naming of the tables to ensure consistency.

• Table 3 –dairy products are reported as significantly associated with increased BWGA. Please verify whether this association pertains to T1 or T2 and ensure that the text and table are fully consistent and clearly labeled.

Response: The text has been updated to reflect associations of dairy products within the tables in both T1 and T2 for BWGA lines 353-354., while the association of dairy products with AGA remains only at T1 lines 361-362.

• Reference list: A major concern remains regarding the reference list. The references need to be revised carefully for accuracy, and consistency. Specific issues include, but are not limited to:
o The reference for Martínez-Galiano et al. (“Effect of Adherence to a Mediterranean Diet and Olive Oil Intake during Pregnancy on Risk of Small for Gestational Age Infants”) appears incomplete and/or incorrectly formatted. Please provide full citation details and numbering.

Response: I rechecked the reference of Martinez-Galiano and it has been correctly cited. I have also edited the reference list to make sure there are consistencies among the text and reference list.

o Line 235: The cited article number “34” appears to be incorrect; please verify and correct.

Response: The reference has been corrected

o Line 470: The reference cited here should be checked for accuracy and consistency with the reference list.

Response: Thank you. References 55-58 are accurate and consistent with the information in line 459.

o Lines 485–486: The reference cited to support the authors’ claim in these lines appears inappropriate or not directly supportive of the statement. Please revise the citation or modify the claim to align with the evidence.

Response: Reference 74 has been removed to align with the claim and the remaining references were re-arranged to follow reference 73.
