# Supplementary material for: Predictors of infant birth weights: Role of the Lebanese mediterranean diet, psychosocial factors and maternal health status
Source: PLoS One. 2026 Jun 10;21(6):e0351497. doi: 10.1371/journal.pone.0351497 (PMC13252803; doi:10.1371/journal.pone.0351497)
Supplement: Attachment — Submitted filename: Point_by_Point_Response_auresp_3.docx [file pone.0351497.s005.docx]

Dear Reviewer,

Thank you for your careful review and constructive comments. We have thoroughly revised the manuscript to address all concerns related to references and citation consistency.

Specifically:

- Reference 1 was updated.
- References 4 which was previously listed but not cited in the manuscript, has now been appropriately integrated into the text.
- Reference 54 has been integrated in the text and the new reference number is 44.
- Reference 81 was carefully verified and corrected for numbering, formatting, and citation placement and became number 56.
- We substantially reduced the number of citations supporting individual claims throughout the manuscript. In most cases, statements are now supported by one to two high-quality, recent, and directly relevant references, as recommended.
- The overall reference list was streamlined and reduced to a total of 66 references by removing redundant or marginally relevant citations.
- The crossed-out statement previously appearing at line 168 has been removed entirely from the revised manuscript.

We appreciate the reviewer’s valuable feedback, which helped improve the clarity, precision, and overall quality of the manuscript.
