# Supplementary material for: Predictors of infant birth weights: Role of the Lebanese mediterranean diet, psychosocial factors and maternal health status
Source: PLoS One. 2026 Jun 10;21(6):e0351497. doi: 10.1371/journal.pone.0351497 (PMC13252803; doi:10.1371/journal.pone.0351497)
Supplement: Peer review history

# Peer review: Decision Letter 0

**DOI:** 10.1371/journal.pone.0351497.r001

**Authors:** Maher Abdelraheim Titi

28 Dec 2025

PONE-D-25-31196

Predictors of Infant Birth Weights: Role of the Lebanese Mediterranean Diet, Psychosocial Factors and Maternal Health Status

PLOS One

Dear Dr. Fares,

Thank you for submitting your manuscript to PLOS ONE. After careful consideration, we feel that it has merit but does not fully meet PLOS ONE’s publication criteria as it currently stands. Therefore, we invite you to submit a revised version of the manuscript that addresses the points raised during the review process.

Please submit your revised manuscript by Feb 11 2026 11:59PM. If you will need more time than this to complete your revisions, please reply to this message or contact the journal office at plosone@plos.org. When you're ready to submit your revision, log on to https://www.editorialmanager.com/pone/ and select the 'Submissions Needing Revision' folder to locate your manuscript file.

Please include the following items when submitting your revised manuscript:

- A letter that responds to each point raised by the academic editor and reviewer(s). You should upload this letter as a separate file labeled 'Response to Reviewers'.
- A marked-up copy of your manuscript that highlights changes made to the original version. You should upload this as a separate file labeled 'Revised Manuscript with Track Changes'.
- An unmarked version of your revised paper without tracked changes. You should upload this as a separate file labeled 'Manuscript'.

If you would like to make changes to your financial disclosure, please include your updated statement in your cover letter. Guidelines for resubmitting your figure files are available below the reviewer comments at the end of this letter.

If applicable, we recommend that you deposit your laboratory protocols in protocols.io to enhance the reproducibility of your results. Protocols.io assigns your protocol its own identifier (DOI) so that it can be cited independently in the future. For instructions see: https://journals.plos.org/plosone/s/submission-guidelines#loc-laboratory-protocols. Additionally, PLOS ONE offers an option for publishing peer-reviewed Lab Protocol articles, which describe protocols hosted on protocols.io. Read more information on sharing protocols at https://plos.org/protocols?utm_medium=editorial-email&utm_source=authorletters&utm_campaign=protocols.

We look forward to receiving your revised manuscript.

Kind regards,

Maher Abdelraheim Titi

Academic Editor

PLOS One

Journal Requirements:

If the reviewer comments include a recommendation to cite specific previously published works, please review and evaluate these publications to determine whether they are relevant and should be cited. There is no requirement to cite these works unless the editor has indicated otherwise.

Additional Editor Comments:

The study investigated an important topic, the impact of Mediterranean Diet (LMeD), psychosocial factors, and maternal health indicators on infant birth weight categories (SGA, AGA, LGA) among Lebanese pregnant women, However, the manuscript requires substantial improvements in clarity, statistical reporting, and narrative focus before it can be considered for publication in PLOS ONE.

Major Issues

Methodological and Reporting Inconsistencies: The manuscript contains multiple inconsistencies in sampling, data collection, and variable measurement. (see my comments below).

Insufficient Statistical Reporting: Non significant regression results are omitted, Model 1 is referenced but not presented. Without full regression outputs, reviewers cannot evaluate the validity of the findings.

Several psychosocial and clinical measurement tools (PSS 10, PSQI, EPDS, BP measurement protocols) are inadequately described or incorrectly referenced (see my comments below).

The manuscript is lengthy, repetitive, lack precision and sometimes misinterprets cited literature.

A major concern relates to the reference list, as a substantial number of the cited sources are not retrievable using the information provided in the manuscript. This issue significantly undermines the credibility of the work.

Abstract

The abstract ends abruptly monitoring MAP, PP and sleep quality and promoting adherence to the LMeD). please add a concluding sentence summarizing implications.

Introduction

The manuscript is overly lengthy, with extensive detail that at times obscures the main findings. Recommendation: Streamline the Introduction and Discussion by eliminating redundancies and emphasizing the most relevant evidence to improve clarity and focus.

Study Design and Sampling

The description of the sampling strategy is insufficient and requires substantial clarification. The manuscript states that “Among 732 obstetric clinics, a total of 20 private and hospital‑based private clinics were selected using simple random sampling.” However, the process by which this random selection occurred is unclear. Please clarify the following points:

o How were the 20 clinics randomly selected? (Was a random number generator used? Or Were clinics stratified by governorate before random selection?)

o How were participants recruited within each clinic? (consecutively or randomly recruited?)

o Explain the apparent inconsistency in regional recruitment (30.7% of participants were recruited from Mount Lebanon, drawn from 3 clinics, while 12.4% were recruited from Beirut, drawn from 5 clinics, and that a minimum of 30 women were recruited from each clinic). These numbers are difficult to reconcile. If each clinic contributed approximately 30 participants, then regions with more clinics should contribute more participants. Instead, the opposite appears to be true.

o Given the unequal distribution of clinics and participants across governorates, weighting may be necessary to avoid over‑ or under‑representation of certain regions. If no weighting was applied, please justify this decision.

At Line 128, there is a clear typo error in the description of clinic locations. The manuscript refers to “South China” and “North China”, These should be corrected to: South Lebanon, North Lebanon.

Study Design

The manuscript states that “the questionnaires were readministered in the 2nd and 3rd trimesters”, yet Table 2 presents data for certain instruments (e.g., Pittsburgh Sleep Quality Index and Perceived Stress Scale) across T1, T2, and T3, implying administration in the first trimester as well. In contrast, PARmed-X for Pregnancy is only reported for T2 and T3. This inconsistency suggests a reporting bias.

Line 155 “abbreviation” SBP, DBP, MAP. PP”, line 489” LMIC”. It require the first mention of an abbreviation to be spelled out.

Line 176-177:” this tool had a good correlation (r=0.56) with the Italian MeD tool.” Saying "good correlation" for r = 0.56 is a bit subjective. In healthcare research, a correlation coefficient of r ≈ 0.5–0.6 is generally interpreted as moderate, not “good.”, you might want to phrase it more precisely: “This tool demonstrated a moderate positive correlation (r=0.56) with the Italian MeD tool”. and consider moving this statement to the first paragraph of the “Dietary assessment and adherence to the Lebanese Mediterranean diet” section to enhance readability and emphasize its importance

Line 171-172: The authors reported “in the LMeD, other main components, such as fish, red meat, poultry, and wine consumption, are missing. While this adaptation reflects cultural dietary practices, the absence of these elements may limit comparability with other Mediterranean populations and should be acknowledged as a methodological limitation.

Line 171 notes wine consumption as missing, while Line 177 explains that alcohol intake (including wine) was excluded due to cultural absence. This overlap creates redundancy and potential confusion regarding whether wine is missing due to cultural adaptation or excluded as part of alcohol intake. Consider merging these into a single, consistent explanation to improve clarity.

The LMeD score is based on tertiles of intake, but the manuscript does not specify Whether tertiles were calculated per trimester or based on baseline distribution.

Line 201-203:

o The text states that FBG values were collected only in trimesters 1 and 3, yet categorization is described as occurring during the second trimester visit. This creates confusion: how can categorization be based on a trimester where no values were collected?

o The unit “mmol/” is incomplete; it should be “mmol/L.”

Line 214: Infant birth outcomes: Clarify whether gestational age was based on LMP or ultrasound.

Line 208-212: The use of trimester-specific MAP cutoffs is appropriate; however, the rationale for applying PP cutoffs derived from a single population study with only 20 women (reference 49) requires justification.

Line 208-212: Given that the study population was drawn from multiple clinics across different geographic areas, the authors should specify whether blood pressure measurements were obtained using standardized protocols and uniform devices. This information is essential to ensure consistency, reliability, and comparability of measurements across sites. If standardized procedures or devices were not used, this should be explicitly acknowledged and discussed as a limitation of the study.

Psychosocial variables (line 220-226)

o In Lines 220–226, the authors claim that the Perceived Stress Scale (PSS-10) has been validated among the Lebanese population, but no supporting reference is provided. The appropriate citation is: Chaaya, M., Osman, H., Naassan, G., et al. (2010). Validation of the Arabic version of the Cohen Perceived Stress Scale (PSS-10) among pregnant and postpartum women. BMC Psychiatry, 10, 111. https://doi.org/10.1186/1471-244X-10-111. In this validation study, Cronbach’s alpha was used to assess internal consistency reliability, while test–retest reliability was evaluated using Spearman’s correlation coefficient. These two values should be reported separately to clearly distinguish between the two scales.

o Line 223-224: The author reported that Lebanese version is Correlated with and with a general health questionnaire (r=0.48) and EPDS (r=0.58), but was not clear from where these value came, the original article in table-2 (Chaaya 2010) reported different values (.59) and .49 respectively.”

o Lines 228–232: The study validating the Arabic version of the Pittsburgh Sleep Quality Index (PSQI) is not cited. The appropriate reference is: Suleiman K., Al-Hadid L., Duhni A. (2012). Psychometric testing of the Arabic version of the Pittsburgh Sleep Quality Index (A‑PSQI) among coronary artery disease patients in Jordan. Journal of Natural Sciences Research, 2(8), 15–20

o Line 236: The term “global sum” is ambiguous. Do you mean the “total score”? Please clarify the terminology.

o Line 237-239: The authors reported that Edinsburgh Perinatal/Postnatal Depression Scale (EPDS) tool was used to assess depression, however, the reference cit the tool Edinsburgh Postnatal Depression Scale (EPDS) 1987 which used only for POST natal. Furthermore, the authors claim that the tool has been validated among the Lebanese population, but no supporting reference is provided. The appropriate citation is “Ghubash, R., Abou-Saleh, M. T., & Daradkeh, T. K. (1997). The validity of the Arabic Edinburgh Postnatal Depression Scale. Social Psychiatry and Psychiatric Epidemiology, 32(8), 474–476. https://doi.org/10.1007/BF00789142”

Results:

The results are reported without interpretation, simply restating data already presented in the tables. The narrative should inform the reader with meaningful statements that emphasize trends and group differences. For example, instead of writing ‘Factors that differed revealed that greater adherence to the LMeD was associated in both trimesters 2 (p<0.033) and 3 (p<0.038), but not in trimester 1,’ the authors could write ‘AGA mothers consistently show better adherence to the LMeD in later trimesters.’ This style of reporting should be applied consistently across the results section to enhance clarity and impact.

There is an inconsistency in participant reporting. The authors state that 42 participants dropped out due to miscarriages or other reasons. However, the analysis (lines 275–286) describes the characteristics of 618 pregnant women, while Table 1 presents characteristics for only 576 women. Furthermore, Table 2 (Comparisons of maternal health factors across the SGA, AGA, and LGA groups) and the supplementary tables report data for 618 women, despite the earlier statement that 42 participants were excluded. This discrepancy suggests a reporting bias and requires clarification regarding the actual sample size used in each analysis.

In Table 1, Infant sex, Birth weight, Birth weight z score should be presented as percentages with appropriate units. Please revise the table to ensure clarity.

Model 1 is referenced in the text, but the corresponding results are not presented in tables or in the supplementary materials.

Table 2 is very long and difficult to interpret. Move some variables to supplementary tables is recommended.

Table 3 presents only the statistically significant adjusted variables from the multiple logistic regression model, while non-significant results are omitted—even from supplementary materials. Without access to the full regression output, including non-significant predictors, the model cannot be adequately reviewed or interpreted. The authors should provide a complete list of variables assessed, ideally in supplementary tables, to ensure methodological transparency and allow for proper peer evaluation.

Line 278: The sample appears to be highly educated, with 76% of participants holding a university degree. This raises concerns about selection bias and limits the generalizability of the findings to populations with more diverse educational and socioeconomic backgrounds. The authors should further discuss how this skewed educational profile may influence health behaviors, access to care, and study outcomes, and explicitly address these socioeconomic factors in the limitations section.

Line 314: Stress and depression were measured but were not included in the analytical models. Given that their prevalence exceeded 50% of mothers across T1, T2, and T3, the authors should discuss why these variables did not demonstrate significant associations with the outcomes. Potential explanations such as measurement timing, confounding with other psychosocial or clinical factors should be explored.

Line 395: The manuscript states that dairy products in the third trimester (T3) were associated with increased BWGA; however, Table 3 indicates that this association was observed in the second trimester.

Discussion:

The Discussion section is overly narrative and includes tangential literature.

The discussion section is relatively long, and several paragraphs repeat or restate results rather than providing interpretation. For example, the paragraph in lines 577–585 (“Pre‑pregnancy BMI, gestational weight gain (GWG) and birth outcomes”) reads more like a results summary than a critical discussion. In addition, multiple sections are overly lengthy and contain redundancies. Streamlining the discussion, removing repeated results, and focusing on interpretation, implications, and comparison is needed.

The authors used sometimes overstates causality, therefore, using cautious language such as “associated with,” “may contribute to” is highly recommended.

Lines 533–537: The Iranian study you cite does not support your findings as stated. The Iranian study reports that as the ratio of MUFA (olive oil) to SFA increases, gestational weight gain decreases—meaning higher olive intake is associated with lower GWG. In contrast, your results indicate that women who delivered LGA infants had higher olive intake in Trimester 2. These two interpretations appear contradictory. Please clarify this discrepancy and explain how the cited study supports your findings. You may need to discuss potential confounding such as total energy intake, cooking methods.

Line 495: The author reported that LMeD adherence did not enter models for SGA or LGA, yet specific LMeD components did. This distinction needs clearer framing in the discussion: why does the overall pattern fail to predict risk while individual components like olive oil in T2 increase LGA risk?

Line 498-499: Is there any explanation why the AGA mothers consistently show better adherence to the LMeD in later trimesters.

Line 555: Eggs show the highest positive coefficient among the listed food groups. It is recommended to explicitly link this finding to its nutritional significance. Eggs are a high‑quality protein source and provide key nutrients such as choline and essential fatty acids, which play critical roles in fetal brain development, cellular growth, and overall pregnancy outcomes. Highlighting these mechanisms would strengthen the interpretation of this result.

Line 696: The authors describe the association between poor sleep in T3 and increased LGA risk as a “novel psychosocial dimension.” I disagree with this characterization, as multiple previous studies have already reported similar associations between maternal sleep disturbances and fetal overgrowth. The authors should revise this statement to accurately reflect the existing body of evidence.

Others

The authors state that the data are available within the manuscript and supporting files. However, PLOS ONE requires that the minimal data set be provided in a form that allows full replication of the analyses. In the current submission, non‑significant results appear to be omitted—even from the supplementary materials. Without access to the complete regression outputs, including non‑significant predictors, it is not possible to adequately review or interpret the models

A major concern relates to the reference list, as a substantial number of the cited sources are not retrievable using the information provided in the manuscript. This issue significantly undermines the credibility of the work. Ensure all references are current and correctly formatted and are complete (some missing journal names or years). Reference 57 appears to be a duplicate of Reference 54.

[Note: HTML markup is below. Please do not edit.]

Reviewers' comments:

Reviewer's Responses to Questions

**Comments to the Author**

1. Is the manuscript technically sound, and do the data support the conclusions?

The manuscript must describe a technically sound piece of scientific research with data that supports the conclusions. Experiments must have been conducted rigorously, with appropriate controls, replication, and sample sizes. The conclusions must be drawn appropriately based on the data presented.

Reviewer #1: Yes

Reviewer #2: Yes

**********

2. Has the statistical analysis been performed appropriately and rigorously?

Reviewer #1: Yes

Reviewer #2: Yes

**********

3. Have the authors made all data underlying the findings in their manuscript fully available?

The PLOS Data policy requires authors to make all data underlying the findings described in their manuscript fully available without restriction, with rare exception (please refer to the Data Availability Statement in the manuscript PDF file). The data should be provided as part of the manuscript or its supporting information, or deposited to a public repository. For example, in addition to summary statistics, the data points behind means, medians and variance measures should be available. If there are restrictions on publicly sharing data—e.g. participant privacy or use of data from a third party—those must be specified.

Reviewer #1: Yes

Reviewer #2: Yes

**********

4. Is the manuscript presented in an intelligible fashion and written in standard English?

PLOS ONE does not copyedit accepted manuscripts, so the language in submitted articles must be clear, correct, and unambiguous. Any typographical or grammatical errors should be corrected at revision, so please note any specific errors here.

Reviewer #1: Yes

Reviewer #2: Yes

**********

5. Review Comments to the Author

Please use the space provided to explain your answers to the questions above. You may also include additional comments for the author, including concerns about dual publication, research ethics, or publication ethics. (Please upload your review as an attachment if it exceeds 20,000 characters)

Reviewer #1: Minor Revision

Abstract :

Some editing mistakes (L41 « . ») / review the conclusion (nonverbal phrase).

Introduction :

L108 : abbreviation of Body mass Index (BMI)

Materials and methods :

L114 : how did u define the study being « national » ?, while the recrutment was exclusively conducted in private clinics and not public hospitals and the recrutment from the capital Beirut was just 12.4%.

L119 : If women were recruted in their 1st trimester of gestation, are the structural and chromosomal anomalies diagnosted at this early stage of pregnancy ?

L126 : how did you ended up with the 732 obstetric clinics ? was the data from the ministry of health ? or other organisation ?

L128 : The study is conducted in Lebanon and not in South or North China like mentionned.

L133 : Provide the ethical consent of the affiliated academic institution.

L149 : Provide more information about the physical activity status of participating pregnant women. Why the research team did not rely on a validated tool such as the PPAQ ?

L153 : why anemia was assessed ? and not explored later in regard to neonatal outcomes ? were the participants supplemented ?

L160/161 : Pls note that this FFQ was not validated among pregnant Lebanese women. This should be cited in the limitations.

L168 : dried fruits or nuts ? since oleaginous fruits are more consumed by the Lebanese population.

L171 : fish, red meat and wine are major food components implicated in measuring the adherence in the mediteranean diet. Provide an explanation why they were excluded from the initial tool, since it may affect the results and not considered a reliable tool?

L173 : Provide a detailed measurement analysis of this tool. How was the final adherence to Med Diet among this sample ? Compare and cite with studies assessing dietary patterns among Lebanese pregnant women.

L177 : Reference 35 is Buysse DJ, et al. Pittsburgh Sleep Quality Index (PSQI). Sleep. 1989;12(1):65-72 and not an italian validated Med tool !

L188 : change the word « according » written twice in the same phrase.

L204 : is it 50 g or 75 g or 1g / kg body weight ? (75g is more widely used)

L214 : cite the other birth outcomes, since they were mentionned in the result part.

L250 : how was the physical activity assessed and categorized ?

L280 : pls correct all values ( 63.4% instead of 67.6% , 32.7% instead of 32.4% and change « or » with « and » )

L293 : Add N= … the number of participants in the title / L336 too

Table 1 Some editing mistakes « , » near GWG T3 and remove « . » near 58 (Boy) /

L315 : Delivery mode was not cited in the results. Why this variable was not taken into account during the analyses?

L316 Participants suffering from delivery complications, were they dropped from the study ? Data is not reported in the tables.

L340 : the format of the footer in table 1 is different from table 2, 3… pls review the formatting

Discussion :

- No mention about the smoking status of the participants, especially that it may be linked to negative neonatal outcomes and linked to HBP.

- L503 editing mistake « . » after ref 58.

- L536 : higher olive intake ? or olive oil ? and specifically how much in ml ?

- L559 : fruits or nuts ?

- L571 – 573 : the statement proposed is not clear. Pls rephrase.

- L577 : replace reference 79, by recent published studies conducted among Lebanese pregnant women in regard to neonatal outcomes.

- L707 editing mistake « . » after « findings ».

Limitations :

L713 Mention about the limitations of the tool used to assess the Med-diet adherence and the lack of assessment of not only energy intake but also macro and micronutrient and fiber content of their diet, since neonatal outcomes are highly influenced by caloric intake and the contribution of fat and protein during pregnancy.

References :

Review pls the referencing of some citations too (57, 74..). Provide the names of all authors, unless if the publication is authored by more than 6 authors and not just the first author with al. The name of some contributing authors are lacking in the citations.

Reviewer #2: The authors are commended for conducting such a robust study in a population where the is a

paucity of data related to pregnancy outcomes.

However, there are several concerns about some of the unusual finding from this study such as

associations of healthy fat intake (olive oil) and wheat with poor pregnancy outcomes and (LGA and

SGA respectively), high MAP during pregnancy being associated with LGA and poor sleep being

associated with LGA. The authors are encouraged to robustly explain these findings and how they

contradict current understandings in this field.

Detailed comments Uploaded as an attachment

**********

6. PLOS authors have the option to publish the peer review history of their article (what does this mean?). If published, this will include your full peer review and any attached files.

If you choose “no”, your identity will remain anonymous but your review may still be made public.

**Do you want your identity to be public for this peer review?** For information about this choice, including consent withdrawal, please see our Privacy Policy.

Reviewer #1: **Yes:** Tatiana Papazian

Reviewer #2: **Yes:** Shrish Budree, MD, PhD

**********

[NOTE: If reviewer comments were submitted as an attachment file, they will be attached to this email and accessible via the submission site. Please log into your account, locate the manuscript record, and check for the action link "View Attachments". If this link does not appear, there are no attachment files.]

To ensure your figures meet our technical requirements, please review our figure guidelines: https://journals.plos.org/plosone/s/figures

You may also use PLOS’s free figure tool, NAAS, to help you prepare publication quality figures: https://journals.plos.org/plosone/s/figures#loc-tools-for-figure-preparation.

NAAS will assess whether your figures meet our technical requirements by comparing each figure against our figure specifications.

Attachment Submitted filename: Summary of Comments on PONE-D-25-31196_SB_12.7.pdf

# Author comment: Author response to Decision Letter 1

**DOI:** 10.1371/journal.pone.0351497.r002

11 Feb 2026

We sincerely thank the Editor and Reviewers for their detailed and constructive feedback. We have carefully addressed each comment and revised the manuscript accordingly. Changes have been highlighted in the revised manuscript. Our responses are presented below in a point-by-point format.

Editor and Major Comments

Abstract

Comment: The abstract ends abruptly monitoring MAP, PP and sleep quality and promoting adherence to the LMeD. Please add a concluding sentence summarizing implications.

Response: We added a concluding sentence highlighting the study implications:

“Screening for family history of diabetes and macrosomia, targeting trimester-specific gestational weight gain, monitoring maternal blood pressure, pulse pressure, and sleep quality, and promoting adherence to the Lebanese Mediterranean diet are important strategies to optimize infant birth outcomes.”

Introduction

Comment: The manuscript is overly lengthy, with extensive detail that at times obscures the main findings. Recommendation: Streamline the Introduction and Discussion by eliminating redundancies and emphasizing the most relevant evidence.

Response: Introduction and Discussion were revised to remove redundancies, improve clarity, and emphasize literature most relevant to maternal diet, psychosocial factors, and infant birth outcome.

Study Design and Sampling

Comment: The description of the sampling strategy is insufficient and requires substantial clarification. The manuscript states that “Among 732 obstetric clinics, a total of 20 private and hospital based private clinics were selected using simple random sampling.” However, the process by which this random selection occurred is unclear. Please clarify the following points:

o How were the 20 clinics randomly selected? (Was a random number generator used? Or Were clinics stratified by governorate before random selection?)

o How were participants recruited within each clinic? (consecutively or randomly recruited?)

o Explain the apparent inconsistency in regional recruitment (30.7% of participants were recruited from Mount Lebanon, drawn from 3 clinics, while 12.4% were recruited from Beirut, drawn from 5 clinics, and that a minimum of 30 women were recruited from each clinic). These numbers are difficult to reconcile. If each clinic contributed approximately 30 participants, then regions with more clinics should contribute more participants. Instead, the opposite appears to be true.

o Given the unequal distribution of clinics and participants across governorates, weighting may be necessary to avoid over or under representation of certain regions. If no weighting was applied, please justify this decision

Response:

• Clinic Selection: The 20 clinics were stratified by governorate. This was added to the study participants section line 124-126.

• Participant Recruitment: Participants were randomly recruited within each clinic at their first prenatal visit. This was added to the study participants section line 128.

• Regional Distribution: Clinics in Mount Lebanon recruited more women per clinic to achieve target sample, explaining apparent discrepancies

• Weighting: No post-stratification weighting was applied; governorate of residence was included as an adjustment variable in analyses to account for regional differences.

Comment: Line 128 refers to “South China” and “North China.”

Response: Corrected to South Lebanon and North Lebanon in line 126-127.

Study Design

Comment: The manuscript states that “the questionnaires were re-administered in the 2nd and 3rd trimesters”, yet Table 2 presents data for certain instruments (e.g., Pittsburgh Sleep Quality Index and Perceived Stress Scale) across T1, T2, and T3, implying administration in the first trimester as well. In contrast, PARmed-X for Pregnancy is only reported for T2 and T3. This inconsistency suggests a reporting bias

Response: Clarified in the Methods lines 149-150 that the PSS-10, PSQI and PARmed-X were administered at all three trimesters. Table 2 was updated to reflect this.

Comment: Abbreviations need to be spelled out at first mention (SBP, DBP, MAP, PP, LMIC).

Response: All abbreviations have been defined at first mention in the revised manuscript.

Comment: Line 176-177:” this tool had a good correlation (r=0.56) with the Italian MeD tool.” Saying "good correlation" for r = 0.56 is a bit subjective. In healthcare research, a correlation coefficient of r ≈ 0.5–0.6 is generally interpreted as moderate, not “good.”, you might want to phrase it more precisely: “This tool demonstrated a moderate positive correlation (r=0.56) with the Italian MeD tool”. and consider moving this statement to the first paragraph of the “Dietary assessment and adherence to the Lebanese Mediterranean diet” section to enhance readability and emphasize its importance

Response: Changed to:

“This tool demonstrated a moderate positive correlation (r=0.56) with the Italian MeD tool” and moved to the first paragraph of the Dietary Assessment section Lines 164-165.

Comment: Line 171-172: The authors reported “in the LMeD, other main components, such as fish, red meat, poultry, and wine consumption, are missing. While this adaptation reflects cultural dietary practices, the absence of these elements may limit comparability with other Mediterranean populations and should be acknowledged as a methodological limitation

Response: This has been added in the limitations section lines 538-540.

Comment: Line 171 notes wine consumption as missing, while Line 177 explains that alcohol intake (including wine) was excluded due to cultural absence. This overlap creates redundancy and potential confusion regarding whether wine is missing due to cultural adaptation or excluded as part of alcohol intake. Consider merging these into a single, consistent explanation to improve clarity.

Response: The sentence about alcohol intake has been deleted to prevent confusion line 178. It has been excluded in the original validation by Naja et al. 2015 as part of cultural absence. We mentioned that these elements did not load high on the pattern the LMeD lines 174-175.

Comment: The LMeD score is based on tertiles of intake, but the manuscript does not specify whether tertiles were calculated per trimester or based on baseline distribution.

Response: LMeD tertiles were calculated per trimester. This has been clarified in Methods lines 178.

Comment: The text states that FBG values were collected only in trimesters 1 and 3, yet categorization is described as occurring during the second trimester visit. This creates confusion: how can categorization be based on a trimester where no values were collected?

o The unit “mmol/” is incomplete; it should be “mmol/L. line 201

Response: There has been a misplacement of the punctuation mark. We have corrected this section to indicate that FBG were collected in trimesters 1 and 3, while GDM diagnosis was made in trimester 2 based on the two-step OGTT test, and the unit has been corrected to mmol/L line 204.

Comment: Line 214: Infant birth outcomes: Clarify whether gestational age was based on LMP or ultrasound. Line 208-212: The use of trimester-specific MAP cutoffs is appropriate; however, the rationale for applying PP cutoffs derived from a single population study with only 20 women (reference 49) requires justification. Line 208-212: Given that the study population was drawn from multiple clinics across different geographic areas, the authors should specify whether blood pressure measurements were obtained using standardized protocols and uniform devices. This information is essential to ensure consistency, reliability, and comparability of measurements across sites. If standardized procedures or devices were not used, this should be explicitly acknowledged and discussed as a limitation of the study.

Response: Gestational age was based on last menstrual period (LMP) confirmed by first-trimester ultrasound. This sentence has been added to the methods section Line 224-225.

As for pulse pressure, previous studies have defined these categories based on the distribution of measurements or means within their respective cohorts. In a recent study by Sampson et al., 2024, they chose to use the dichotomous cutoff of PP > 55 mmHg based on previous studies (Mullan et al., 2021; Thadhani et al., 2001; Maykin et al., 2024). The study that we cited before was the most recent one at the time. Reference 49 has been updated to reflect the new study by Sampson et al. 2024 lines 214-216. Blood pressure measurements were obtained using standardized protocols. A sentence in the limitations was added that the devices used by gynecologists were not uniform line 543-544.

References below:

Reference: Mullan S.J., Vricella L.K., Edwards A.M., Powel J.E., Ong S.K., Li X., Tomlinson T.M. Pulse pressure as a predictor of response to treatment for severe hypertension in pregnancy. Am. J. Obstet. Gynecol. MFM. 2021;3:100455. doi: 10.1016/j.ajogmf.2021.100455. [DOI] [PubMed] [Google Scholar][Ref list]

Thadhani R., Ecker J.L., Kettyle E., Sandler L., Frigoletto F.D., Jr. Pulse pressure and risk of preeclampsia: A prospective study. Obstet. Gynecol. 2001;97:515–520. doi: 10.1016/s0029-7844(00)01192-3. [DOI] [PubMed] [Google Scholar][Ref list]

Maykin M.M., Mercer E., Saiki K.M., Kaneshiro B., Miller C.B., Tsai P.-J.S. Furosemide to lower antenatal severe hypertension: A randomized placebo-controlled trial. Am. J. Obstet. Gynecol. MFM. 2024;6:101348. doi: 10.1016/j.ajogmf.2024.101348.

Sampson R, Davis S, Wong R, Baranco N, Silverman RK. Pulse Pressure as a Hemodynamic Parameter in Preeclampsia with Severe Features Accompanied by Fetal Growth Restriction. J Clin Med. 2024 Jul 24;13(15):4318. doi: 10.3390/jcm13154318. PMID: 39124585; PMCID: PMC11312723.

Comment: Psychosocial variables (line 220-226)

o In Lines 220–226, the authors claim that the Perceived Stress Scale (PSS-10) has been validated among the Lebanese population, but no supporting reference is provided. The appropriate citation is: Chaaya, M., Osman, H., Naassan, G., et al. (2010). Validation of the Arabic version of the Cohen Perceived Stress Scale (PSS-10) among pregnant and postpartum women. BMC Psychiatry, 10, 111. https://doi.org/10.1186/1471-244X-10-111. In this validation study, Cronbach’s alpha was used to assess internal consistency reliability, while test–retest reliability was evaluated using Spearman’s correlation coefficient. These two values should be reported separately to clearly distinguish between the two scales.

o Line 223-224: The author reported that Lebanese version is Correlated with and with a general health questionnaire (r=0.48) and EPDS (r=0.58), but was not clear from where these value came, the original article in table-2 (Chaaya 2010) reported different values (.59) and .49 respectively.”

Response: We thank the reviewer for pointing out the missing reference and clarifying the reliability and validity values. We have updated the manuscript to correctly cite Chaaya et al. (2010) and report Cronbach’s alpha (0.74) and test–retest reliability (Spearman’s r = 0.49) separately. Correlations with the General Health Questionnaire (r = 0.59) and EPDS (r = 0.49) have also been corrected to reflect the original validation study lines 231-235.

Comment: Lines 228–232: The study validating the Arabic version of the Pittsburgh Sleep Quality Index (PSQI) is not cited. The appropriate reference is: Suleiman K., Al-Hadid L., Duhni A. (2012). Psychometric testing of the Arabic version of the Pittsburgh Sleep Quality Index (A‑PSQI) among coronary artery disease patients in Jordan. Journal of Natural Sciences Research, 2(8), 15–20

Response: We have updated the reference for the validation study line 243.

o Line 236: The term “global sum” is ambiguous. Do you mean the “total score”? Please clarify the terminology.

Response: This has been corrected line 240.

o Line 237-239: The authors reported that Edinsburgh Perinatal/Postnatal Depression Scale (EPDS) tool was used to assess depression, however, the reference cite the tool Edinsburgh Postnatal Depression Scale (EPDS) 1987 which used only for POST natal. Furthermore, the authors claim that the tool has been validated among the Lebanese population, but no supporting reference is provided. The appropriate citation is “Ghubash, R., Abou-Saleh, M. T., & Daradkeh, T. K. (1997). The validity of the Arabic Edinburgh Postnatal Depression Scale. Social Psychiatry and Psychiatric Epidemiology, 32(8), 474–476. https://doi.org/10.1007/BF00789142”

Response: The name of the tool was corrected to Edinsburgh Postnatal Depression Scale (EPDS) tool line 246. The citation for validation has been updated line 249. The word Lebanese has been replaced with Arabic population line 248.

Results

Comment: Results simply restate tables; sample size inconsistencies (618 vs 576).

Response:

• Narratives were rewritten to interpret findings, emphasizing trends and group differences.

• Sample size inconsistencies were clarified: 618 women is the exact number of pregnant women who completed the study. The 42 participants dropped from the initial sample size of N=660. This has been added to the methods section to clarify and removed from the table line 158-159.

Comment: Model 1 referenced but not presented; non-significant regression results omitted.

Response: Full regression tables, including Model 1 and non-significant variable values, have been added to Supplementary Tables S1–S3 for transparency line 1019-1025.

Comment: Tables restructured for clarity (percentages for categorical variables, moved long variables to Supplementary Tables).

Response: Tables revised per reviewer recommendations.

Comment: Education skew discussed as a limitation.

Response: We added in the limitations the following statement “Limitations include reliance on self-reported data, a highly educated sample (76%) which may skew results and limit generalizability” line 536.

Comment: Stress and depression not included in models; discuss prevalence >50%.

Response: Explanation that these variables were not significant in multivariable models has been explained in the results line 325, discussion line 517, and limitations lines 537.

Comment: Dairy association with BWGA corrected (T2, not T3).

Response: Table 3 and Results text updated accordingly line 359.

Discussion

Comment: The Discussion section is overly narrative and includes tangential literature.

The discussion section is relatively long, and several paragraphs repeat or restate results rather than providing interpretation. For example, the paragraph in lines 577–585 (“Pre pregnancy BMI, gestational weight gain (GWG) and birth outcomes”) reads more like a results summary than a critical discussion. In addition, multiple sections are overly lengthy and contain redundancies. Streamlining the discussion, removing repeated results, and focusing on interpretation, implications, and comparison is needed. The authors used sometimes overstates causality, therefore, using cautious language such as “associated with,” “may contribute to” is highly recommended.

Response: We have streamlined discussion to emphasize interpretation, implications, and comparison with literature. Language revised to “associated with” rather than implying causality throughout the discussion.

Comment: Lines 533–537: The Iranian study you cite does not support your findings as stated. The Iranian study reports that as the ratio of MUFA (olive oil) to SFA increases, gestational weight gain decreases—meaning higher olive intake is associated with lower GWG. In contrast, your results indicate that women who delivered LGA infants had higher olive intake in Trimester 2. These two interpretations appear contradictory. Please clarify this discrepancy and explain how the cited study supports your findings. You may need to discuss potential confounding such as total energy intake, cooking methods.

Response: A statement about the potential confounding (energy intake, cooking methods) has been added to the limitations lines 540-541

The reference has been amended in the text line 482 and in the reference list “66”.

Comment: Line 495: The author reported that LMeD adherence did not enter models for SGA or LGA, yet specific LMeD components did. This distinction needs clearer framing in the

Attachment Submitted filename: Response to Reviewers.docx

# Peer review: Decision Letter 1

**DOI:** 10.1371/journal.pone.0351497.r003

**Authors:** Maher Abdelraheim Titi

15 Apr 2026

PONE-D-25-31196R1

Predictors of Infant Birth Weights: Role of the Lebanese Mediterranean Diet, Psychosocial Factors and Maternal Health Status

PLOS One

Dear Dr. Fares,

Thank you for submitting your manuscript to PLOS ONE. After careful consideration, we feel that it has merit but does not fully meet PLOS ONE’s publication criteria as it currently stands. Therefore, we invite you to submit a revised version of the manuscript that addresses the points raised during the review process.

The authors have satisfactorily addressed several of the previous comments; however, a number of important issues remain unresolved. Kindly see my comments below.

Please submit your revised manuscript by May 30 2026 11:59PM. If you will need more time than this to complete your revisions, please reply to this message or contact the journal office at plosone@plos.org. When you're ready to submit your revision, log on to https://www.editorialmanager.com/pone/ and select the 'Submissions Needing Revision' folder to locate your manuscript file.

Please include the following items when submitting your revised manuscript:

- A letter that responds to each point raised by the academic editor and reviewer(s). You should upload this letter as a separate file labeled 'Response to Reviewers'.
- A marked-up copy of your manuscript that highlights changes made to the original version. You should upload this as a separate file labeled 'Revised Manuscript with Track Changes'.
- An unmarked version of your revised paper without tracked changes. You should upload this as a separate file labeled 'Manuscript'.

If you would like to make changes to your financial disclosure, please include your updated statement in your cover letter. Guidelines for resubmitting your figure files are available below the reviewer comments at the end of this letter.

If applicable, we recommend that you deposit your laboratory protocols in protocols.io to enhance the reproducibility of your results. Protocols.io assigns your protocol its own identifier (DOI) so that it can be cited independently in the future. For instructions see: https://journals.plos.org/plosone/s/submission-guidelines#loc-laboratory-protocols. Additionally, PLOS ONE offers an option for publishing peer-reviewed Lab Protocol articles, which describe protocols hosted on protocols.io. Read more information on sharing protocols at https://plos.org/protocols?utm_medium=editorial-email&utm_source=authorletters&utm_campaign=protocols.

As the corresponding author, your ORCID iD is verified in the submission system and will appear in the published article. PLOS supports the use of ORCID, and we encourage all coauthors to register for an ORCID iD and use it as well. Please encourage your coauthors to verify their ORCID iD within the submission system before final acceptance, as unverified ORCID iDs will not appear in the published article. *Only* the individual author can complete the verification step; PLOS staff *cannot* verify ORCID iDs on behalf of authors.

We look forward to receiving your revised manuscript.

Kind regards,

Maher Abdelraheim Titi

Academic Editor

PLOS One

**Journal Requirements:**

1. If the reviewer comments include a recommendation to cite specific previously published works, please review and evaluate these publications to determine whether they are relevant and should be cited. There is no requirement to cite these works unless the editor has indicated otherwise.

2. Please review your reference list to ensure that it is complete and correct. If you have cited papers that have been retracted, please include the rationale for doing so in the manuscript text, or remove these references and replace them with relevant current references. Any changes to the reference list should be mentioned in the rebuttal letter that accompanies your revised manuscript. If you need to cite a retracted article, indicate the article’s retracted status in the References list and also include a citation and full reference for the retraction notice.

**Additional Editor Comments:**

The authors have satisfactorily addressed several of the previous comments; however, a number of important issues remain unresolved:

• Manuscript length and focus: I still believe that the manuscript is overly lengthy, with extensive detail that at times obscures the main findings. Further streamlining of the Introduction and Discussion is needed, particularly by removing redundancies and focusing on the key messages.

• Line 125 – Clarification of clinic types: “A total of 20 private and hospital-based private clinics were stratified by governorate.” Please clarify the distinction between private clinics and hospital-based private clinics. How do these settings differ operationally or structurally, and why is this distinction relevant to the study?

• Line 128 – Definition of “first prenatal visit” and recruitment strategy

“Participants were then randomly recruited within each clinic at their first prenatal visit.”

Please clarify what is meant by “their first prenatal visit.” Does this refer to women attending their first-ever prenatal visit at that clinic during the study period? If so, this appears closer to consecutive recruitment rather than random recruitment.

• Lines 112–113 – Governorate distribution and percentages: “Governorates of Lebanon that included Mount Lebanon (30.7%), Beirut (12.4%), Bekaa (16.4%), South Lebanon (and Nabatieh) (29.4%), and Akkar (3.7%).”

The percentages sum to 92.6%, not 100%.

o Please provide the total number of participants per governorate in addition to percentages.

o Ensure that these numbers and percentages are consistent with what is reported in lines 126–127 and elsewhere in the manuscript.

• Pulse pressure cutoff and supporting literature

I recommend citing additional studies with larger populations and clearly defined pulse pressure (PP) thresholds to justify the cutoff used in this study. the rationale for the PP cutoff adopted in this study is not sufficiently supported by the literature and should be more clearly justified, For example:

o Mullan et al., 2021: PP cutoff 55 mmHg

o Thadhani et al., 2001: PP cutoff 50 mmHg

o Maykin et al., 2024: furosemide RCT that did not define or analyze a PP threshold.

o Sampson et al., 2024: PP cutoff 50 mmHg

In its current form,

• Lines 158–159: “Initially, 660 participants were recruited; however, 42 dropped out due to miscarriage or unwillingness to participate, making the total sample size N = 618.”

This statement is more appropriate for the Results section rather than the Methods. Please relocate this description of participant flow to the Results

• Table 1 –It would be preferable to display the units in parentheses (e.g., kg, %) and to standardize unit presentation across all variables in Table 1.

• Supplementary tables : Please ensure that the numbering and labeling of all supplementary tables are consistent between the text and the supplementary material.

• Table 3 –dairy products are reported as significantly associated with increased BWGA. Please verify whether this association pertains to T1 or T2 and ensure that the text and table are fully consistent and clearly labeled.

• Reference list: A major concern remains regarding the reference list. The references need to be revised carefully for accuracy, and consistency. Specific issues include, but are not limited to:

o The reference for Martínez-Galiano et al. (“Effect of Adherence to a Mediterranean Diet and Olive Oil Intake during Pregnancy on Risk of Small for Gestational Age Infants”) appears incomplete and/or incorrectly formatted. Please provide full citation details and numbering.

o Line 235: The cited article number “34” appears to be incorrect; please verify and correct.

o Line 470: The reference cited here should be checked for accuracy and consistency with the reference list.

o Lines 485–486: The reference cited to support the authors’ claim in these lines appears inappropriate or not directly supportive of the statement. Please revise the citation or modify the claim to align with the evidence.

[Note: HTML markup is below. Please do not edit.]

Reviewers' comments:

Reviewer's Responses to Questions

**Comments to the Author**

1. If the authors have adequately addressed your comments raised in a previous round of review and you feel that this manuscript is now acceptable for publication, you may indicate that here to bypass the “Comments to the Author” section, enter your conflict of interest statement in the “Confidential to Editor” section, and submit your "Accept" recommendation.

Reviewer #1: All comments have been addressed

**********

2. Is the manuscript technically sound, and do the data support the conclusions?

The manuscript must describe a technically sound piece of scientific research with data that supports the conclusions. Experiments must have been conducted rigorously, with appropriate controls, replication, and sample sizes. The conclusions must be drawn appropriately based on the data presented.

Reviewer #1: Yes

**********

3. Has the statistical analysis been performed appropriately and rigorously?

Reviewer #1: Yes

**********

4. Have the authors made all data underlying the findings in their manuscript fully available?

The PLOS Data policy requires authors to make all data underlying the findings described in their manuscript fully available without restriction, with rare exception (please refer to the Data Availability Statement in the manuscript PDF file). The data should be provided as part of the manuscript or its supporting information, or deposited to a public repository. For example, in addition to summary statistics, the data points behind means, medians and variance measures should be available. If there are restrictions on publicly sharing data—e.g. participant privacy or use of data from a third party—those must be specified.

Reviewer #1: Yes

**********

5. Is the manuscript presented in an intelligible fashion and written in standard English?

PLOS ONE does not copyedit accepted manuscripts, so the language in submitted articles must be clear, correct, and unambiguous. Any typographical or grammatical errors should be corrected at revision, so please note any specific errors here.

Reviewer #1: Yes

**********

6. Review Comments to the Author

Please use the space provided to explain your answers to the questions above. You may also include additional comments for the author, including concerns about dual publication, research ethics, or publication ethics. (Please upload your review as an attachment if it exceeds 20,000 characters)

Reviewer #1: All points raised during the peer-review process have been modified and the manuscript in its final version is acceptable for publication.

**********

7. PLOS authors have the option to publish the peer review history of their article (what does this mean?). If published, this will include your full peer review and any attached files.

If you choose “no”, your identity will remain anonymous but your review may still be made public.

**Do you want your identity to be public for this peer review?** For information about this choice, including consent withdrawal, please see our Privacy Policy.

Reviewer #1: **Yes:** Tatiana Wahanian Papazian

**********

[NOTE: If reviewer comments were submitted as an attachment file, they will be attached to this email and accessible via the submission site. Please log into your account, locate the manuscript record, and check for the action link "View Attachments". If this link does not appear, there are no attachment files.]

To ensure your figures meet our technical requirements, please review our figure guidelines: https://journals.plos.org/plosone/s/figures

You may also use PLOS’s free figure tool, NAAS, to help you prepare publication quality figures: https://journals.plos.org/plosone/s/figures#loc-tools-for-figure-preparation.

NAAS will assess whether your figures meet our technical requirements by comparing each figure against our figure specifications.

# Author comment: Author response to Decision Letter 2

**DOI:** 10.1371/journal.pone.0351497.r004

4 May 2026

Point by Point Response

• Manuscript length and focus: I still believe that the manuscript is overly lengthy, with extensive detail that at times obscures the main findings. Further streamlining of the Introduction and Discussion is needed, particularly by removing redundancies and focusing on the key messages.

Response: Thank you for your response. Both introduction and discussion have been streamlined to focus on the key messages and remove redundancies.

• Line 125 – Clarification of clinic types: “A total of 20 private and hospital-based private clinics were stratified by governorate.” Please clarify the distinction between private clinics and hospital-based private clinics. How do these settings differ operationally or structurally, and why is this distinction relevant to the study?

Response: To our knowledge, there are no publicly available national data in Lebanon that quantify the number of obstetric clinics by type (private vs hospital-based private). This is partly due to the absence of a centralized registry that classifies outpatient facilities according to their structural affiliation.

However, available evidence suggests that obstetric care in Lebanon is predominantly delivered in the private sector. For example, a national study reported that approximately 64.9% of obstetricians primarily practice in private clinics, while 21.6% practice in hospital-based private clinics (El Khoury et al., 2024).

Given this distribution and the overlap in practice settings among physicians, our study stratified clinics by type to ensure representation of both independent and hospital-affiliated outpatient care environments rather than to reflect exact national counts.

We have the added the following sentence in lines 111-112: The sample was proportionally allocated to include 15 private clinics and 5 hospital-based private clinics

Reference: El Khoury T, Rebeiz MC, Abi Zeid B, Mansour S, Yared G, El Kak F, Akik C, McCall SJ. An assessment of the content of antenatal care provided by obstetricians in Lebanon: A cross-sectional study. PLOS Glob Public Health. 2024 Nov 4;4(11):e0003853. doi: 10.1371/journal.pgph.0003853. PMID: 39495764; PMCID: PMC11534237.

• Line 128 – Definition of “first prenatal visit” and recruitment strategy

“Participants were then randomly recruited within each clinic at their first prenatal visit.”

Please clarify what is meant by “their first prenatal visit.” Does this refer to women attending their first-ever prenatal visit at that clinic during the study period? If so, this appears closer to consecutive recruitment rather than random recruitment.

Response: Thank you for your comment. I have adjusted lines 115-118 to the following: Participants were consecutively recruited within each clinic at their first prenatal visit during the study period. This refers to women presenting for their initial antenatal consultation for the current pregnancy, all of whom were in their first trimester (<12 weeks of gestation) at the time of enrollment.

• Lines 112–113 – Governorate distribution and percentages: “Governorates of Lebanon that included Mount Lebanon (30.7%), Beirut (12.4%), Bekaa (16.4%), South Lebanon (and Nabatieh) (29.4%), and Akkar (3.7%).”

The percentages sum to 92.6%, not 100%.

Response: Thank you for your response. There was a typing mistake and we have edited the sentence to include the 6th governorate which is North Lebanon (7.4%) line 99.

o Please provide the total number of participants per governorate in addition to percentages.

o Ensure that these numbers and percentages are consistent with what is reported in lines 126–127 and elsewhere in the manuscript.

Response: We have added next to the percentage the total number of participants for each governorate Lines 112-115.

• Pulse pressure cutoff and supporting literature

I recommend citing additional studies with larger populations and clearly defined pulse pressure (PP) thresholds to justify the cutoff used in this study. the rationale for the PP cutoff adopted in this study is not sufficiently supported by the literature and should be more clearly justified, For example:

o Mullan et al., 2021: PP cutoff 55 mmHg

o Thadhani et al., 2001: PP cutoff 50 mmHg

o Maykin et al., 2024: furosemide RCT that did not define or analyze a PP threshold.

o Sampson et al., 2024: PP cutoff 50 mmHg

Response: We thank the reviewer for this important comment. We would like to clarify that, in our study, pulse pressure (PP) was not categorized using a predefined cutoff. Instead, PP was analyzed as a continuous variable in all statistical models.

While several studies have proposed thresholds (e.g., 50–55 mmHg), these cutoffs are not consistently defined across the literature and vary depending on study population and clinical context. For instance, prior research has highlighted that there is no universally accepted definition of “elevated” pulse pressure in pregnancy, and thresholds are often derived empirically within individual cohorts rather than standardized across studies.

Given this variability and lack of consensus, we opted to retain PP as a continuous variable to preserve statistical power and avoid arbitrary categorization. This approach is methodologically robust and allows for a more precise assessment of the association between PP and the outcomes of interest.

I have updated the methods section to reflect the above justification lines 201-208.

In its current form,

• Lines 158–159: “Initially, 660 participants were recruited; however, 42 dropped out due to miscarriage or unwillingness to participate, making the total sample size N = 618.”

This statement is more appropriate for the Results section rather than the Methods. Please relocate this description of participant flow to the Results

Response: Thank you for your comment. We have relocated this sentence to the results section line 276-277.

• Table 1 –It would be preferable to display the units in parentheses (e.g., kg, %) and to standardize unit presentation across all variables in Table 1.

Response: The table has been updated to reflect units in parentheses

• Supplementary tables : Please ensure that the numbering and labeling of all supplementary tables are consistent between the text and the supplementary material.

Response: All supplementary tables have been cross-checked with the text, We have fixed the naming of the tables to ensure consistency.

• Table 3 –dairy products are reported as significantly associated with increased BWGA. Please verify whether this association pertains to T1 or T2 and ensure that the text and table are fully consistent and clearly labeled.

Response: The text has been updated to reflect associations of dairy products within the tables in both T1 and T2 for BWGA lines 353-354., while the association of dairy products with AGA remains only at T1 lines 361-362.

• Reference list: A major concern remains regarding the reference list. The references need to be revised carefully for accuracy, and consistency. Specific issues include, but are not limited to:

o The reference for Martínez-Galiano et al. (“Effect of Adherence to a Mediterranean Diet and Olive Oil Intake during Pregnancy on Risk of Small for Gestational Age Infants”) appears incomplete and/or incorrectly formatted. Please provide full citation details and numbering.

Response: I rechecked the reference of Martinez-Galiano and it has been correctly cited. I have also edited the reference list to make sure there are consistencies among the text and reference list.

o Line 235: The cited article number “34” appears to be incorrect; please verify and correct.

Response: The reference has been corrected

o Line 470: The reference cited here should be checked for accuracy and consistency with the reference list.

Response: Thank you. References 55-58 are accurate and consistent with the information in line 459.

o Lines 485–486: The reference cited to support the authors’ claim in these lines appears inappropriate or not directly supportive of the statement. Please revise the citation or modify the claim to align with the evidence.

Response: Reference 74 has been removed to align with the claim and the remaining references were re-arranged to follow reference 73.

Attachment Submitted filename: Point by Point Response.docx

# Editor report: Decision Letter 2

**DOI:** 10.1371/journal.pone.0351497.r005

**Authors:** Maher Abdelraheim Titi

11 May 2026

PONE-D-25-31196R2

Predictors of Infant Birth Weights: Role of the Lebanese Mediterranean Diet, Psychosocial Factors and Maternal Health Status

PLOS One

Dear Dr. Fares,

Thank you for submitting your manuscript to PLOS ONE. After careful consideration, we feel that it has merit but does not fully meet PLOS ONE’s publication criteria as it currently stands. Therefore, we invite you to submit a revised version of the manuscript that addresses the points raised during the review process.

The authors have successfully resolved and responded to all reviewer comments. Howevre, the manuscript continues to exhibit substantial issues related to reference accuracy, and citation consistency. Kindly see my comments belows.

Please submit your revised manuscript by Jun 25 2026 11:59PM. If you will need more time than this to complete your revisions, please reply to this message or contact the journal office at plosone@plos.org. When you're ready to submit your revision, log on to https://www.editorialmanager.com/pone/ and select the 'Submissions Needing Revision' folder to locate your manuscript file.

Please include the following items when submitting your revised manuscript:

- A letter that responds to each point raised by the academic editor and reviewer(s). You should upload this letter as a separate file labeled 'Response to Reviewers'.
- A marked-up copy of your manuscript that highlights changes made to the original version. You should upload this as a separate file labeled 'Revised Manuscript with Track Changes'.
- An unmarked version of your revised paper without tracked changes. You should upload this as a separate file labeled 'Manuscript'.

If you would like to make changes to your financial disclosure, please include your updated statement in your cover letter. Guidelines for resubmitting your figure files are available below the reviewer comments at the end of this letter.

If applicable, we recommend that you deposit your laboratory protocols in protocols.io to enhance the reproducibility of your results. Protocols.io assigns your protocol its own identifier (DOI) so that it can be cited independently in the future. For instructions see: https://journals.plos.org/plosone/s/submission-guidelines#loc-laboratory-protocols. Additionally, PLOS ONE offers an option for publishing peer-reviewed Lab Protocol articles, which describe protocols hosted on protocols.io. Read more information on sharing protocols at https://plos.org/protocols?utm_medium=editorial-email&utm_source=authorletters&utm_campaign=protocols.

As the corresponding author, your ORCID iD is verified in the submission system and will appear in the published article. PLOS supports the use of ORCID, and we encourage all coauthors to register for an ORCID iD and use it as well. Please encourage your coauthors to verify their ORCID iD within the submission system before final acceptance, as unverified ORCID iDs will not appear in the published article. *Only* the individual author can complete the verification step; PLOS staff *cannot* verify ORCID iDs on behalf of authors.

We look forward to receiving your revised manuscript.

Kind regards,

Maher Abdelraheim Titi

Academic Editor

PLOS One

Journal Requirements:

If the reviewer comments include a recommendation to cite specific previously published works, please review and evaluate these publications to determine whether they are relevant and should be cited. There is no requirement to cite these works unless the editor has indicated otherwise.

Please review your reference list to ensure that it is complete and correct. If you have cited papers that have been retracted, please include the rationale for doing so in the manuscript text, or remove these references and replace them with relevant current references. Any changes to the reference list should be mentioned in the rebuttal letter that accompanies your revised manuscript. If you need to cite a retracted article, indicate the article’s retracted status in the References list and also include a citation and full reference for the retraction notice.

Additional Editor Comments:

The authors have successfully resolved and responded to all reviewer comments. However, the manuscript continues to exhibit substantial issues related to reference accuracy, and citation consistency. The following concerns require careful and systematic correction:

• Missing and Incorrect Citations

o Reference 4 is listed in the reference list but not cited anywhere in the manuscript.

o Reference 54 is also not cited in the text.

o Reference 81 appears to contain an error. Please verify the correct numbering, formatting, and citation placement.

• The manuscript frequently supports individual statements with an unnecessarily large number of references. This practice weakens the clarity and precision of the argument.

Examples include:

o Line 489: Claim supported by seven references [87–93].

o Line 469: Claim supported by eight references [66–73].

o Line 459: Claim supported by six references [59–94].

• Please reduce the number of citations per claim. Supporting a statement with ONE - TWO high quality, recent and relevant studies is generally sufficient unless the claim explicitly requires broader evidence synthesis.

• The overall number of references should be reduced. Several citations appear redundant or marginally relevant. Please ensure that each reference directly contributes to the argument and is essential for supporting the manuscript’s claims.

• The crossed out statement at line 168 should be removed entirely.

[Note: HTML markup is below. Please do not edit.]

To ensure your figures meet our technical requirements, please review our figure guidelines: https://journals.plos.org/plosone/s/figures

You may also use PLOS’s free figure tool, NAAS, to help you prepare publication quality figures: https://journals.plos.org/plosone/s/figures#loc-tools-for-figure-preparation.

NAAS will assess whether your figures meet our technical requirements by comparing each figure against our figure specifications.

# Author comment: Author response to Decision Letter 3

**DOI:** 10.1371/journal.pone.0351497.r006

19 May 2026

Dear Reviewer,

Thank you for your careful review and constructive comments. We have thoroughly revised the manuscript to address all concerns related to references and citation consistency.

Specifically:

• Reference 1 was updated.

• References 4 which was previously listed but not cited in the manuscript, has now been appropriately integrated into the text.

• Reference 54 has been integrated in the text and the new reference number is 44.

• Reference 81 was carefully verified and corrected for numbering, formatting, and citation placement and became number 56.

• We substantially reduced the number of citations supporting individual claims throughout the manuscript. In most cases, statements are now supported by one to two high-quality, recent, and directly relevant references, as recommended.

• The overall reference list was streamlined and reduced to a total of 66 references by removing redundant or marginally relevant citations.

• The crossed-out statement previously appearing at line 168 has been removed entirely from the revised manuscript.

We appreciate the reviewer’s valuable feedback, which helped improve the clarity, precision, and overall quality of the manuscript.

Attachment Submitted filename: Point_by_Point_Response_auresp_3.docx

# Editor report: Decision Letter 3

**DOI:** 10.1371/journal.pone.0351497.r007

**Authors:** Maher Abdelraheim Titi

28 May 2026

Predictors of Infant Birth Weights: Role of the Lebanese Mediterranean Diet, Psychosocial Factors and Maternal Health Status

PONE-D-25-31196R3

Dear Dr. Fares,

We’re pleased to inform you that your manuscript has been judged scientifically suitable for publication and will be formally accepted for publication once it meets all outstanding technical requirements.

Within one week, you’ll receive an e-mail detailing the required amendments. When these have been addressed, you’ll receive a formal acceptance letter and your manuscript will be scheduled for publication.

An invoice will be generated when your article is formally accepted. Please note, if your institution has a publishing partnership with PLOS and your article meets the relevant criteria, all or part of your publication costs will be covered. Please make sure your user information is up-to-date by logging into Editorial Manager at Editorial Manager® and clicking the ‘Update My Information' link at the top of the page. For questions related to billing, please contact billing support.

If your institution or institutions have a press office, please notify them about your upcoming paper to help maximize its impact. If they’ll be preparing press materials, please inform our press team as soon as possible -- no later than 48 hours after receiving the formal acceptance. Your manuscript will remain under strict press embargo until 2 pm Eastern Time on the date of publication. For more information, please contact onepress@plos.org.

Kind regards,

Maher Abdelraheim Titi

Academic Editor

PLOS One

Additional Editor Comments (optional):

Reviewers' comments:

# Editor report: Acceptance letter

**DOI:** 10.1371/journal.pone.0351497.r008

**Authors:** Maher Abdelraheim Titi

PONE-D-25-31196R3

PLOS One

Dear Dr. Fares,

I'm pleased to inform you that your manuscript has been deemed suitable for publication in PLOS One. Congratulations! Your manuscript is now being handed over to our production team.

At this stage, our production department will prepare your paper for publication. This includes ensuring the following:

* All references, tables, and figures are properly cited

* All relevant supporting information is included in the manuscript submission,

* There are no issues that prevent the paper from being properly typeset

You will receive further instructions from the production team, including instructions on how to review your proof when it is ready. Please keep in mind that we are working through a large volume of accepted articles, so please give us a few days to review your paper and let you know the next and final steps.

Lastly, if your institution or institutions have a press office, please let them know about your upcoming paper now to help maximize its impact. If they'll be preparing press materials, please inform our press team within the next 48 hours. Your manuscript will remain under strict press embargo until 2 pm Eastern Time on the date of publication. For more information, please contact onepress@plos.org.

You will receive an invoice from PLOS for your publication fee after your manuscript has reached the completed accept phase. If you receive an email requesting payment before acceptance or for any other service, this may be a phishing scheme. Learn how to identify phishing emails and protect your accounts at https://explore.plos.org/phishing.

If we can help with anything else, please email us at customercare@plos.org.

Thank you for submitting your work to PLOS ONE and supporting open access.

Kind regards,

PLOS ONE Editorial Office Staff

on behalf of

Dr. Maher Abdelraheim Titi

Academic Editor

PLOS One
